# Supplementary material for: Highly Selective Cyclization and Isomerization of Propargylamines to Access Functionalized Quinolines and 1-Azadienes
Source: Molecules. 2023 Aug 26;28(17):6259. doi: 10.3390/molecules28176259 (PMC10488633; doi:10.3390/molecules28176259)
Supplement: Supplementary file 1 [file molecules-28-06259-s001.zip › molecules-2534063-supplementary.pdf]

Supporting Information

Highly Selective Cyclization and Isomerization of Propargylamines to Access Functionalized Quinolines and 1-Azadienes

Zheng-Guang Wu [1,*,†], Hui Zhang [1,†], Chenhui Cao [2], Chaowu Lu [1], Aiwei Jiang [1], Jie He [1], Qin Zhao [1]
and Yanfeng Tang [1]

[1] School of Chemistry and Chemical Engineering, Nantong University, Nantong 226019, China; zhanghui199907@163.com (H.Z.); lu17355389097@163.com (C.L.); jiangaiwei1996@163.com (A.J.); hejie19960901@163.com (J.H.); zhao.q@ntu.edu.cn (Q.Z.); tangyf@ntu.edu.cn (Y.T.)
[2] Anhui Sholon New Material Technology Co., Ltd., Chuzhou 239500, China; caoch@sholonchem.com
* Correspondence: wuzhengguang_zy@163.com
† These authors contributed equally to this work.

1. General information

Commercially available reagents were used as received without purification. Raw Materials were purchased from Bidepharm and Energy-chemical. Column chromatography was carried out on silica gel (200–300 mesh). Analytical thin–layer chromatography was performed on glass plates of Silica Gel GF–254 with detection by UV. ^{1}H, ^{13}C and ^{19}F NMR spectra were recorded on a Bruker AVANCE 400M spectrometer. The chemical shift references were as follows: ^{1}H NMR ($CDCl_3$) 7.26 ppm; ^{13}C NMR ($CDCl_3$) 77.0 ppm. HRMS spectra were carried out on Micromass GCT (ESI). The photoluminescence spectra were measured on a Hitachi F-4600 photoluminescence spectrophotometer. The absolute photoluminescence quantum yields (Φ) was measured with HORIBA FL-3 fluorescence spectrometer.

2. Experimental section

2.1 Synthesis of propargylamines following reported procedures (*J. Org. Chem.* **2006**, *71*, 2064-2070; *Org. Lett.* **2006**, *8*, 2405-2408; *Tetrahedron*, **2014**, *70*, 3134-3140).

2.2 General procedure for the preparation of quinolines through Palladium-catalyzed cyclization.

To a solution of propargylamine (0.1 mmol) and $Pd(OAc)_2$ (5 mol%) in toluene (2 mL), the reaction mixture was stirred at 80 ℃ under air for 12 h. After removing the solvent with vacuum distillation, the crude mixture was purified by flash column chromatography to afford the target product.

2.3 General procedure for the preparation of 1-azadienes *via* Bu_4NOAc-promoted isomerization.

To a solution of propargylamine (0.1 mmol) and Bu_4NOAc (0.2 mmol) in CH_3CN (2 mL), the reaction mixture was stirred at 80 ℃ under air for 12 h. After removing the solvent with vacuum distillation, the crude mixture was purified by flash column chromatography to afford the target product.

2.4 General procedure for the preparation of Ir-2m.

The mixture of **2m** (1.0 mmol) and $IrCl_3$ (0.4 mmol) in 2-ethoxyethanol and water (20 mL, 3 : 1, v/v) was stirred at 130℃ for 24 h under argon. After cooling, the solid precipitate was filtered to obtain crude cyclometalated Ir(III) chloro-bridged dimer. Then, the slurry of crude chloro-bridged dimer, Na_2CO_3 (5.0 mmol) and TMHD (5.0 mmol) in 2-ethoxyethanol (30 mL) was reacted at 120℃ for 24 h. The solvent was evaporated at low pressure, and the mixture was poured into water. Next, the mixture was extracted with CH_2Cl_2 and then chromatographed to give the complex **Ir-2m** with 66% yield.

2.5 General procedure for the preparation of dihydropyridin-2(1H)-ones *via* cycloaddition reaction with 1-azadienes and homophthalic anhydride.

To a solution of propargylamine (1.0 mmol) and Bu_4NOAc (2.0 mmol) in CH_3CN (10 mL), the reaction mixture was stirred at 80 ℃ under air for 12 h. After cooling to room temperature, homophthalic anhydride (1.0 mmol) was added, and the mixture was stirred at room temperature under air for 12 h. After removing the solvent with vacuum distillation, the crude mixture was purified by flash column chromatography to afford the target product.

3. Supplementary data

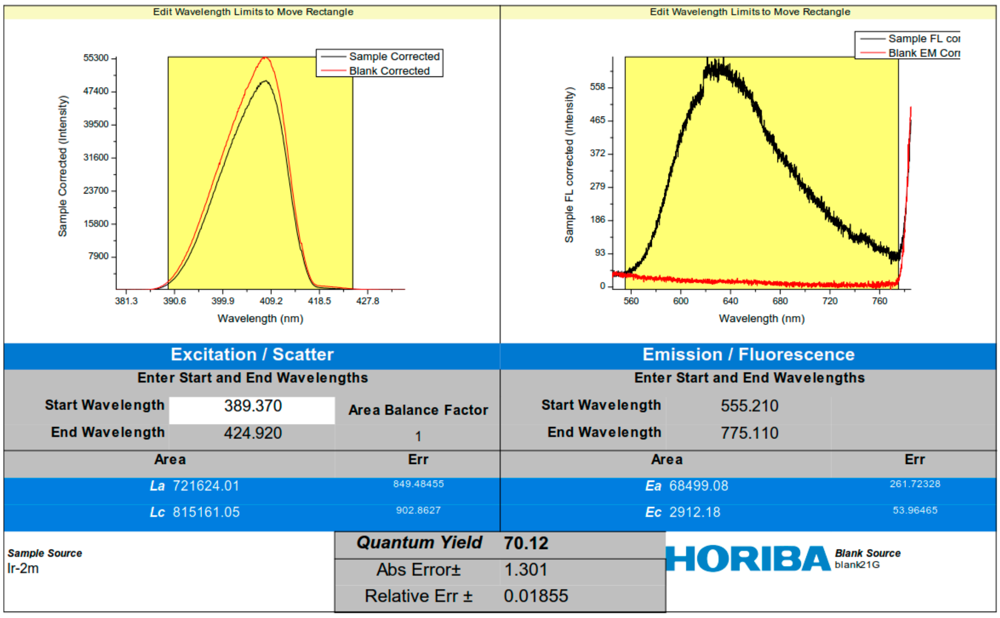

Figure S1 The photoluminescence quantum yield (PLQY) of **Ir-2m**.

4. Characterization data

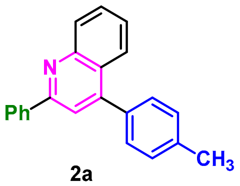

2a

phenyl-4-(p-tolyl)quinoline (2a): White solid, 23.7 mg, 81% yield (Eluent: petroleum ether/ethyl acetate = 50/1).

^{1}H NMR (400 MHz, CDCl$_3$) δ 8.24 (d, *J* = 8.4 Hz, 1H), 8.22 – 8.15 (m, 2H), 7.97 – 7.84 (m, 1H), 7.81 (s, 1H), 7.73 (ddd, *J* = 8.4, 6.8, 1.5 Hz, 1H), 7.57 – 7.39 (m, 6H), 7.36 (d, *J* = 7.8 Hz, 2H), 2.48 (s, 3H).

^{13}C NMR (101 MHz, CDCl$_3$) δ 156.9, 149.3, 148.8, 139.7, 138.4, 135.5, 130.1, 129.5, 129.5, 129.3, 128.9, 128.2, 127.6, 126.3, 125.9, 125.7, 119.4, 21.3.

HRMS (ESI) *m/z* calcd for $C_{22}H_{17}N$ [M+H]: 296.1439, found: 296.1439.

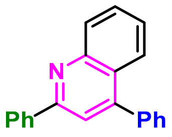

2b

2,4-diphenylquinoline (2b): Light yellow solid, 19.7 mg, 72% yield (Eluent: petroleum ether/ethyl acetate = 50/1).

^{1}H NMR (400 MHz, CDCl$_3$) δ 8.25 (dd, *J* = 8.6, 1.3 Hz, 1H), 8.22 – 8.16 (m, 2H), 7.91 (dd, *J* = 8.5, 1.4 Hz, 1H), 7.82 (s, 1H), 7.74 (ddd, *J* = 8.4, 6.8, 1.5 Hz, 1H), 7.61 – 7.51 (m, 7H), 7.52 – 7.46 (m, 1H), 7.49 – 7.42 (m, 1H).

^{13}C NMR (101 MHz, CDCl$_3$) δ 156.9, 149.2, 148.8, 139.7, 138.4, 130.1, 129.6, 129.5, 129.3, 128.8, 128.6, 128.4, 127.6, 126.3, 125.8, 125.6, 123.5, 119.4, 115.9.

HRMS (ESI) *m/z* calcd for $C_{21}H_{15}N$ [M+H]: 282.1283, found: 282.1283.

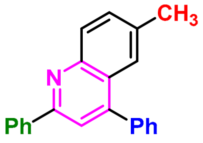

2c

methyl-2,4-diphenylquinoline (2c): Faint yellow solid, 23.4 mg, 78% yield (Eluent: petroleum ether/ethyl acetate = 50/1).

^{1}H NMR (400 MHz, CDCl$_3$) δ 8.21 – 8.10 (m, 3H), 7.78 (s, 1H), 7.65 (s, 1H), 7.56 (d, *J* = 4.4 Hz, 5H), 7.52 (dd, *J* = 8.2, 6.3 Hz, 3H), 7.49 – 7.40 (m, 1H), 2.48 (s, 3H).

^{13}C NMR (101 MHz, CDCl$_3$) δ 156.0, 148.5, 147.4, 139.8, 138.6, 136.3, 131.8, 129.8, 129.6, 129.2, 128.8, 128.6, 128.3, 127.5, 125.7, 124.4, 119.4, 21.8.

HRMS (ESI) *m/z* calcd for $C_{22}H_{17}N$ [M+H]: 296.1439, found: 296.1439.

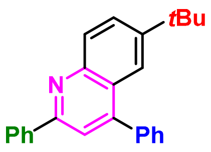

2d

6-(tert-butyl)-2,4-diphenylquinoline (2d): Light yellow solid, 23.6 mg, 70% yield (Eluent: petroleum ether/ethyl acetate = 40/1).

^{1}H NMR (400 MHz, CDCl$_3$) δ 8.23 – 8.13 (m, 3H), 7.90 – 7.76 (m, 3H), 7.63 – 7.39 (m, 8H), 1.35 (s, 9H).

^{13}C NMR (101 MHz, CDCl$_3$) δ 156.3, 149.2, 149.0, 138.6, 129.6, 129.6, 129.2, 128.8, 128.6, 128.4, 128.4, 127.5, 125.3, 120.5, 119.5, 35.1, 31.2.

HRMS (ESI) *m/z* calcd for $C_{25}H_{23}N$ [M+H]: 338.1909, found: 338.1910.

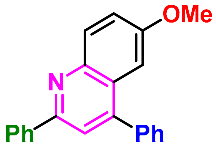

2e

6-methoxy-2,4-diphenylquinoline (2e): Light yellow solid, 24.8 mg, 79% yield (Eluent: petroleum ether/ethyl acetate = 40/1).

^{1}H NMR (400 MHz, CDCl$_3$) δ 8.19 – 8.11 (m, 3H), 7.77 (s, 1H), 7.58 (s, 1H), 7.59 – 7.52 (m, 2H), 7.55 – 7.43 (m, 4H), 7.47 – 7.33 (m, 2H), 7.19 (d, J = 2.8 Hz, 1H), 3.80 (s, 3H).

^{13}C NMR (101 MHz, CDCl$_3$) δ 157.8, 154.7, 147.8, 144.9, 139.8, 138.7, 131.6, 129.4, 129.0, 128.8, 128.7, 128.4, 127.3, 126.7, 121.8, 119.7, 103.7, 55.5.

HRMS (ESI) *m/z* calcd for $C_{22}H_{17}NO$ [M+H]: 312.1388, found: 312.1388.

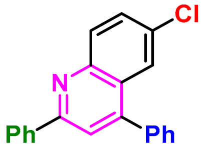

2f

6-chloro-2,4-diphenylquinoline (2f): White solid, 22.3 mg, 73% yield (Eluent: petroleum ether/ethyl acetate = 50/1).

^{1}H NMR (400 MHz, CDCl$_3$) δ 8.22 – 8.14 (m, 3H), 7.89 – 7.82 (m, 2H), 7.67 (dd, J = 9.0, 2.3 Hz, 1H), 7.62 – 7.48 (m, 7H), 7.52 – 7.40 (m, 1H).

^{13}C NMR (101 MHz, CDCl$_3$) δ 157.1, 148.5, 147.2, 139.2, 137.8, 132.2, 131.7, 130.5, 129.6, 129.4, 128.9, 128.8, 128.7, 127.5, 126.5, 124.5, 120.1.

HRMS (ESI) *m/z* calcd for $C_{21}H_{14}ClN$ [M+H]: 316.0893, found: 316.0893.

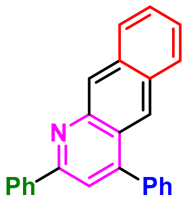

2g

2,4-diphenylbenzo[g]quinoline (2g): Light yellow solid, 23.2 mg, 71% yield (Eluent: petroleum ether/ethyl acetate = 40/1).

^{1}H NMR (400 MHz, CDCl$_3$) δ 8.27 – 8.19 (m, 2H), 8.13 (d, *J* = 9.0 Hz, 1H), 8.00 (d, *J* = 9.0 Hz, 1H), 7.88 (dd, *J* = 7.9, 1.5 Hz, 1H), 7.81 (s, 1H), 7.67 (d, *J* = 8.6 Hz, 1H), 7.60 – 7.42 (m, 9H), 7.16 (ddd, *J* = 8.6, 7.0, 1.5 Hz, 1H).

^{13}C NMR (101 MHz, CDCl$_3$) δ 155.5, 149.8, 149.2, 143.0, 139.1, 132.9, 131.5, 129.8, 129.3, 129.3, 129.2, 128.9, 128.6, 128.4, 128.1, 128.1, 127.4, 126.5, 125.5, 122.8, 121.8.

HRMS (ESI) *m/z* calcd for $C_{25}H_{17}N$ [M+H]: 332.1439, found: 332.1439.

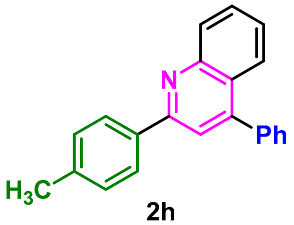

2h

4-phenyl-2-(p-tolyl)quinoline (2h): Faint yellow solid, 20.9 mg, 73% yield (Eluent: petroleum ether/ethyl acetate = 50/1).

^{1}H NMR (400 MHz, CDCl$_3$) δ 8.23 (dd, *J* = 8.6, 1.2 Hz, 1H), 8.13 – 8.06 (m, 2H), 7.89 (dd, *J* = 8.4, 1.4 Hz, 1H), 7.82 – 7.68 (m, 2H), 7.60 – 7.40 (m, 6H), 7.33 (d, *J* = 8.1 Hz, 2H), 2.43 (s, 3H).

^{13}C NMR (101 MHz, CDCl$_3$) δ 156.9, 149.1, 148.8, 139.5, 138.5, 136.9, 130.1, 129.6, 129.5, 128.6, 128.4, 128.1, 127.5, 126.2, 125.7, 125.6, 119.2, 21.4.

HRMS (ESI) *m/z* calcd for $C_{22}H_{17}N$ [M+H]: 296.1439, found: 296.1438.

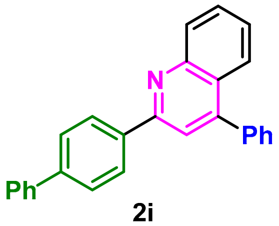

2i

2-([1,1'-biphenyl]-4-yl)-4-phenylquinoline (2i): Light yellow solid, 28.7 mg, 81% yield (Eluent: petroleum ether/ethyl acetate = 40/1).

^{1}H NMR (400 MHz, CDCl$_3$) δ 8.32 – 8.23 (m, 3H), 7.99 – 7.85 (m, 2H), 7.83 – 7.65 (m, 5H), 7.58 (s, 2H), 7.57 – 7.51 (m, 2H), 7.53 – 7.44 (m, 3H), 7.50 – 7.31 (m, 2H).

^{13}C NMR (101 MHz, CDCl$_3$) δ 156.5, 149.2, 148.9, 142.1, 140.6, 138.5, 138.4, 130.1, 129.6, 128.8, 128.6, 128.5, 128.0, 127.6, 127.6, 127.2, 126.4, 125.8, 125.7, 119.3.

HRMS (ESI) *m/z* calcd for $C_{27}H_{19}N$ [M+H]: 358.1596, found: 358.1596.

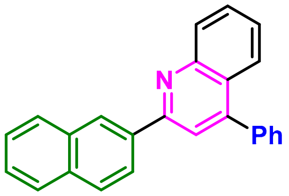

2j

(naphthalen-2-yl)-4-phenylquinoline (2j): Light yellow solid, 23.6 mg, 74% yield (Eluent: petroleum ether/ethyl acetate = 40/1).

^{1}H NMR (400 MHz, CDCl$_3$) δ 8.65 (d, *J* = 1.7 Hz, 1H), 8.41 (dd, *J* = 8.6, 1.8 Hz, 1H), 8.30 (dd, *J* = 8.6, 1.2 Hz, 1H), 8.04 – 7.84 (m, 5H), 7.76 (ddd, *J* = 8.4, 6.8, 1.5 Hz, 1H), 7.67 – 7.45 (m, 8H).

^{13}C NMR (101 MHz, CDCl$_3$) δ 156.7, 149.3, 148.9, 138.5, 136.9, 133.9, 133.5, 130.1, 129.6, 129.5, 128.8, 128.6, 128.6, 128.5, 127.7, 127.2, 126.7, 126.4, 126.3, 125.8, 125.7, 125.1, 124.5, 119.5.

HRMS (ESI) *m/z* calcd for $C_{25}H_{17}N$ [M+H]: 332.1439, found: 332.1439.

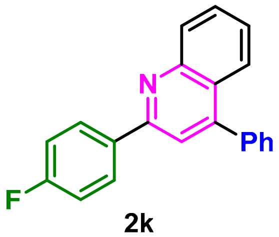

2k

(4-fluorophenyl)-4-phenylquinoline (2k): Faint yellow solid, 23.7 mg, 78% yield (Eluent: petroleum ether/ethyl acetate = 40/1).

^{1}H NMR (400 MHz, CDCl$_3$) δ 8.25 – 8.15 (m, 3H), 7.91 (dd, *J* = 8.4, 1.4 Hz, 1H), 7.80 – 7.70 (m, 2H), 7.56 (s, 3H), 7.60 – 7.50 (m, 2H), 7.48 (ddd, *J* = 8.3, 6.8, 1.3 Hz, 1H), 7.26 – 7.16 (m, 2H).

^{13}C NMR (101 MHz, CDCl$_3$) δ 155.8, 149.4, 148.8, 138.3, 135.8, 130.0, 129.7, 129.6, 129.5, 129.4, 128.7, 128.5, 126.4, 125.7, 119.0, 115.9, 115.7.

^{19}F NMR (376 MHz, CDCl$_3$) δ -112.45.

HRMS (ESI) *m/z* calcd for $C_{21}H_{14}FN$ [M+H]: 300.1189, found: 300.1190.

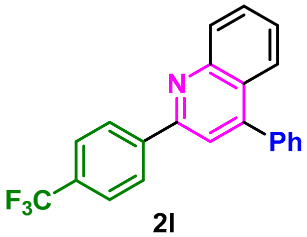

2l

3-phenyl-2-(4-(trifluoromethyl)phenyl)quinoline (2l): Light yellow solid, 24.6 mg, 72% yield (Eluent: petroleum ether/ethyl acetate = 50/1).

^{1}H NMR (400 MHz, CDCl$_3$) δ 8.36 – 8.30 (m, 2H), 8.27 (d, *J* = 8.4 Hz, 1H), 7.94 (dd, *J* = 8.5, 1.4 Hz, 1H), 7.84 (s, 1H), 7.78 (dd, *J* = 8.5, 6.8 Hz, 3H), 7.60 – 7.45 (m,

6H).
^{13}C NMR (101 MHz, $CDCl_3$) δ 138.1, 130.2, 129.9, 129.6, 128.7, 128.6, 127.9, 127.0, 126.1, 125.8, 125.8, 119.2, 29.7.
^{19}F NMR (376 MHz, $CDCl_3$) δ -62.56.
HRMS (ESI) *m/z* calcd for $C_{22}H_{14}F_3N$ [M+H]: 350.1157, found: 350.1156.

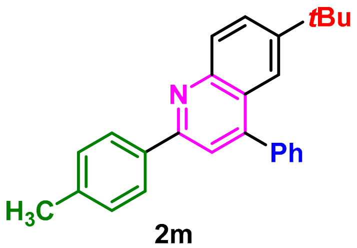

2m

6-(tert-butyl)-4-phenyl-2-(p-tolyl)quinoline (2m): Light yellow solid, 28.3 mg, 82% yield (Eluent: petroleum ether/ethyl acetate = 50/1).
^{1}H NMR (400 MHz, $CDCl_3$) δ 8.16 (d, J = 8.8 Hz, 1H), 8.11 – 8.04 (m, 2H), 7.88 – 7.79 (m, 2H), 7.77 (s, 1H), 7.62 – 7.55 (m, 3H), 7.58 – 7.46 (m, 2H), 7.32 (d, J = 8.0 Hz, 2H), 2.43 (s, 3H), 1.35 (s, 9H).
^{13}C NMR (101 MHz, $CDCl_3$) δ 156.3, 149.0, 148.8, 147.4, 139.2, 138.7, 137.1, 129.6, 129.6, 128.6, 128.3, 127.4, 125.2, 120.5, 119.3, 35.1, 31.2, 21.4.
HRMS (ESI) *m/z* calcd for $C_{26}H_{25}N$ [M+H]: 352.2065, found: 352.2065.

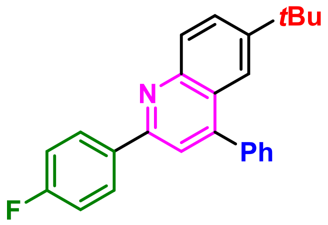

2n

6-(tert-butyl)-2-(4-fluorophenyl)-4-phenylquinoline (2n): Light yellow solid, 24.9 mg, 71% yield (Eluent: petroleum ether/ethyl acetate = 50/1).
^{1}H NMR (400 MHz, $CDCl_3$) δ 8.21 – 8.12 (m, 3H), 7.88 – 7.80 (m, 2H), 7.73 (s, 1H), 7.62 – 7.52 (m, 4H), 7.52 (ddd, J = 6.6, 5.3, 2.6 Hz, 1H), 7.25 – 7.14 (m, 2H), 1.35 (s, 9H).
^{13}C NMR (101 MHz, $CDCl_3$) δ 165.0, 162.5, 155.3, 149.3, 149.2, 147.3, 138.6, 136.0, 136.0, 129.5, 129.4, 129.3, 128.6, 128.6, 128.4, 125.2, 120.6, 119.1, 115.9, 115.6, 35.1, 31.2.
^{19}F NMR (376 MHz, $CDCl_3$) δ -112.78.
HRMS (ESI) *m/z* calcd for $C_{25}H_{22}FN$ [M+H]: 356.1815, found: 356.1815.

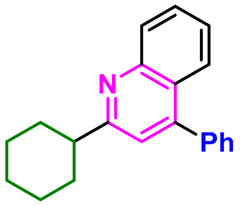

2o

2-cyclohexyl-4-phenylquinoline (2o): White solid, 20.2mg, 81% yield (Eluent: petroleum ether/ethyl acetate = 50/1).

^{1}H NMR (400 MHz, CDCl$_3$) δ 8.10 – 8.01 (m, 1H), 7.79 (dd, *J* = 8.4, 1.4 Hz, 1H), 7.50 – 7.38 (m, 6H), 7.19 (d, *J* = 4.8 Hz, 2H), 2.04 – 1.95 (m, 3H), 1.85 – 1.80 (m, 3H), 1.58 (dd, *J* = 12.4, 3.4 Hz, 2H), 1.42 – 1.37 (m, 2H), 1.18 (s, 1H).

^{13}C NMR (101 MHz, CDCl$_3$) δ 166.4, 148.7, 148.2, 138.5, 129.6, 129.3, 129.2, 128.5, 128.3, 125.7, 125.6, 125.6, 119.9, 47.7, 32.9, 29.7, 26.6, 26.1.

HRMS (ESI) *m/z* calcd for $C_{21}H_{21}N$ [M+H]: 288.1752, found: 288.1752.

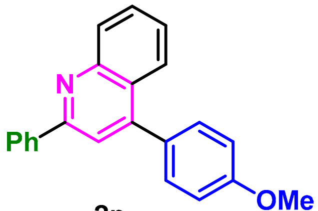

2p

4-(4-methoxyphenyl)-2-phenylquinoline (2p): Light yellow solid, 23.3 mg, 75% yield (Eluent: petroleum ether/ethyl acetate = 50/1).

^{1}H NMR (400 MHz, CDCl$_3$) δ 8.24 (d, *J* = 8.5 Hz, 1H), 8.22 – 8.15 (m, 2H), 7.96 (dd, *J* = 8.4, 1.4 Hz, 1H), 7.80 (s, 1H), 7.73 (ddd, *J* = 8.4, 6.8, 1.5 Hz, 1H), 7.58 – 7.46 (m, 6H), 7.13 – 7.05 (m, 2H), 3.92 (s, 3H).

^{13}C NMR (101 MHz, CDCl$_3$) δ 159.9, 156.9, 148.9, 139.7, 130.8, 130.7, 130.1, 129.5, 129.3, 128.8, 127.6, 126.2, 126.0, 125.7, 119.3, 114.1, 55.4.

HRMS (ESI) *m/z* calcd for $C_{22}H_{17}NO$ [M+H]: 312.1388, found: 312.1388.

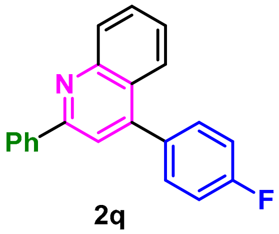

2q

4-(4-fluorophenyl)-2-phenylquinoline (2q): Light yellow solid, 20.9 mg, 71% yield (Eluent: petroleum ether/ethyl acetate = 40/1).

^{1}H NMR (400 MHz, CDCl$_3$) δ 8.26 (dd, *J* = 8.5, 1.2 Hz, 1H), 8.25 – 8.15 (m, 2H), 7.88 (dd, *J* = 8.3, 1.4 Hz, 1H), 7.81 (s, 1H), 7.75 (ddd, *J* = 8.4, 6.8, 1.4 Hz, 1H), 7.58 – 7.41 (m, 5H), 7.35 (dt, *J* = 7.6, 1.3 Hz, 1H), 7.29 (dt, *J* = 9.4, 2.1 Hz, 1H), 7.26 – 7.12 (m, 1H).

^{13}C NMR (101 MHz, CDCl$_3$) δ 164.0, 161.6, 156.9, 148.8, 147.8, 147.8, 140.6, 140.5, 139.5, 130.3, 130.2, 129.8, 129.5, 128.9, 127.6, 126.6, 125.4, 125.4, 125.4, 125.3, 119.3, 116.8, 116.6, 115.5, 115.3.
^{19}F NMR (376 MHz, CDCl$_3$) δ -112.48.
HRMS (ESI) *m/z* calcd for $C_{21}H_{14}FN$ [M+H]: 300.1189, found: 300.1189.

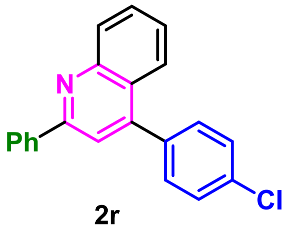

2r

4-(4-chlorophenyl)-2-phenylquinoline (2r): Light yellow solid, 22.2 mg, 71% yield (Eluent: petroleum ether/ethyl acetate = 50/1).
^{1}H NMR (400 MHz, CDCl$_3$) δ 8.25 (d, J = 8.4 Hz, 1H), 8.22 – 8.15 (m, 2H), 7.85 (dd, J = 8.4, 1.4 Hz, 1H), 7.81 – 7.65 (m, 2H), 7.58 – 7.43 (m, 8H).
^{13}C NMR (101 MHz, CDCl$_3$) δ 156.9, 148.8, 147.9, 139.5, 136.8, 134.7, 130.9, 130.3, 129.7, 129.5, 128.9, 127.6, 126.6, 125.5, 125.3, 119.3.
HRMS (ESI) *m/z* calcd for $C_{21}H_{14}ClN$ [M+H]: 316.0893, found: 316.0893.

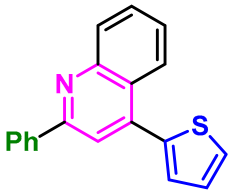

2s

2-phenyl-4-(thiophen-2-yl)quinoline (2s): Light yellow solid, 20.3 mg, 72% yield (Eluent: petroleum ether/ethyl acetate = 40/1).
^{1}H NMR (400 MHz, CDCl$_3$) δ 8.26 (ddd, J = 14.2, 8.5, 1.4 Hz, 2H), 8.21 – 8.14 (m, 2H), 7.92 (s, 1H), 7.75 (ddd, J = 8.3, 6.8, 1.4 Hz, 1H), 7.59 – 7.40 (m, 6H), 7.25 (t, J = 4.3 Hz, 1H).
^{13}C NMR (101 MHz, CDCl$_3$) δ 156.9, 149.0, 141.5, 139.4, 139.2, 130.2, 129.7, 129.4, 128.9, 128.5, 127.8, 127.6, 127.2, 126.7, 125.4, 125.3, 119.8.
HRMS (ESI) *m/z* calcd for $C_{19}H_{13}NS$ [M+H]: 288.0847, found: 288.0847.

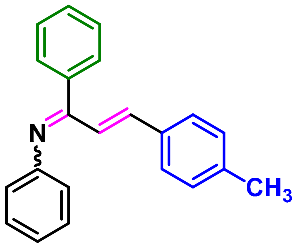

3a

(2E)-N,1-diphenyl-3-(p-tolyl)prop-2-en-1-imine (3a): Light yellow solid, 26.8 mg, 91% yield (Eluent: petroleum ether/ethyl acetate = 40/1).

^{1}H NMR (400 MHz, CDCl$_3$) δ 7.78 – 7.69 (m, 2H), 7.47 (dd, *J* = 4.1, 1.8 Hz, 2H), 7.38 – 7.34 (m, 2H), 7.22 – 7.08 (m, 6H), 6.93 – 6.85 (m, 2H), 2.34 (d, *J* = 11.3 Hz, 3H).

^{13}C NMR (101 MHz, CDCl$_3$) δ 167.3 (C=N), 151.0, 141.7, 132.9, 129.8, 129.5, 129.5, 129.4, 128.8, 128.3, 127.5, 127.5, 123.8, 120.8, 21.4.

HRMS (ESI) *m/z* calcd for $C_{22}H_{19}N$ [M+H]: 298.1596, found: 298.1594.

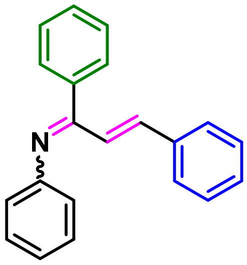

3b

(2E)-N,1,3-triphenylprop-2-en-1-imine(3b): Light yellow solid, 25.3 mg, 88% yield (Eluent: petroleum ether/ethyl acetate = 40/1).

^{1}H NMR (400 MHz, CDCl$_3$) δ 7.75 (t, *J* = 2.0 Hz, 1H), 7.49 (q, *J* = 1.4 Hz, 3H), 7.37 (s, 1H), 7.30 (d, *J* = 9.6 Hz, 7H), 7.13 – 7.11 (m, 1H), 6.97 (d, *J* = 1.3 Hz, 1H), 6.92 (s, 1H).

^{13}C NMR (101 MHz, CDCl$_3$) δ 167.2 (C=N), 150.9, 141.7, 139.4, 135.7, 131.6, 129.4, 129.4, 128.9, 128.8, 128.4, 127.5, 124.0, 121.9, 120.8.

HRMS (ESI) *m/z* calcd for $C_{21}H_{17}N$ [M+H]: 284.1439, found: 284.1441.

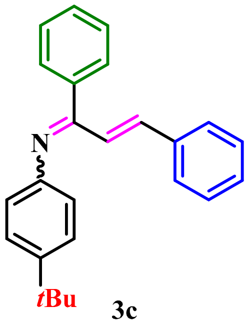

3c

(2E)-N-(4-(tert-butyl)phenyl)-1,3-diphenylprop-2-en-1-imine (3c): Light yellow solid, 28.3 mg, 85% yield (Eluent: petroleum ether/ethyl acetate = 30/1).

^{1}H NMR (400 MHz, CDCl$_3$) δ 7.74 (t, *J* = 2.0 Hz, 1H), 7.48 (t, *J* = 1.7 Hz, 2H), 7.39 – 7.30 (m, 8H), 6.98 – 6.92 (m, 3H), 1.36 (s, 9H).

^{13}C NMR (101 MHz, CDCl$_3$) δ 166.9 (C=N), 148.1, 146.9, 141.3, 139.7, 135.9, 132.0, 129.8, 129.4, 128.8, 128.3, 127.5, 125.7, 122.3, 120.8, 34.4, 31.5.

HRMS (ESI) *m/z* calcd for $C_{25}H_{25}N$ [M+H]: 340.2065, found: 340.2063.

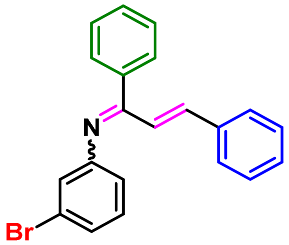

3d

(2E)-N-(3-bromophenyl)-1,3-diphenylprop-2-en-1-imine (3d): Light yellow solid, 32.7 mg, 92% yield (Eluent: petroleum ether/ethyl acetate = 40/1).

^{1}H NMR (400 MHz, CDCl$_3$) δ 7.71 (d, J = 2.0 Hz, 2H), 7.50 (s, 3H), 7.34 – 7.29 (m, 2H), 7.14 (t, J = 2.1 Hz, 2H), 6.94 (s, 2H), 6.86 (d, J = 2.6 Hz, 3H).

^{13}C NMR (101 MHz, CDCl$_3$) δ 168.0 (C=N), 152.4, 142.7, 138.9, 135.4, 131.1, 130.3, 129.3, 128.9, 128.4, 127.6, 126.8, 124.1, 123.6, 122.7, 121.3, 119.3.

HRMS (ESI) *m/z* calcd for $C_{21}H_{16}BrN$ [M+H]: 362.0544, found: 362.0546.

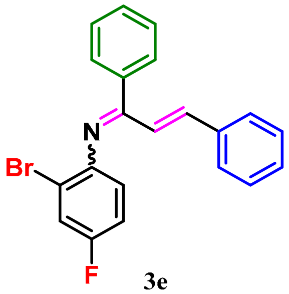

3e

(2E)-N-(2-bromo-4-fluorophenyl)-1,3-diphenylprop-2-en-1-imine (3e): Light yellow solid, 34.2 mg, 91% yield (Eluent: petroleum ether/ethyl acetate = 40/1).

^{1}H NMR (400 MHz, CDCl$_3$) δ 7.80 (t, J = 1.6 Hz, 2H), 7.50 (d, J = 7.0 Hz, 5H), 7.32 (d, J = 11.7 Hz, 1H), 7.24 (d, J = 11.8 Hz, 2H), 6.95 (s, 1H), 6.87 – 6.84 (m, 1H), 6.73 (s, 1H).

^{13}C NMR (101 MHz, CDCl$_3$) δ 169.3 (C=N), 157.6, 142.9, 138.5, 135.4, 130.7, 130.2, 129.8, 129.3, 128.9, 128.4, 128.2, 127.7, 121.5, 121.1, 119.9, 115.1, 114.9.

^{19}F NMR (376 MHz, CDCl$_3$) δ -118.89.

HRMS (ESI) *m/z* calcd for $C_{21}H_{15}BrFN$ [M+H]: 380.0450, found: 380.0453.

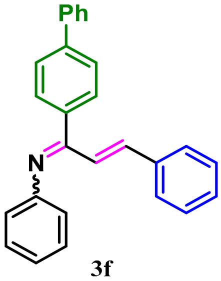

3f

(2E)-1-([1,1'-biphenyl]-4-yl)-N,3-diphenylprop-2-en-1-imine (3f): Light yellow solid, 32.1mg, 87% yield (Eluent: petroleum ether/ethyl acetate = 40/1).

^{1}H NMR (400 MHz, CDCl$_3$) δ 7.90 – 7.80 (m, 1H), 7.77 – 7.57 (m, 4H), 7.59 – 7.48 (m, 1H), 7.50 – 7.38 (m, 3H), 7.42 – 7.32 (m, 3H), 7.35 – 7.28 (m, 3H), 7.21 – 7.12 (m, 1H), 7.06 – 6.88 (m, 3H).
^{13}C NMR (101 MHz, CDCl$_3$) δ 166.8 (C=N), 150.9, 144.8, 142.8, 141.6, 140.5, 139.9, 135.7, 129.9, 129.4, 128.9, 128.8, 128.2, 127.5, 127.2, 127.1, 124.0, 122.0, 120.9.
HRMS (ESI) *m/z* calcd for $C_{27}H_{21}N$ [M+H]: 360.1752, found: 360.1754.

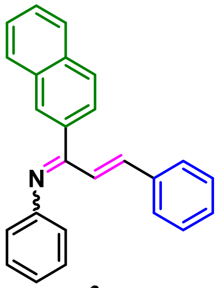

3g

(2E)-1-(naphthalen-2-yl)-N,3-diphenylprop-2-en-1-imine (3g): Light yellow solid, 28.2 mg, 84% yield (Eluent: petroleum ether/ethyl acetate = 30/1).
^{1}H NMR (400 MHz, CDCl$_3$) δ 8.24 (d, *J* = 1.7 Hz, 1H), 7.96 – 7.91 (m, 3H), 7.71 – 7.68 (m, 1H), 7.40 – 7.24 (m, 9H), 6.99 (d, *J* = 7.6 Hz, 3H).
^{13}C NMR (101 MHz, CDCl$_3$) δ 167.1 (C=N), 151.0, 144.8, 141.7, 136.8, 135.7, 134.1, 129.4, 128.9, 128.8, 128.8, 127.7, 127.5, 126.4, 124.0, 122.2, 120.8.
HRMS (ESI) *m/z* calcd for C25H19N [M+H]: 334.1596, found: 334.1593.

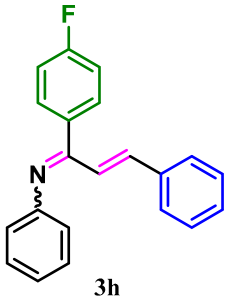

3h

(2E)-1-(4-fluorophenyl)-N,3-diphenylprop-2-en-1-imine (3h): Light yellow solid,27.4 mg, 93% yield (Eluent: petroleum ether/ethyl acetate = 40/1).
^{1}H NMR (400 MHz, CDCl$_3$) δ 7.76 (d, *J* = 3.4 Hz, 1H), 7.37 – 7.24 (m, 6H), 7.16 (d, *J* = 1.7 Hz, 3H), 6.94 (t, *J* = 1.1 Hz, 2H), 6.89 (s, 2H).
^{13}C NMR (101 MHz, CDCl$_3$) δ 166.0 (C=N), 150.8, 141.6, 135.5, 131.4, 131.3, 129.5, 128.9, 128.9, 127.5, 124.1, 121.9, 120.8, 115.5, 115.3.
^{19}F NMR (376 MHz, CDCl$_3$) δ -110.92.
HRMS (ESI) *m/z* calcd for $C_{21}H_{16}FN$ [M+H]: 302.1345, found: 302.1347.

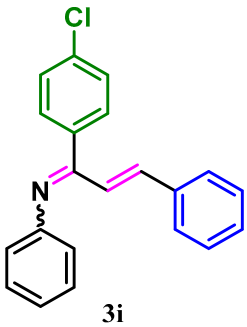

3i

(2E)-1-(4-chlorophenyl)-N,3-diphenylprop-2-en-1-imine (3i): Light yellow solid, 28.5 mg, 90% yield (Eluent: petroleum ether/ethyl acetate = 40/1).

^{1}H NMR (400 MHz, CDCl$_3$) δ 7.65 – 7.59 (m, 2H), 7.46 – 7.23 (m, 6H), 7.21 – 7.15 (m, 1H), 7.07 (dt, *J* = 6.0, 2.6 Hz, 1H), 6.91 – 6.79 (m, 4H).

^{13}C NMR (101 MHz, CDCl$_3$) δ 165.0 (C=N), 149.6, 140.6, 136.8, 135.0, 134.4, 129.7, 129.4, 128.6, 127.9, 127.8, 127.6, 126.5, 123.1, 119.7.

HRMS (ESI) *m/z* calcd for $C_{21}H_{16}ClN$ [M+H]: 318.1050, found: 318.1046.

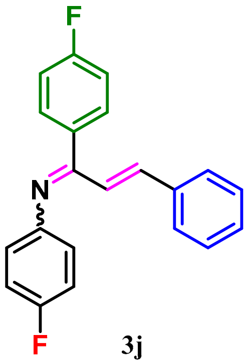

3j

(2E)-N,1-bis(4-fluorophenyl)-3-phenylprop-2-en-1-imine (3j): Light yellow solid, 25.5 mg, 81% yield (Eluent: petroleum ether/ethyl acetate = 30/1).

^{1}H NMR (400 MHz, CDCl$_3$) δ 8.11 – 8.02 (m, 1H), 7.76 – 7.73 (m, 1H), 7.42 (d, *J* = 3.0 Hz, 1H), 7.33 (s, 4H), 7.22 – 7.15 (m, 2H), 7.10 – 6.99 (m, 2H), 6.90 (t, *J* = 6.1 Hz, 2H).

^{13}C NMR (101 MHz, CDCl$_3$) δ 166.7 (C=N), 146.8, 141.9, 135.4, 134.8, 131.3, 130.7, 129.7, 128.9, 127.5, 122.2, 121.7, 115.8, 115.5, 115.3.

^{19}F NMR (376 MHz, CDCl$_3$) δ -110.63, -119.57.

HRMS (ESI) *m/z* calcd for $C_{21}H_{15}F_2N$ [M+H]: 320.1251, found: 320.1253.

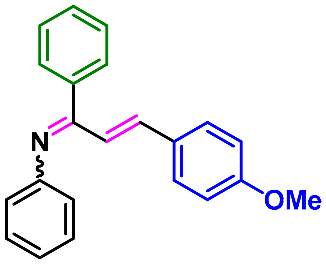

3k

(2E)-3-(4-methoxyphenyl)-N,1-diphenylprop-2-en-1-imine (3k): Light yellow solid, 28.3 mg, 92% yield (Eluent: petroleum ether/ethyl acetate = 30/1).

^{1}H NMR (400 MHz, $CDCl_3$) δ 7.74 – 7.69 (m, 2H), 7.52 – 7.47 (m, 3H), 7.36 (t, *J* = 7.8 Hz, 2H), 7.28 – 7.24 (m, 2H), 7.16 – 7.11 (m, 2H), 6.87 – 6.80 (m, 3H), 3.79 (s, 3H).

^{13}C NMR (101 MHz, $CDCl_3$) δ 167.5 (C=N), 160.7, 151.0, 141.5, 139.5, 129.7, 129.4, 129.0, 128.8, 128.6, 128.3, 123.8, 120.8, 119.7, 114.2, 55.3.

HRMS (ESI) *m/z* calcd for $C_{22}H_{19}NO$ [M+H]: 314.1545, found: 313.2672.

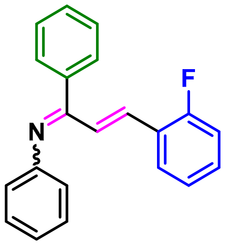

3l

(2E)-3-(2-fluorophenyl)-N,1-diphenylprop-2-en-1-imine (3l): Light yellow solid, 27.0 mg, 89% yield (Eluent: petroleum ether/ethyl acetate = 40/1).

^{1}H NMR (400 MHz, $CDCl_3$) δ 7.78 – 7.70 (m, 1H), 7.54 – 7.46 (m, 2H), 7.42 – 7.35 (m, 2H), 7.27 (dq, *J* = 13.9, 3.2 Hz, 2H), 7.19 – 7.04 (m, 3H), 6.98 (ddd, *J* = 16.0, 8.7, 1.2 Hz, 3H), 6.88 (s, 1H).

^{13}C NMR (101 MHz, $CDCl_3$) δ 166.7 (C=N), 150.7, 140.2, 137.9, 133.0, 130.3, 130.3, 129.3, 128.9, 128.4, 124.2, 123.1, 120.7, 116.3, 116.1, 113.9, 113.7.

HRMS (ESI) *m/z* calcd for $C_{21}H_{16}FN$ [M+H]: 302.1345, found: 302.1342.

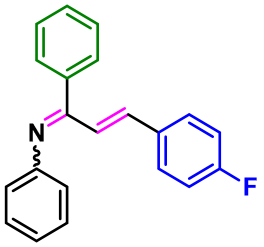

3m

(2E)-3-(4-fluorophenyl)-N,1-diphenylprop-2-en-1-imine (3m): Light yellow solid, 26.8 mg, 86% yield (Eluent: petroleum ether/ethyl acetate = 40/1).

^{1}H NMR (400 MHz, $CDCl_3$) δ 7.76 – 7.70 (m, 1H), 7.56 – 7.44 (m, 2H), 7.41 – 7.21 (m, 4H), 7.20 – 7.04 (m, 3H), 7.02 – 6.86 (m, 4H).

^{13}C NMR (101 MHz, $CDCl_3$) δ 166.7 (C=N), 161.8, 150.7, 140.2, 137.9, 132.9, 130.0, 129.3, 128.9, 128.9, 128.4, 123.1, 120.7, 116.1, 113.7.

^{19}F NMR (376 MHz, $CDCl_3$) δ -112.69.

HRMS (ESI) *m/z* calcd for $C_{21}H_{16}FN$ [M+H]: 302.1345, found: 302.1348.

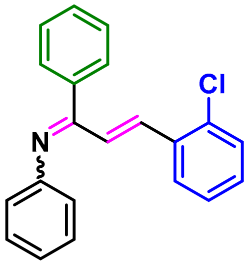

3n

(2E)-3-(2-chlorophenyl)-N,1-diphenylprop-2-en-1-imine (3n): Light yellow solid, 28.1 mg, 86% yield (Eluent: petroleum ether/ethyl acetate = 40/1).
^{1}H NMR (400 MHz, CDCl$_3$) δ 7.83 – 7.72 (m, 2H), 7.51 – 7.49 (m, 2H), 7.38 – 7.35 (m, 3H), 7.29 – 7.23 (m, 2H), 7.17 – 7.12 (m, 2H), 7.00 – 6.94 (m, 2H), 6.84 (d, J = 16.5 Hz, 1H).
^{13}C NMR (101 MHz, CDCl$_3$) δ 166.8 (C=N), 150.9, 139.0, 137.5, 134.3, 134.1, 130.2, 129.9, 129.4, 128.9, 128.6, 128.4, 127.3, 127.0, 124.4, 124.1, 120.7.
HRMS (ESI) *m/z* calcd for $C_{21}H_{16}ClN$ [M+H]: 318.1050, found: 318.1042.

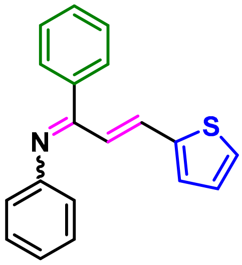

3o

(2E)-N,1-diphenyl-3-(thiophen-2-yl)prop-2-en-1-imine (3o): Light yellow solid, 24.6 mg, 85% yield (Eluent: petroleum ether/ethyl acetate = 40/1).
^{1}H NMR (400 MHz, CDCl$_3$) δ 7.76 – 7.66 (m, 1H), 7.52 – 7.43 (m, 2H), 7.41 – 7.23 (m, 3H), 7.18 – 6.83 (m, 6H), 6.74 – 6.62 (m, 1H).
^{13}C NMR (101 MHz, CDCl$_3$) δ 166.8 (C=N), 150.8, 141.0, 139.2, 137.2, 134.4, 132.8, 132.1, 129.3, 128.8, 128.4, 124.0, 121.0, 120.8.
HRMS (ESI) *m/z* calcd for $C_{19}H_{15}NS$ [M+H]: 290.1003, found: 290.1001.

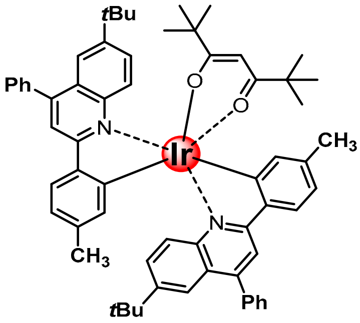

Ir-2m

Ir-2m: red solid, 710.4 mg, 66% yield (Eluent: petroleum ether/dichloromethane = 10/1).

^{1}H NMR (400 MHz, $CDCl_3$) δ 8.19 (d, *J* = 9.2 Hz, 2H), 7.84 (s, 2H), 7.68 – 7.59 (m, 4H), 7.58 – 7.45 (m, 10H), 7.29 (dd, *J* = 9.2, 2.3 Hz, 2H), 6.68 (d, *J* = 7.9 Hz, 2H), 6.55 (s, 2H), 4.75 (s, 1H), 1.96 (s, 6H), 1.18 (s, 18H), 0.56 (s, 18H).
^{13}C NMR (101 MHz, $CDCl_3$) δ 192.5, 167.7, 150.9, 148.2, 146.8, 146.8, 143.9, 137.5, 137.0, 136.5, 128.6, 127.6, 127.6, 127.4, 125.9, 123.8, 123.7, 120.7, 119.2, 115.7, 39.5, 33.7, 30.0, 26.8, 20.6.
HRMS (ESI) *m/z* calcd for $C_{63}H_{67}IrN_2O_2$ [M+H]: 1077.4910, found: 1077.4910.

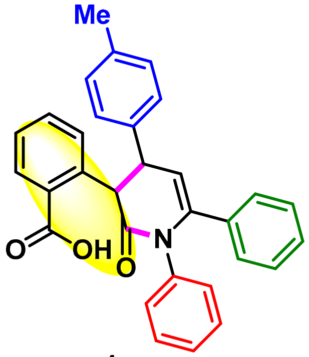

4a

4a: Light yellow solid, 358.1 mg, 78% yield (Eluent: dichloromethane/methyl alcohol = 100/1).
^{1}H NMR (400 MHz, DMSO) δ 13.07 (s, 1H), 7.84 (d, *J* = 7.7 Hz, 1H), 7.43 (t, *J* = 7.5 Hz, 1H), 7.38 (s, 1H), 7.34 – 7.16 (m, 10H), 7.15 (d, *J* = 4.2 Hz, 2H), 7.15 – 7.07 (m, 1H), 7.06 (d, *J* = 7.6 Hz, 2H), 5.58 (d, *J* = 4.3 Hz, 1H), 5.11 (d, *J* = 8.9 Hz, 1H), 4.35 (dd, *J* = 8.7, 4.2 Hz, 1H), 2.23 (s, 3H).
^{13}C NMR (101 MHz, DMSO) δ 170.4, 169.3, 141.9, 139.4, 139.3, 136.4, 136.2, 132.0, 131.4, 131.1, 129.5, 128.7, 128.6, 128.5, 128.2, 128.1, 128.0, 127.4, 126.8, 115.2, 44.5, 21.0.
HRMS (ESI) *m/z* calcd for $C_{31}H_{25}NO_3$ [M+H]: 460.1913, found: 460.1914.

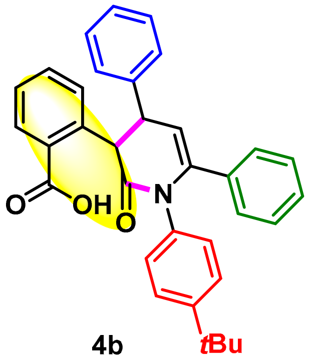

4b

4b: Light yellow solid, 364.4 mg, 80% yield (Eluent: dichloromethane/methyl alcohol = 100/1).
^{1}H NMR (400 MHz, DMSO) δ 12.90 (s, 1H), 7.83 (dd, *J* = 7.7, 1.5 Hz, 1H), 7.42 (d, *J* = 1.6 Hz, 1H), 7.35 – 7.27 (m, 5H), 7.30 – 7.21 (m, 5H), 7.23 – 7.12 (m, 4H), 7.14 – 7.07 (m, 2H), 5.61 (d, *J* = 4.3 Hz, 1H), 5.12 (d, *J* = 9.1 Hz, 1H), 4.39 (dd, *J* = 9.2, 4.3 Hz, 1H), 1.19 (s, 9H).

^{13}C NMR (101 MHz, DMSO) δ 170.5, 169.3, 149.0, 142.4, 142.0, 139.4, 136.7, 136.5, 132.0, 131.1, 131.1, 128.9, 128.5, 128.3, 128.2, 128.2, 127.9, 127.4, 127.2, 125.3, 115.1, 45.0, 34.6, 31.5.
HRMS (ESI) *m/z* calcd for $C_{34}H_{31}NO_3$ [M+H]: 502.2382, found: 502.2381.

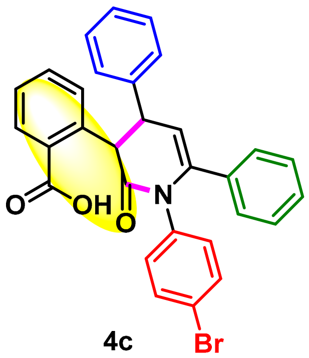

4c

4c: Light yellow solid, 403.7 mg, 77% yield (Eluent: dichloromethane/methyl alcohol = 100/1).
^{1}H NMR (400 MHz, DMSO) δ 13.05 (s, 1H), 7.84 (dd, *J* = 8.0, 1.5 Hz, 1H), 7.47 – 7.36 (m, 3H), 7.34 – 7.27 (m, 4H), 7.30 – 7.19 (m, 6H), 7.22 – 7.11 (m, 4H), 5.63 (d, *J* = 4.0 Hz, 1H), 5.11 (d, *J* = 9.7 Hz, 1H), 4.44 (dd, *J* = 9.7, 4.0 Hz, 1H).
^{13}C NMR (101 MHz, DMSO) δ 170.4, 169.2, 142.4, 141.4, 139.3, 138.8, 136.2, 132.0, 131.6, 131.2, 131.1, 130.9, 128.9, 128.6, 128.4, 128.3, 128.1, 127.4, 127.3, 119.5, 115.6, 45.0.
HRMS (ESI) *m/z* calcd for $C_{30}H_{22}BrNO_3$ [M+H]: 524.0861, found: 524.0860.

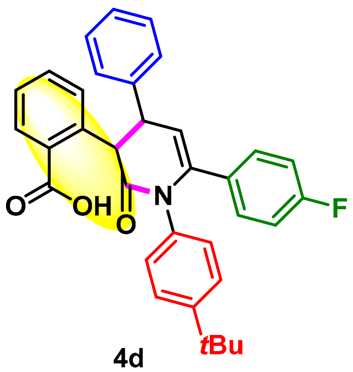

4d

4d: White solid, 374.7 mg, 74% yield (Eluent: dichloromethane/methyl alcohol = 100/1).
^{1}H NMR (400 MHz, DMSO) δ 13.10 (s, 1H), 7.84 (d, *J* = 7.7 Hz, 1H), 7.43 – 7.33 (m, 3H), 7.28 (dd, *J* = 14.6, 8.5 Hz, 8H), 7.18 (t, *J* = 7.1 Hz, 1H), 7.11 (d, *J* = 8.1 Hz, 2H), 7.02 (t, *J* = 8.6 Hz, 2H), 5.61 (d, *J* = 4.2 Hz, 1H), 5.10 (d, *J* = 9.2 Hz, 1H), 4.45 – 4.37 (m, 1H), 1.20 (s, 9H).
^{13}C NMR (101 MHz, DMSO) δ 170.3, 169.3, 163.0, 160.6, 149.2, 142.4, 141.0, 139.4, 136.6, 133.0, 132.9, 132.0, 131.2, 131.1, 130.2, 130.1, 128.9, 128.3, 128.2, 127.4, 127.2, 125.4, 115.5, 115.3, 115.1, 45.0, 34.7, 31.5.
^{19}F NMR (376 MHz, DMSO) δ -113.86.

HRMS (ESI) *m/z* calcd for $C_{34}H_{30}FNO_3$ [M+H]: 520.2288, found: 520.2286.

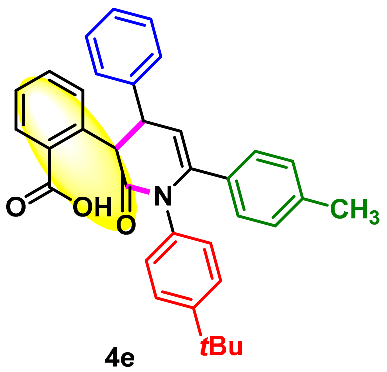

4e

4e: Light yellow solid, 408.1 mg, 79% yield (Eluent: dichloromethane/methyl alcohol = 100/1).

^{1}H NMR (400 MHz, DMSO) δ 13.07 (s, 1H), 7.83 (dd, *J* = 7.8, 1.4 Hz, 1H), 7.46 – 7.23 (m, 9H), 7.19 (dd, *J* = 10.5, 7.6 Hz, 3H), 7.10 (d, *J* = 8.3 Hz, 2H), 7.00 (d, *J* = 7.9 Hz, 2H), 5.57 (d, *J* = 4.4 Hz, 1H), 5.11 (d, *J* = 8.9 Hz, 1H), 4.35 (dd, *J* = 9.0, 4.4 Hz, 1H), 2.18 (s, 3H), 1.20 (s, 9H).

^{13}C NMR (101 MHz, DMSO) δ 170.5, 169.3, 149.0, 141.9, 139.4, 137.5, 136.8, 133.6, 132.0, 131.2, 129.1, 128.9, 128.2, 128.1, 127.8, 127.4, 127.2, 125.4, 114.6, 44.9, 34.7, 31.5, 21.1.

HRMS (ESI) *m/z* calcd for $C_{35}H_{33}NO_3$ [M+H]: 516.2539, found: 516.2541.

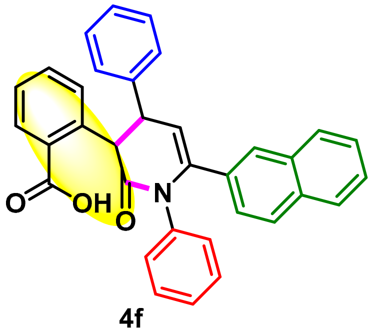

4f

4f: Light yellow solid, 384.3 mg, 78% yield (Eluent: dichloromethane/methyl alcohol = 100/1).

^{1}H NMR (400 MHz, DMSO) δ 13.12 (s, 1H), 7.95 (s, 1H), 7.84 (dd, *J* = 12.0, 6.7 Hz, 2H), 7.81 – 7.75 (m, 1H), 7.68 (d, *J* = 8.6 Hz, 1H), 7.50 – 7.15 (m, 15H), 7.04 (t, *J* = 7.3 Hz, 1H), 5.76 (d, *J* = 4.2 Hz, 1H), 5.20 (d, *J* = 9.5 Hz, 1H), 4.49 (dd, *J* = 9.6, 4.2 Hz, 1H).

^{13}C NMR (101 MHz, DMSO) δ 170.4, 169.3, 142.5, 141.9, 139.4, 139.4, 134.0, 133.0, 132.6, 132.0, 131.2, 131.1, 129.0, 128.8, 128.6, 128.4, 128.3, 127.8, 127.7, 127.4, 127.3, 127.1, 126.9, 126.8, 125.8, 115.7, 45.1.

HRMS (ESI) *m/z* calcd for $C_{34}H_{25}NO_3$ [M+H]: 496.1913, found: 496.1916.

5. ^{1}H, ^{13}C and ^{19}F NMR Spectra

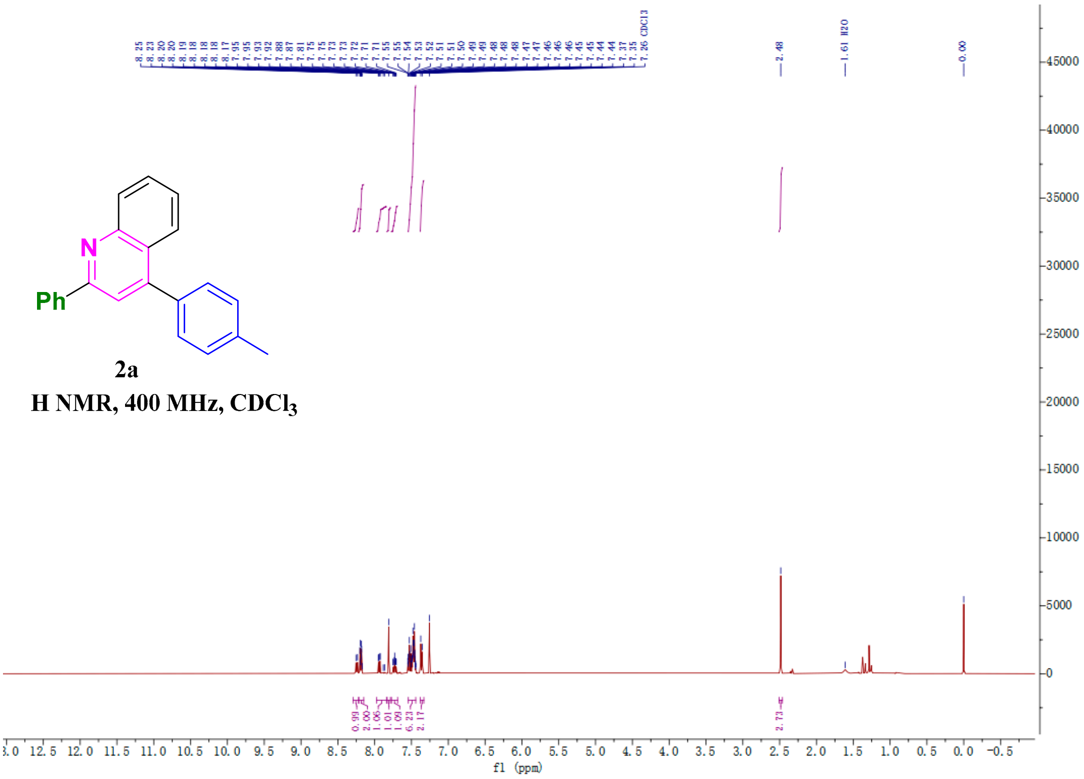

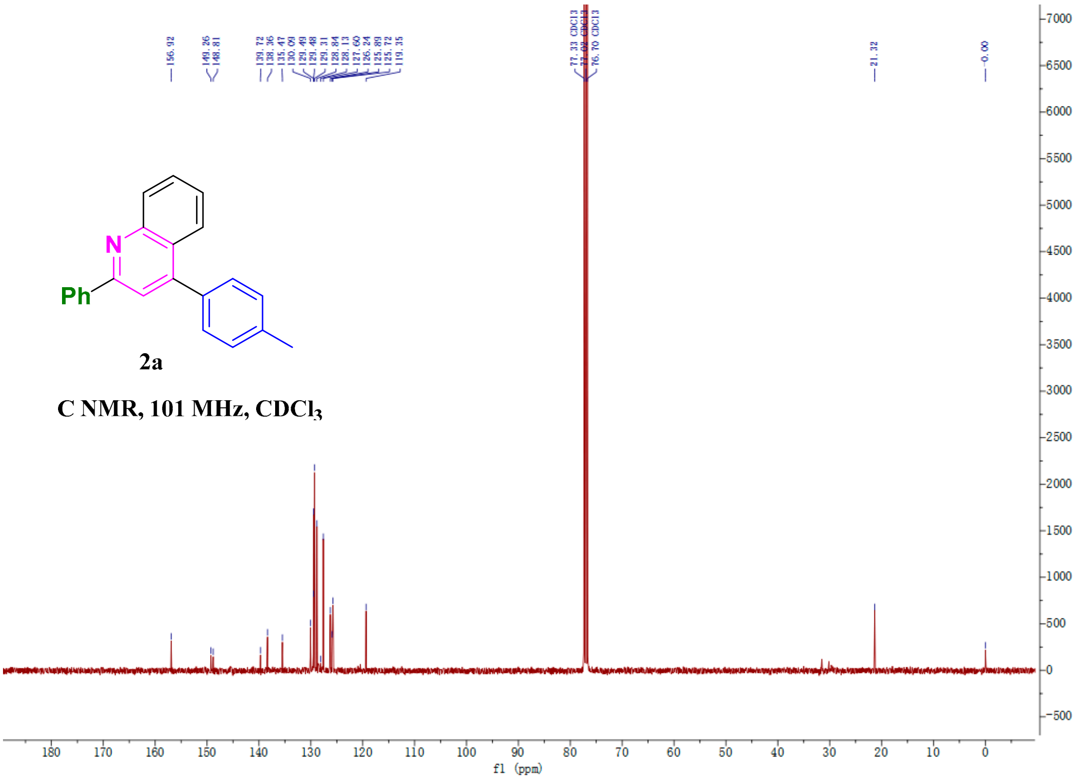

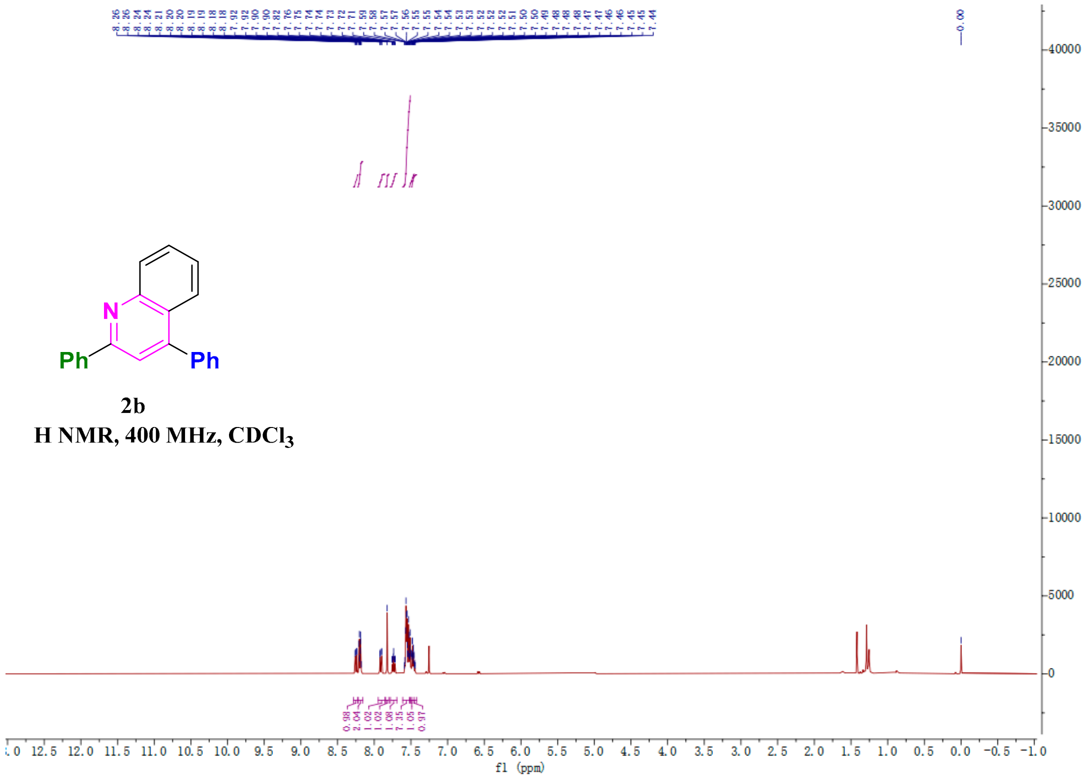
N
Ph
Ph
2b
H NMR, 400 MHz, CDCl3
f1 (ppm)

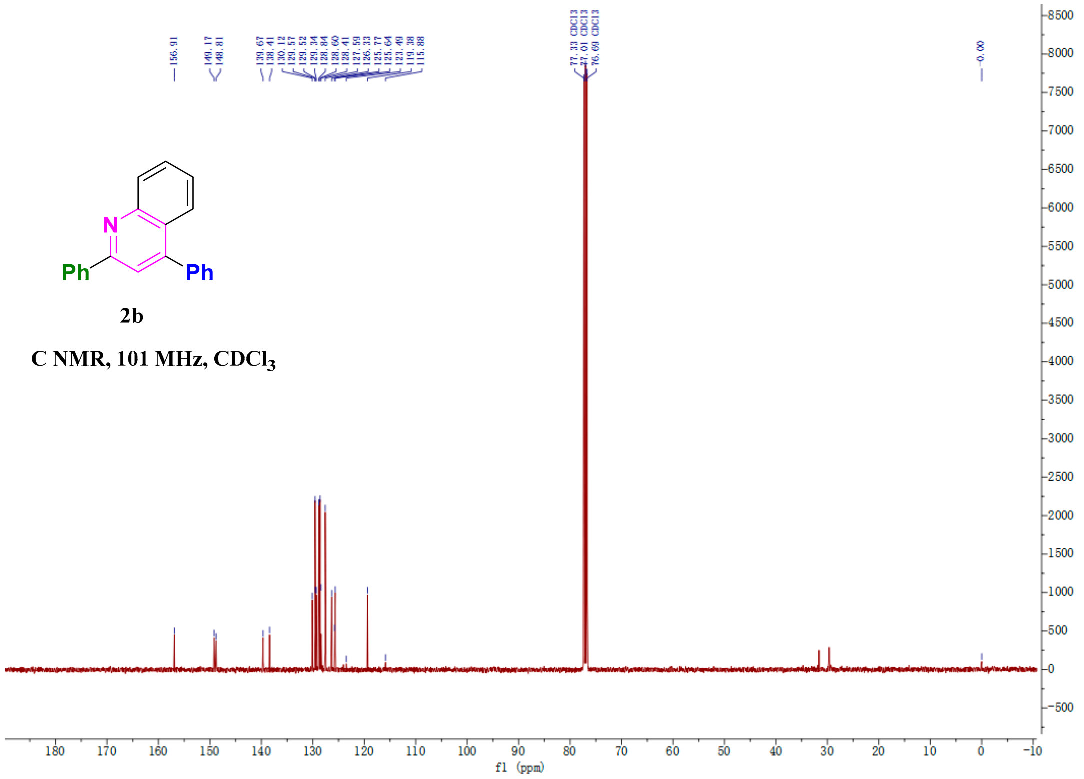
N
Ph
Ph
2b
C NMR, 101 MHz, CDCl3
f1 (ppm)

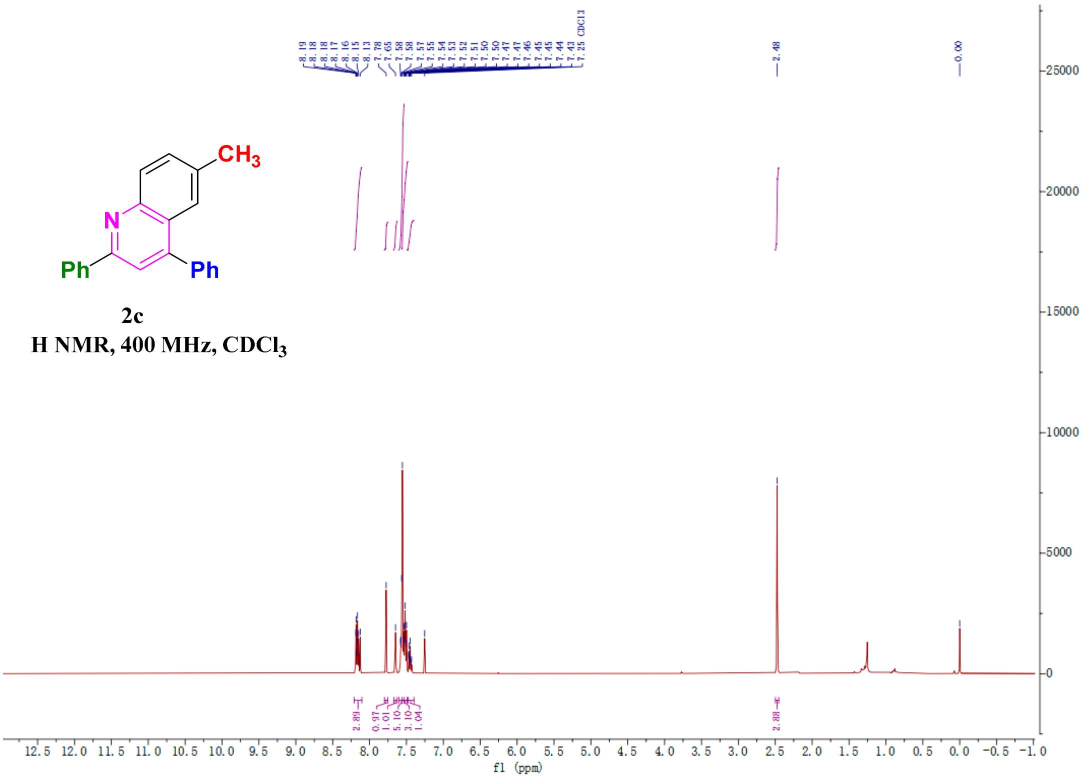
CH3
N
Ph
Ph
2c
H NMR, 400 MHz, CDCl3
f1 (ppm)

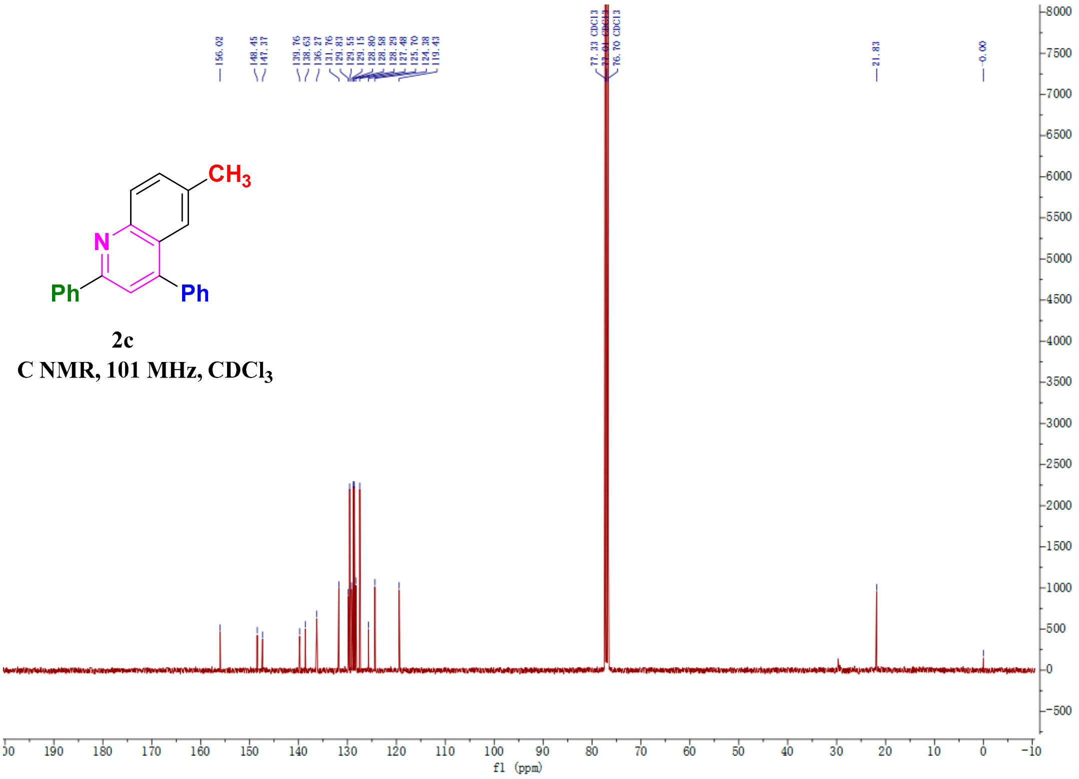
CH3
N
Ph
Ph
2c
C NMR, 101 MHz, CDCl3
f1 (ppm)

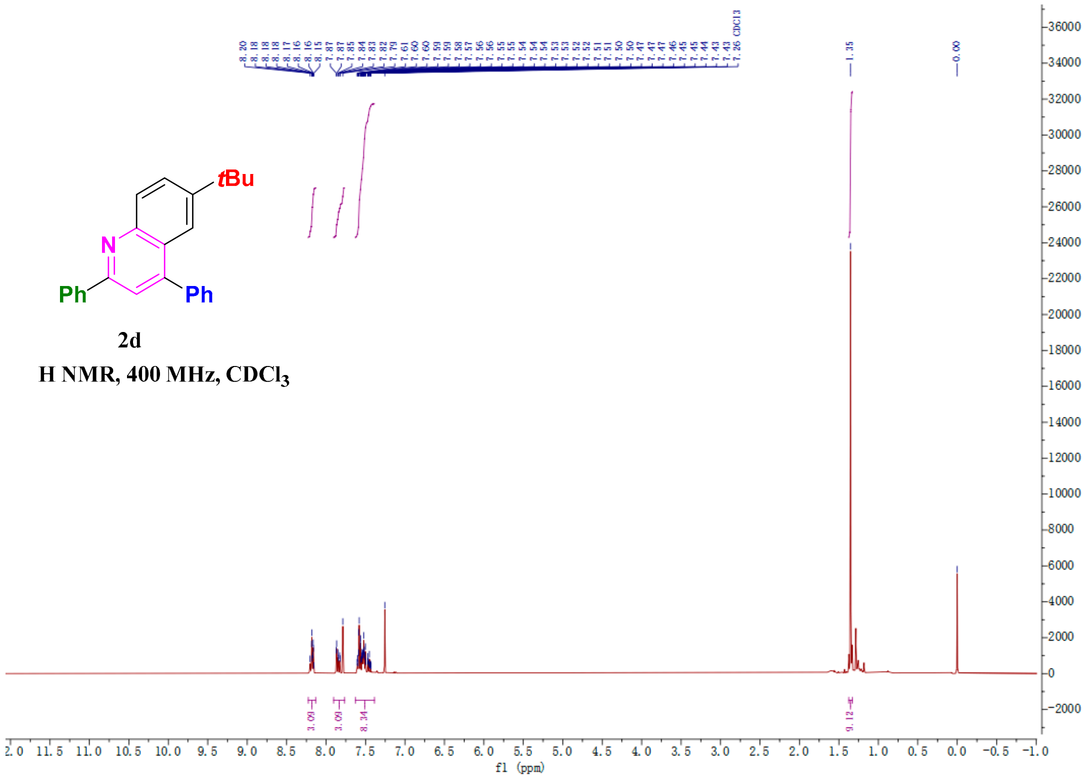

2d

H NMR, 400 MHz, $CDCl_3$

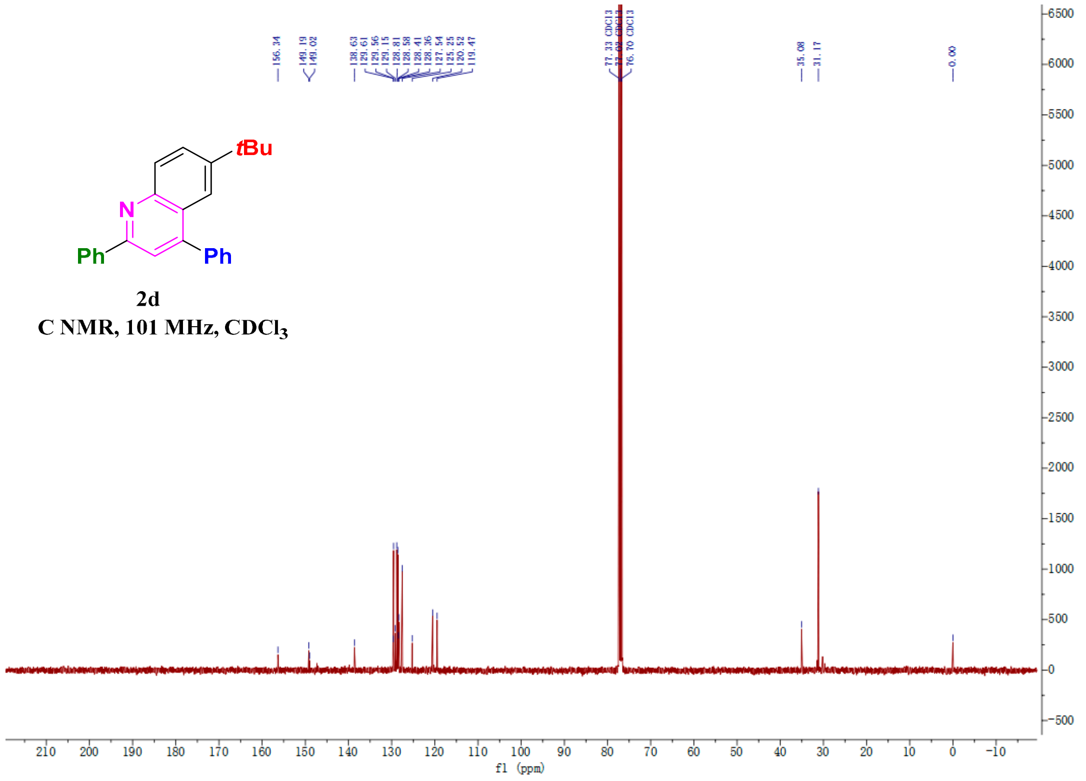

2d

C NMR, 101 MHz, $CDCl_3$

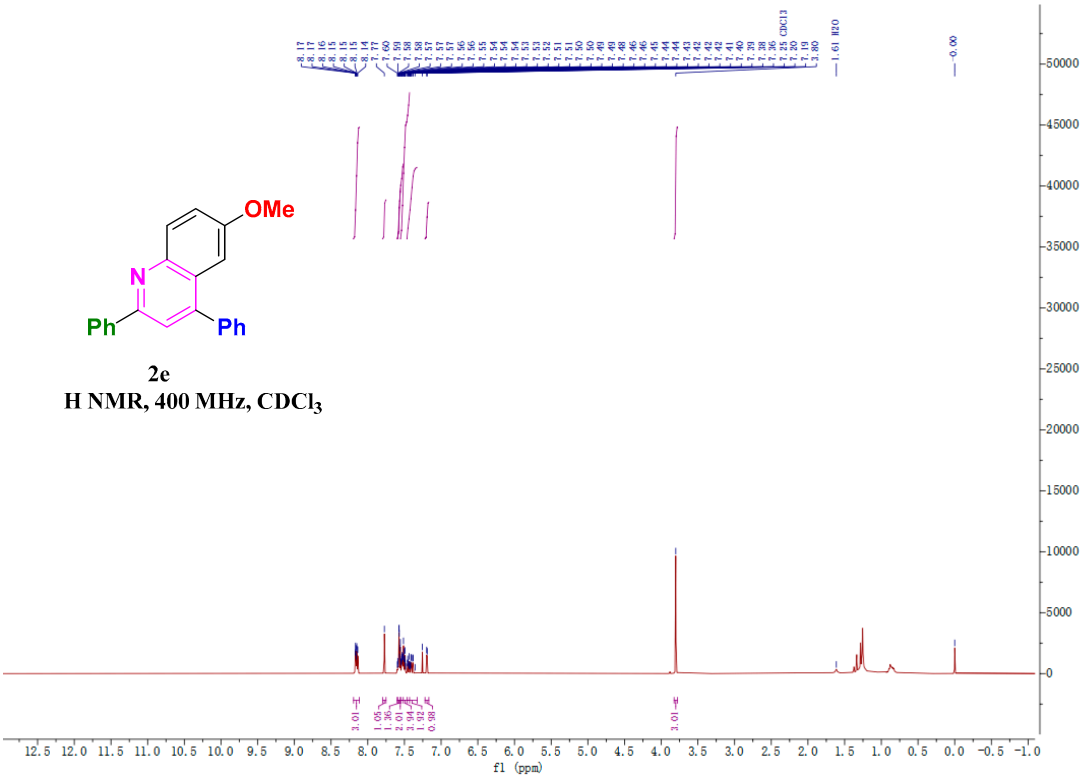

2e
H NMR, 400 MHz, $CDCl_3$

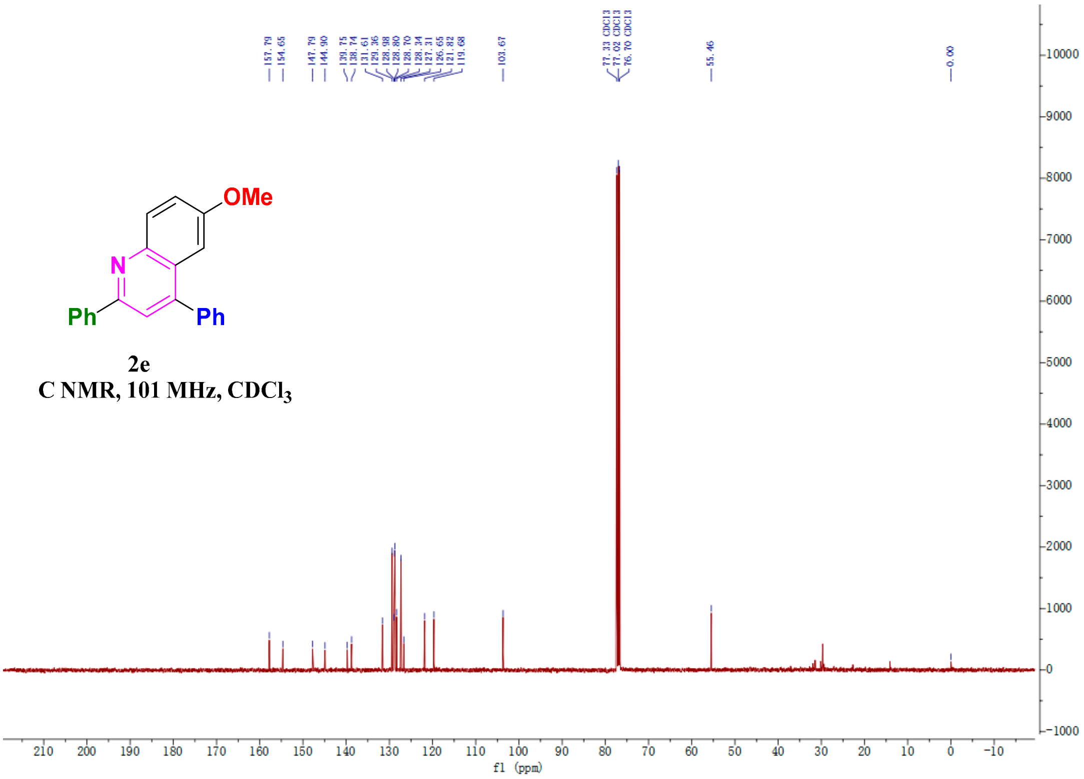

2e
C NMR, 101 MHz, $CDCl_3$

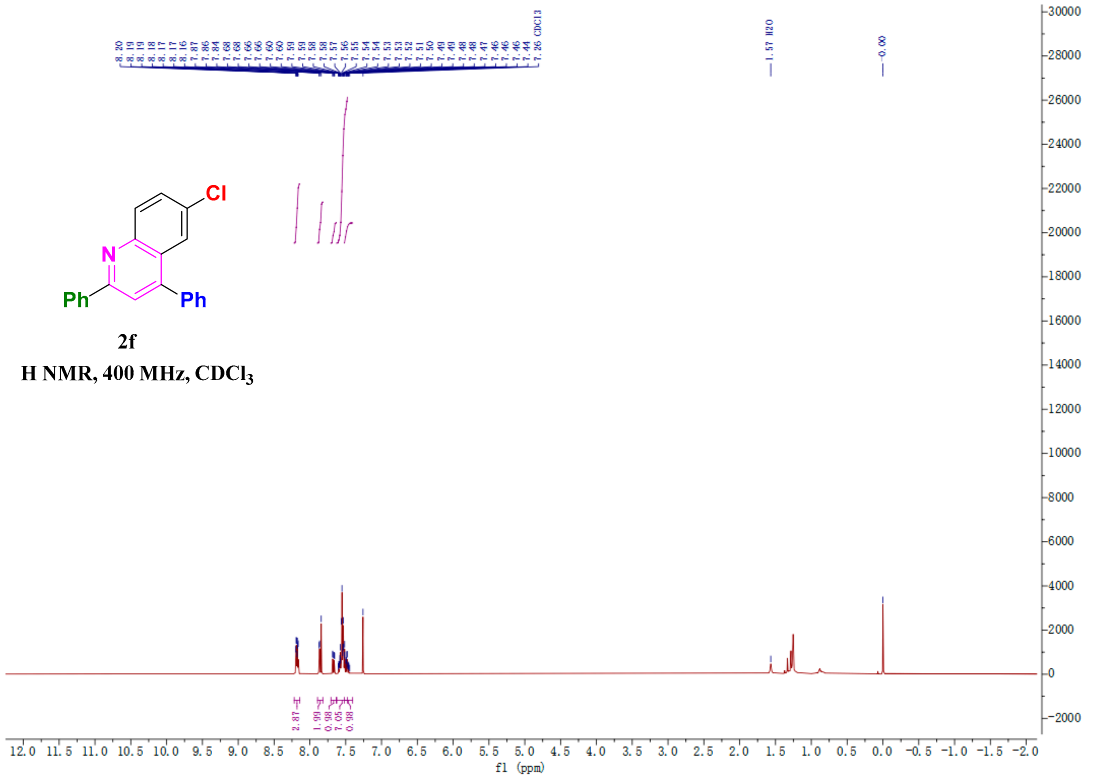

Cl
N
Ph
Ph
2f
H NMR, 400 MHz, CDCl3
7.26 CDCl3
1.57 H2O
0.00
f1 (ppm)

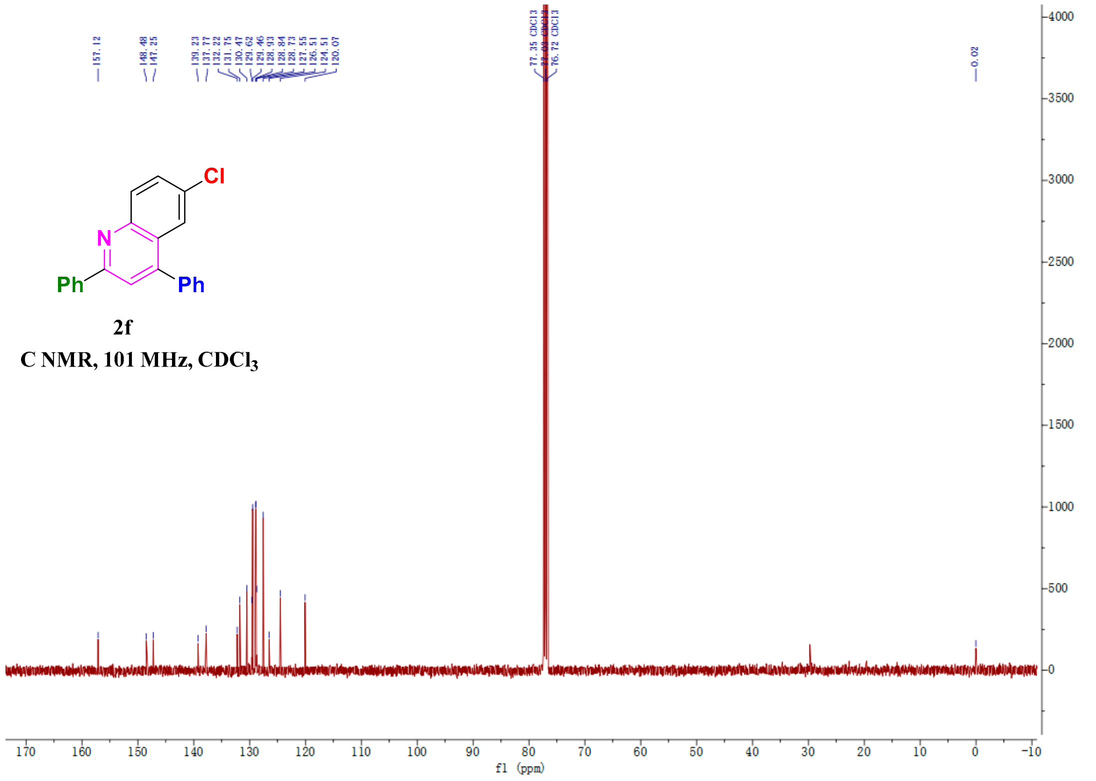

Cl
N
Ph
Ph
2f
C NMR, 101 MHz, CDCl3
157.12
148.48
147.25
139.23
137.77
132.22
131.73
130.47
129.62
129.46
128.93
128.84
128.73
127.55
126.51
124.51
120.07
77.35 CDCl3
76.72 CDCl3
0.02
f1 (ppm)

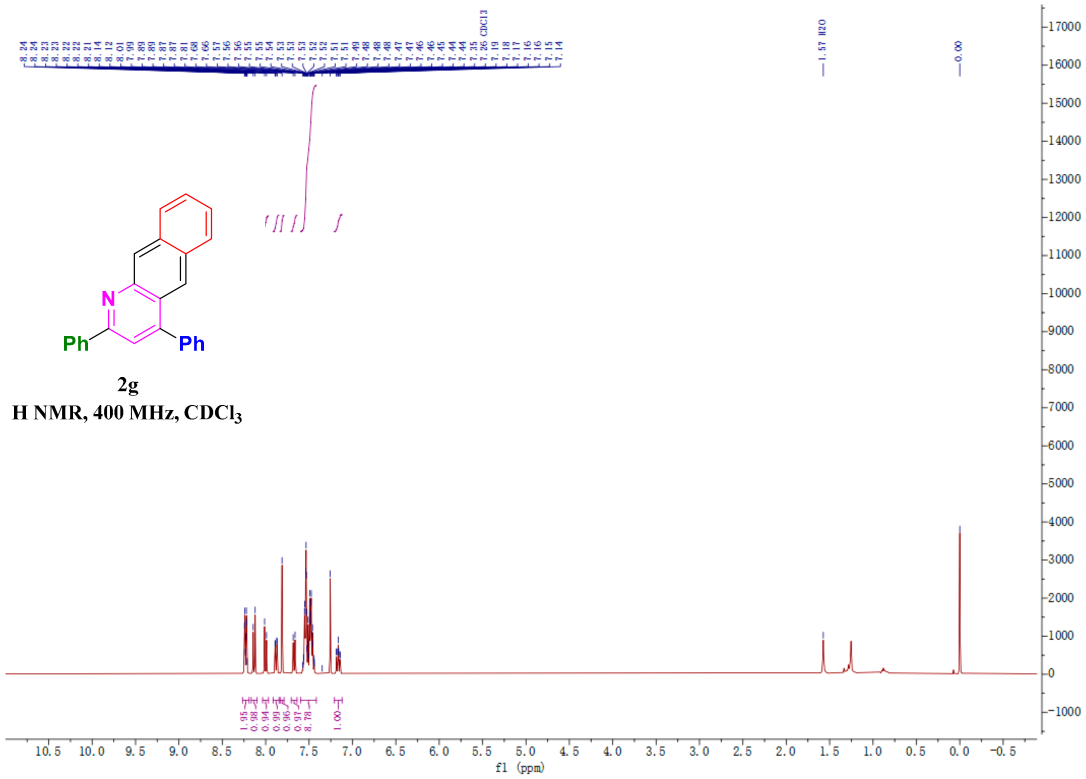
N
Ph
Ph
2g
H NMR, 400 MHz, CDCl3
1.57 H2O
0.00
7.26 CDCl3
f1 (ppm)

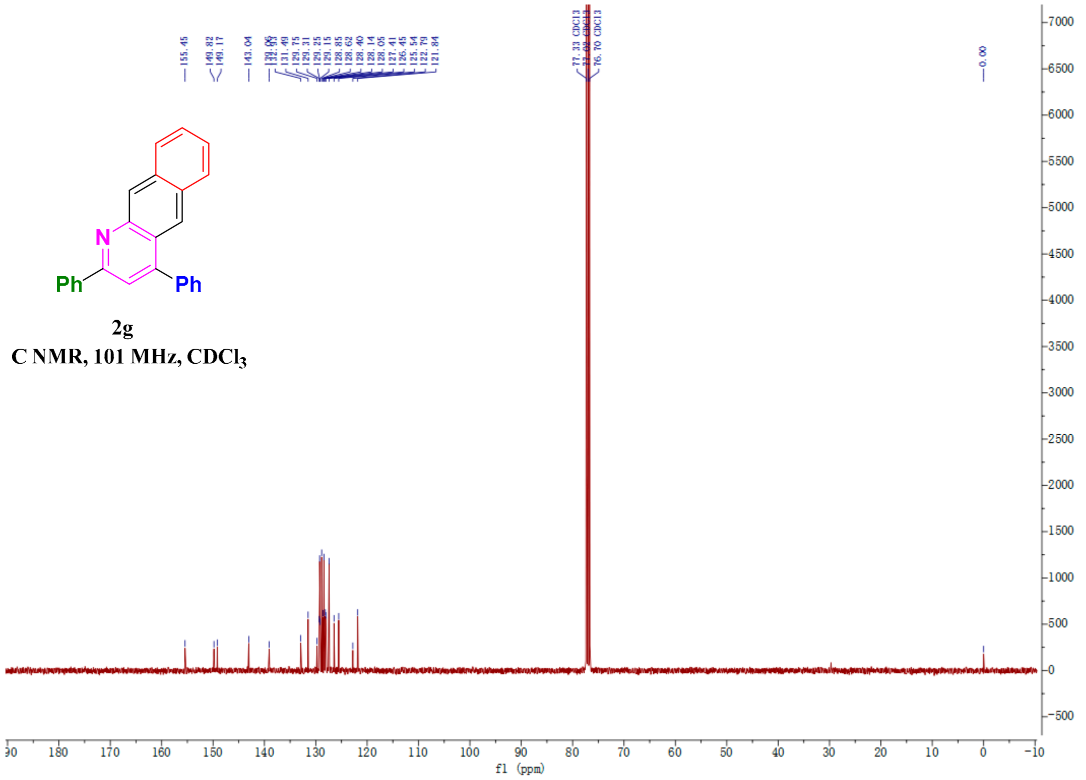
N
Ph
Ph
2g
C NMR, 101 MHz, CDCl3
155.45
149.82
149.17
143.04
77.33 CDCl3
76.70 CDCl3
0.00
f1 (ppm)

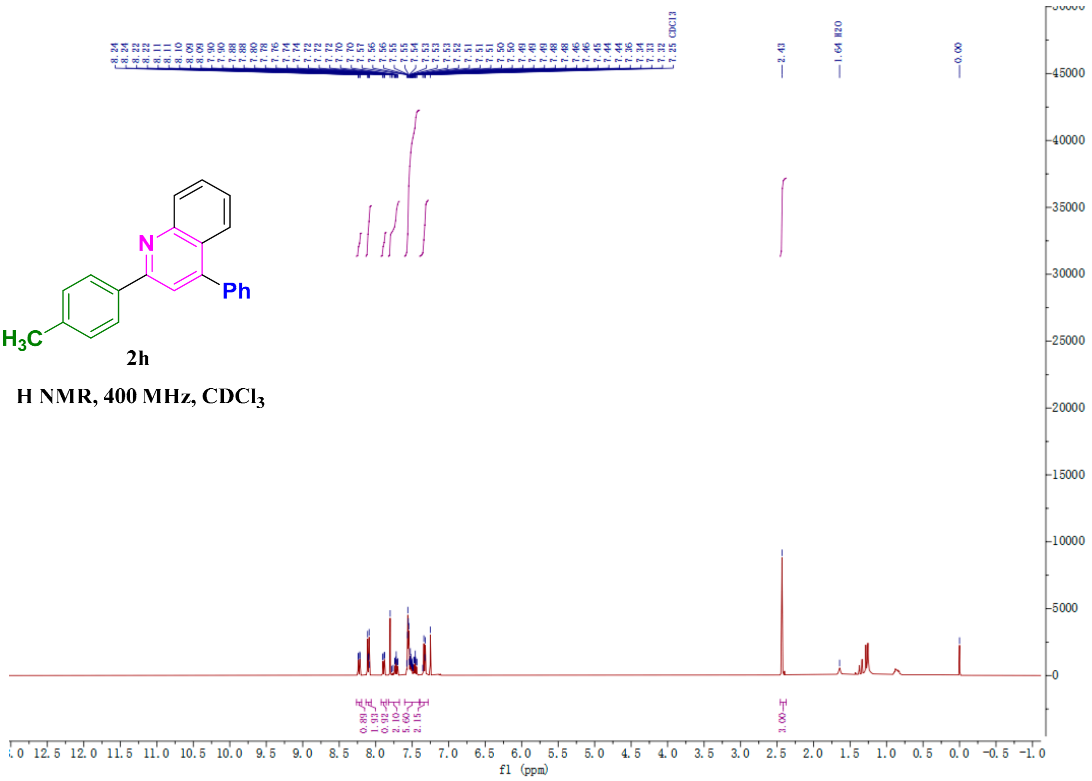

N
Ph
H_3C
2h
H NMR, 400 MHz, $CDCl_3$
2.43
1.64 H2O
0.00
7.25 CDCl3
f1 (ppm)

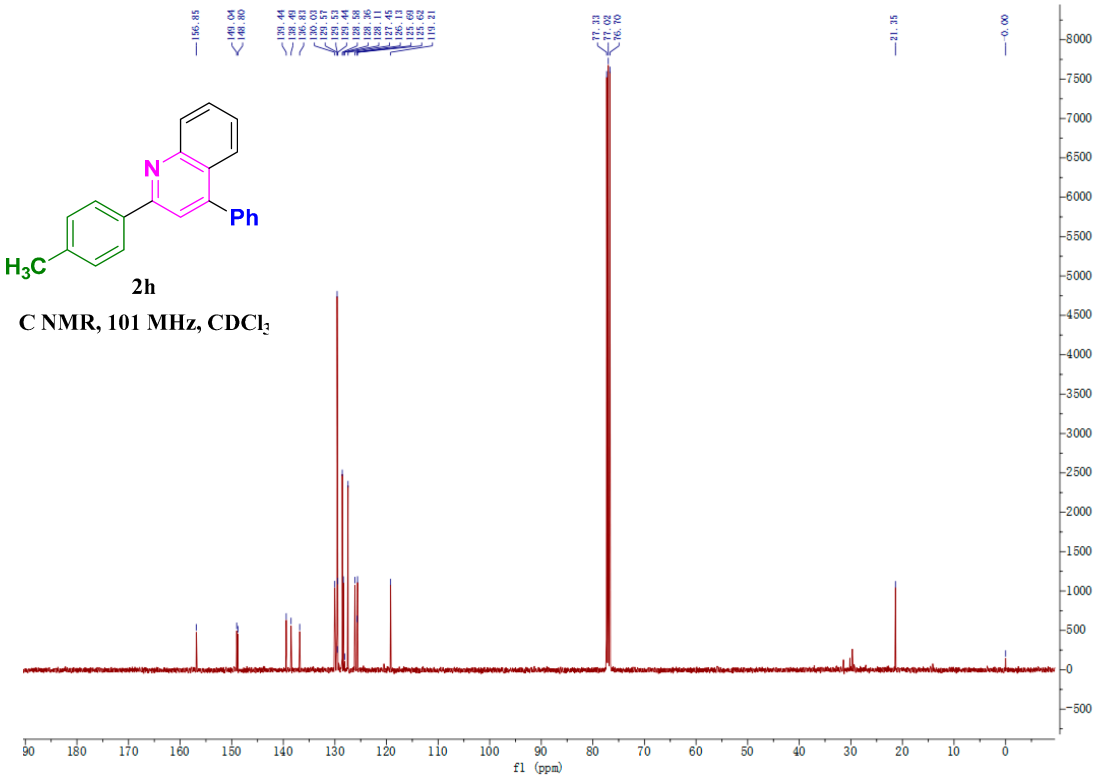

N
Ph
H_3C
2h
C NMR, 101 MHz, $CDCl_3$
156.85
149.04
148.80
139.44
138.49
136.83
130.03
129.57
129.53
129.44
128.58
128.36
128.11
127.45
126.13
125.69
125.62
119.21
77.33
77.02
76.70
21.35
0.00
f1 (ppm)

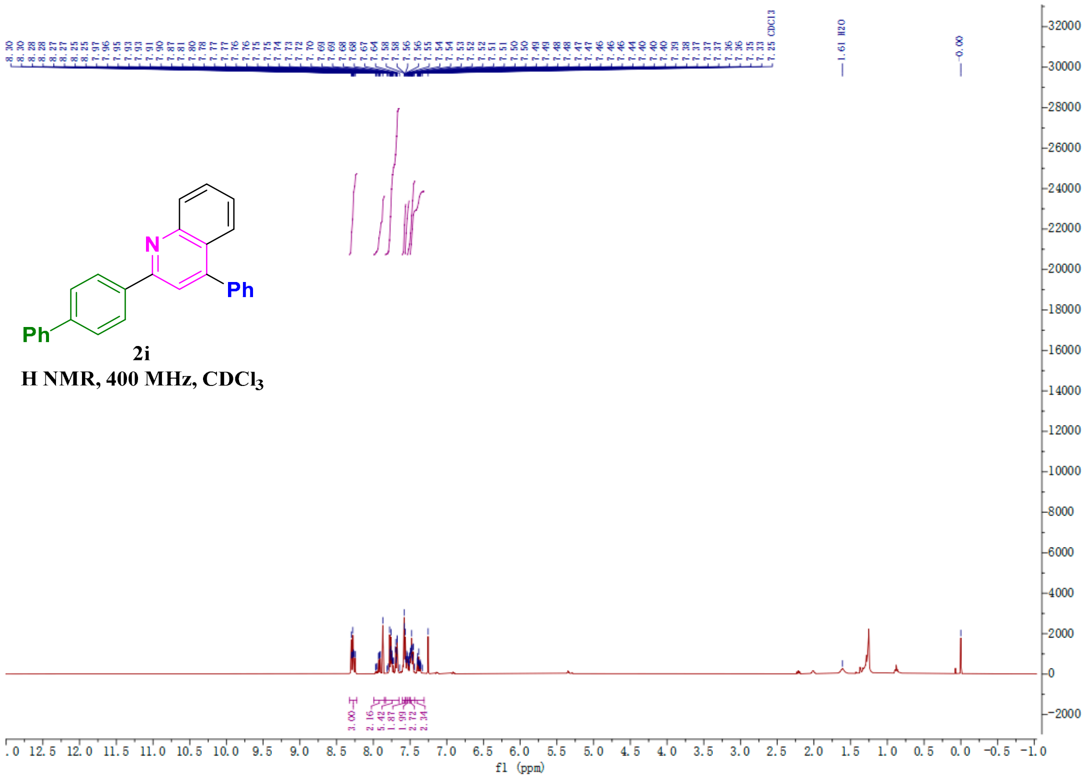
Ph
N
Ph
2i
H NMR, 400 MHz, $CDCl_3$
f1 (ppm)

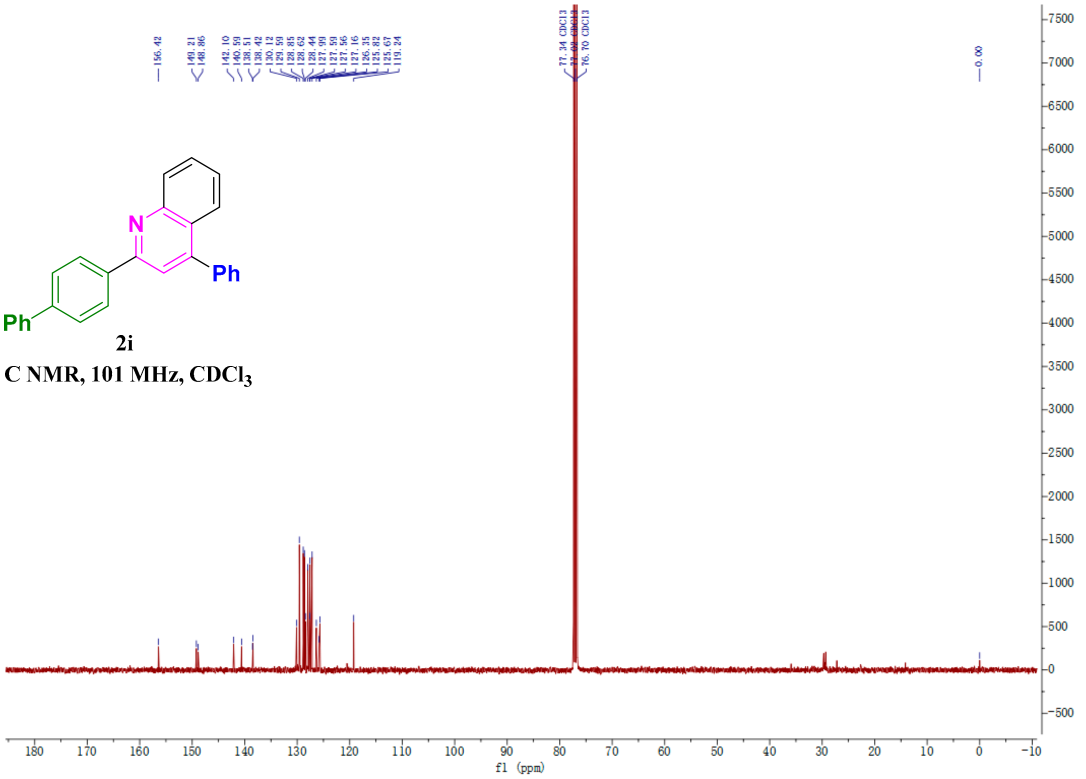
N
Ph
Ph
2i
C NMR, 101 MHz, $CDCl_3$
f1 (ppm)

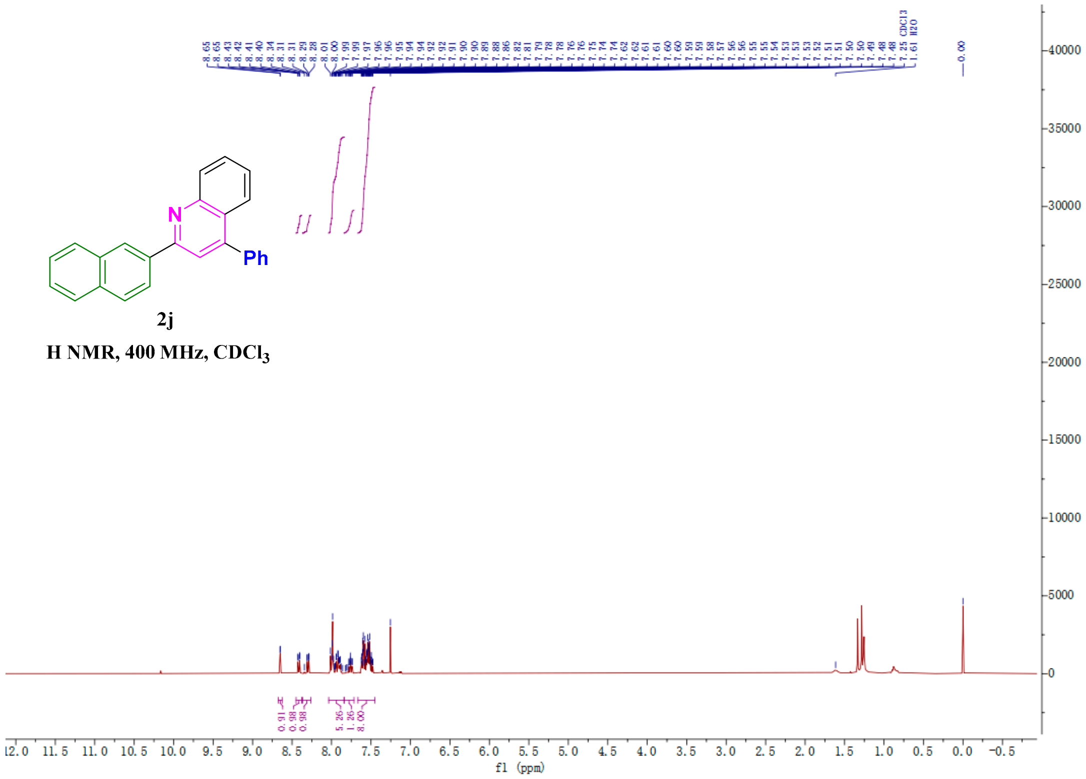
N
Ph
2j
H NMR, 400 MHz, CDCl3
f1 (ppm)

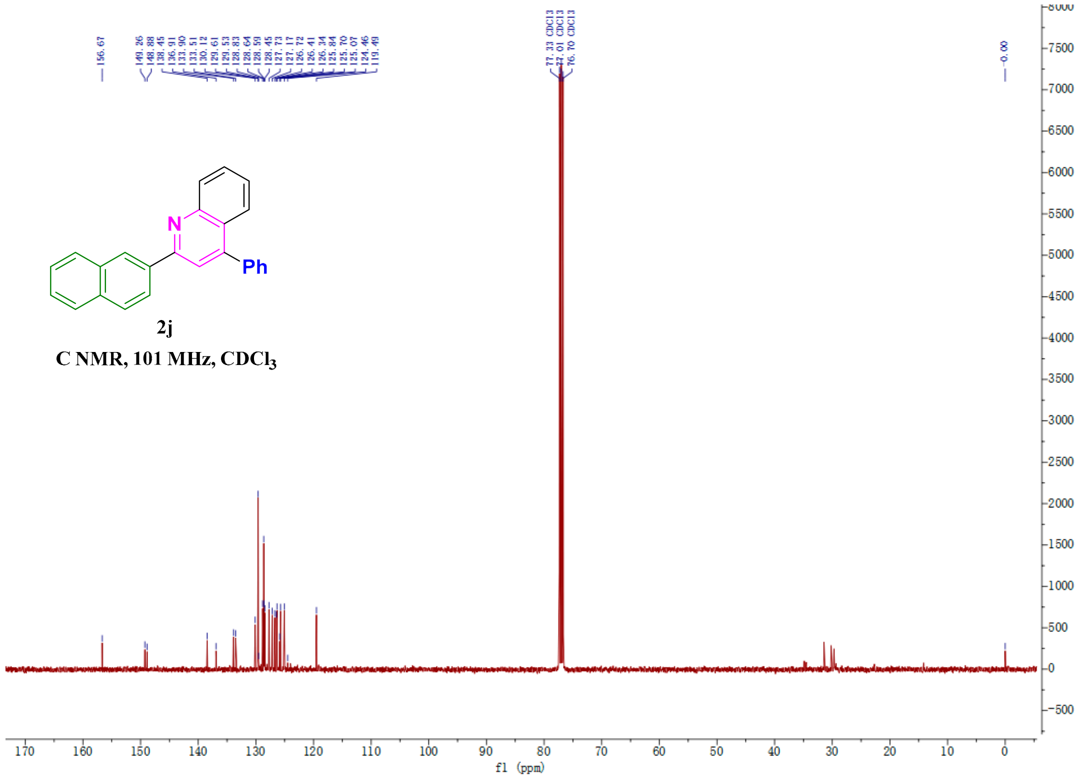
N
Ph
2j
C NMR, 101 MHz, CDCl3
f1 (ppm)

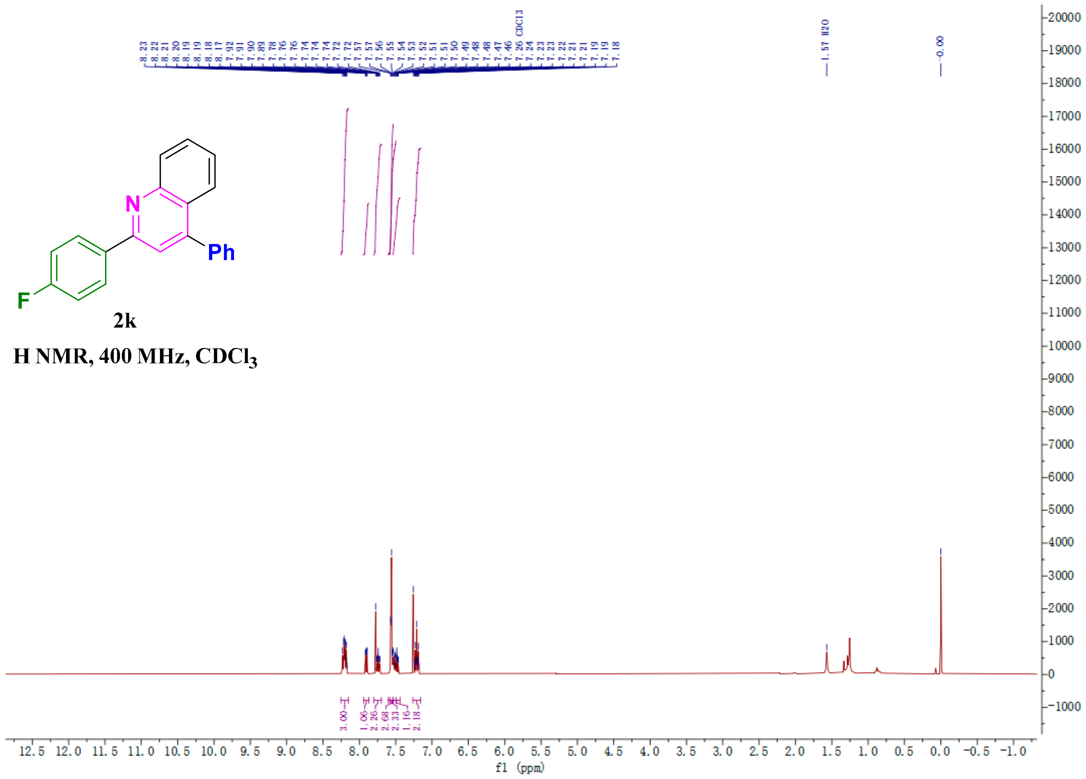

N
Ph
F
2k
H NMR, 400 MHz, CDCl3
7.26 CDCl3
1.57 H2O
0.00
3.00
1.06
2.26
2.68
2.13
1.16
2.18
f1 (ppm)

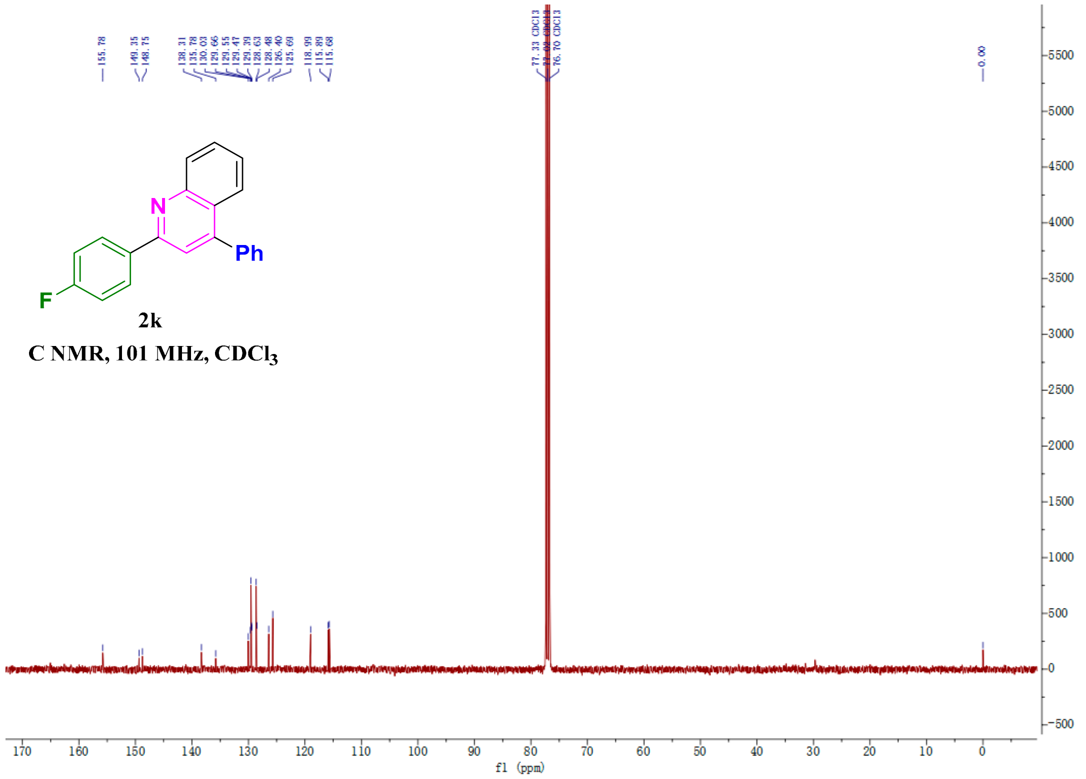

155.78
149.35
148.75
138.31
135.78
130.63
129.66
129.55
129.47
129.39
128.63
128.48
126.40
125.69
118.99
115.89
115.68
77.33 CDCl3
76.70 CDCl3
0.00
N
Ph
F
2k
C NMR, 101 MHz, CDCl3
f1 (ppm)

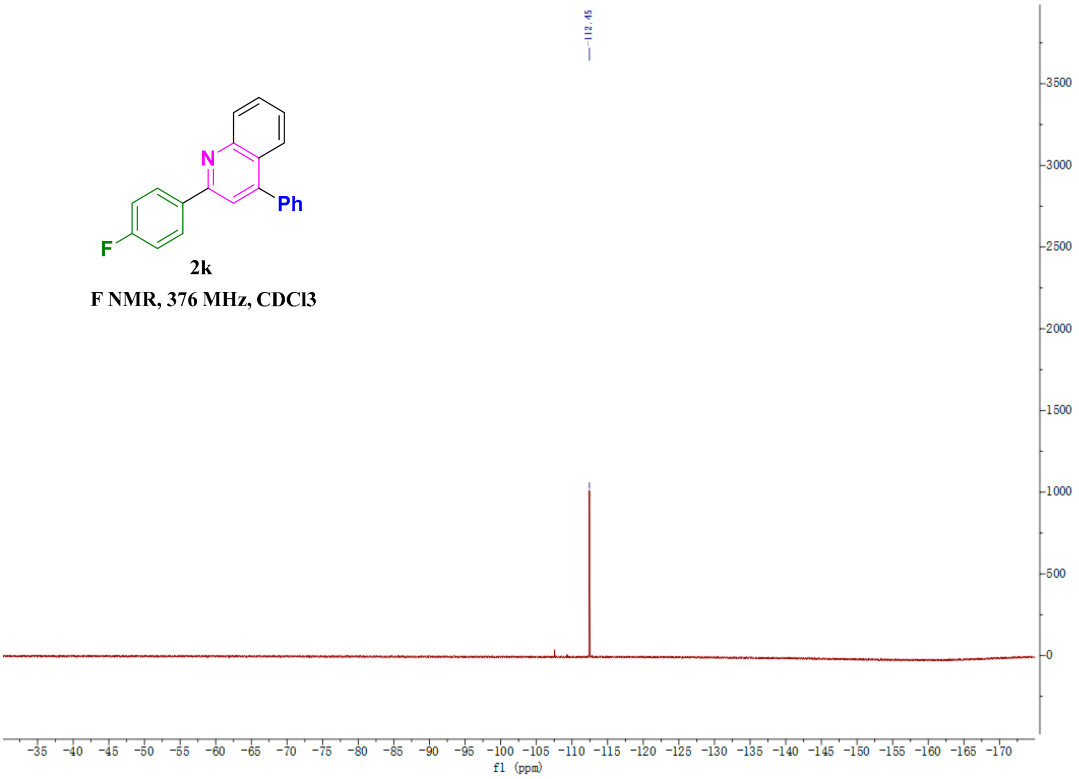

2k

F NMR, 376 MHz, CDCl3

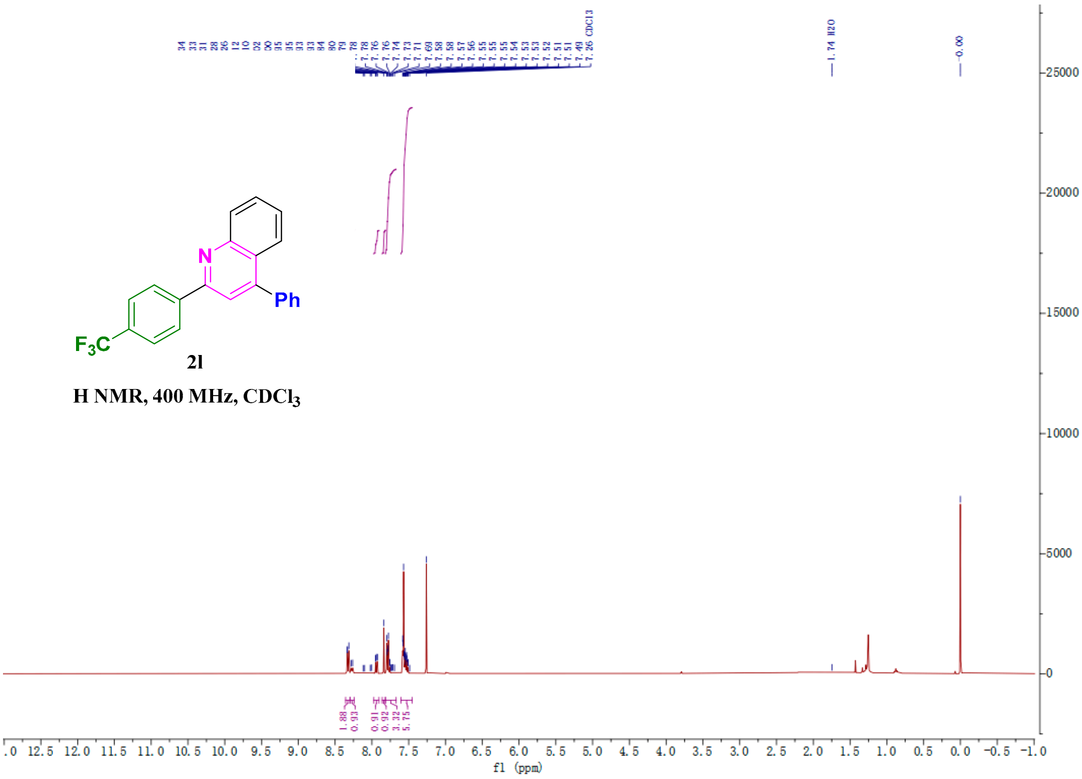

2l

H NMR, 400 MHz, $CDCl_3$

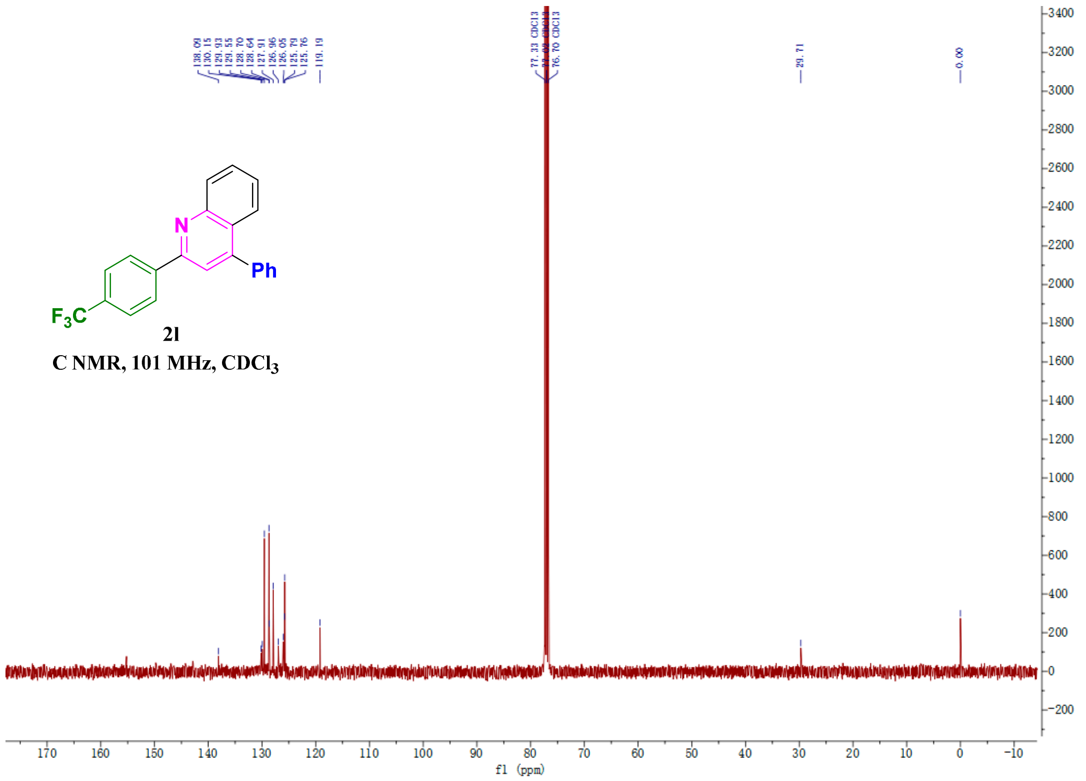

138.09
130.15
129.93
129.55
128.70
128.64
127.91
126.96
126.05
125.79
125.76
119.19
77.33 CDCl3
76.70 CDCl3
29.71
0.00
Ph
N
F3C
2l
C NMR, 101 MHz, CDCl3
f1 (ppm)

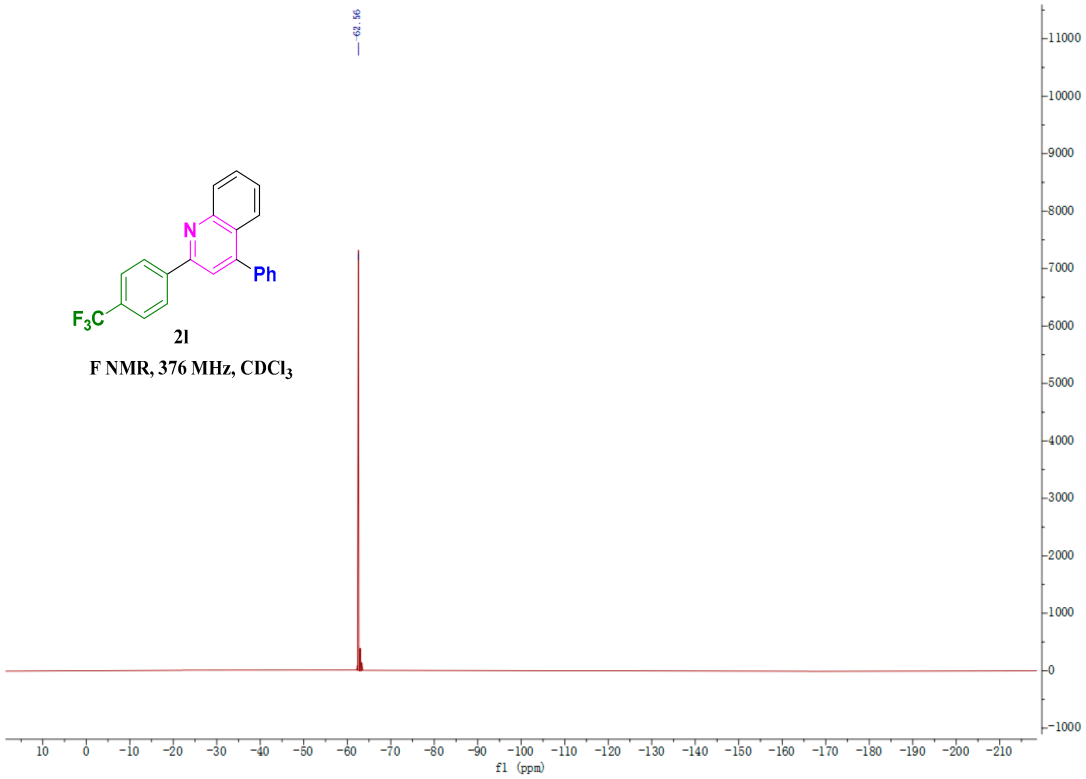

-62.56
Ph
N
F3C
2l
F NMR, 376 MHz, CDCl3
f1 (ppm)

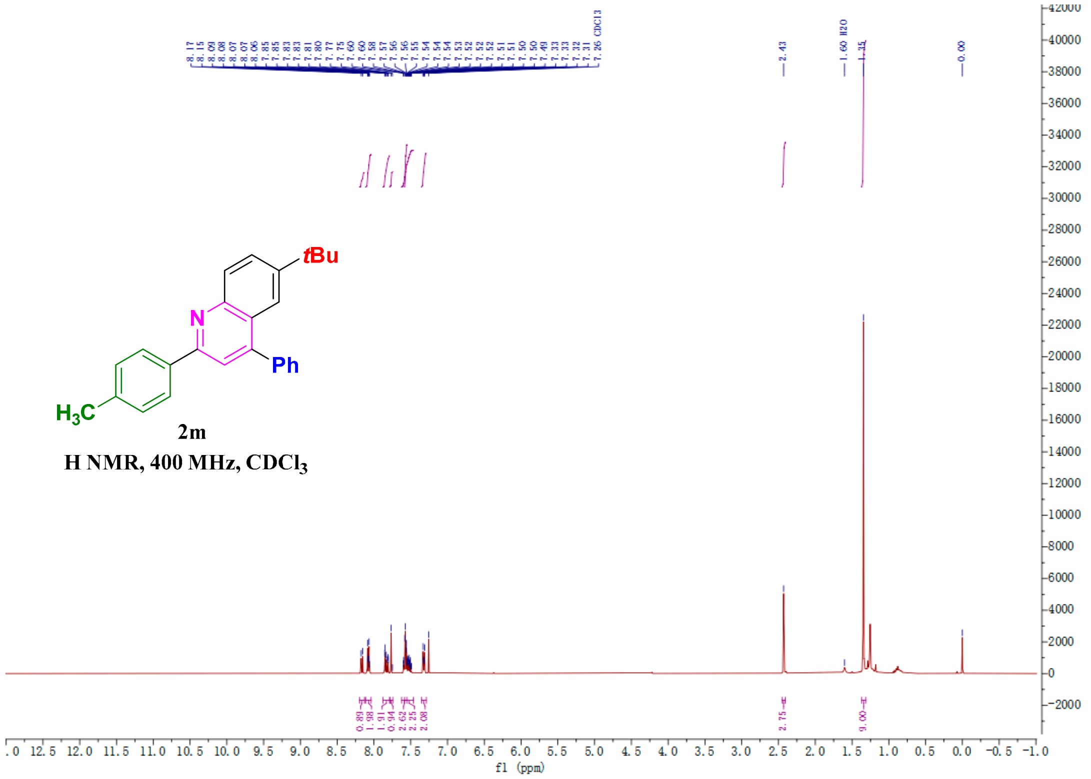
8.17 8.15 8.09 8.08 8.07 8.07 8.06 7.85 7.85 7.83 7.83 7.81 7.80 7.77 7.75 7.60 7.58 7.57 7.56 7.56 7.55 7.54 7.54 7.53 7.52 7.52 7.52 7.51 7.51 7.50 7.50 7.49 7.33 7.32 7.31 7.26 CDCl3
2.43
1.60 H2O
1.35
0.00
tBu
N
Ph
H3C
2m
H NMR, 400 MHz, CDCl3
0.89 1.98 1.91 0.94 2.62 2.25 2.08
2.75
9.00
f1 (ppm)

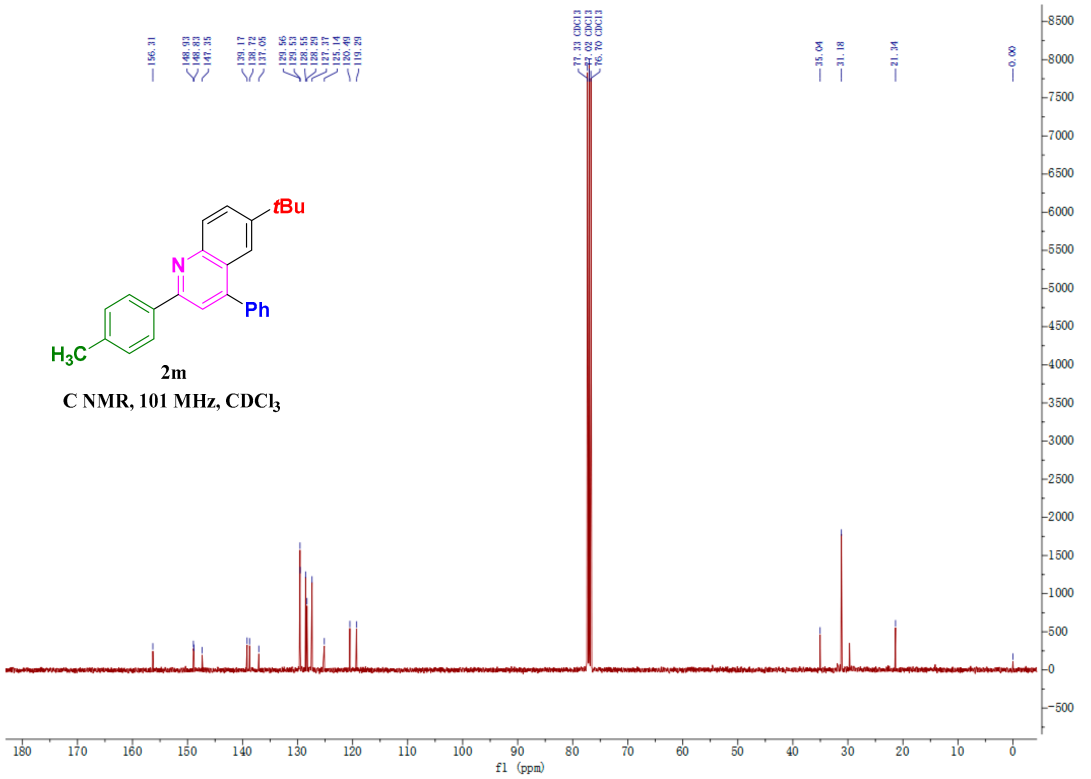
156.31
148.93 148.83 147.35
139.17 138.72 137.05
129.56 129.53 128.55 128.29 127.37 125.14 120.49 119.29
77.33 CDCl3 77.02 CDCl3 76.70 CDCl3
35.04
31.18
21.34
0.00
tBu
N
Ph
H3C
2m
C NMR, 101 MHz, CDCl3
f1 (ppm)

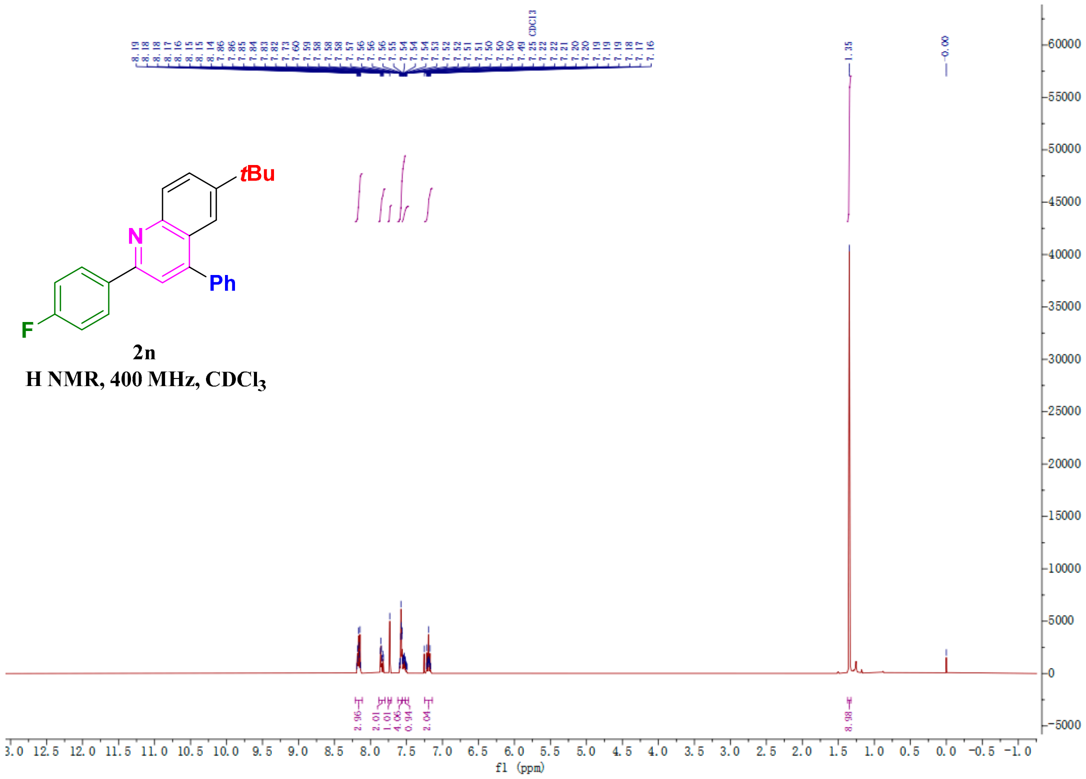
tBu
N
Ph
F
2n
H NMR, 400 MHz, CDCl3
1.35
0.00
2.96
2.01
1.01
1.01
4.06
0.94
2.04
8.98
f1 (ppm)

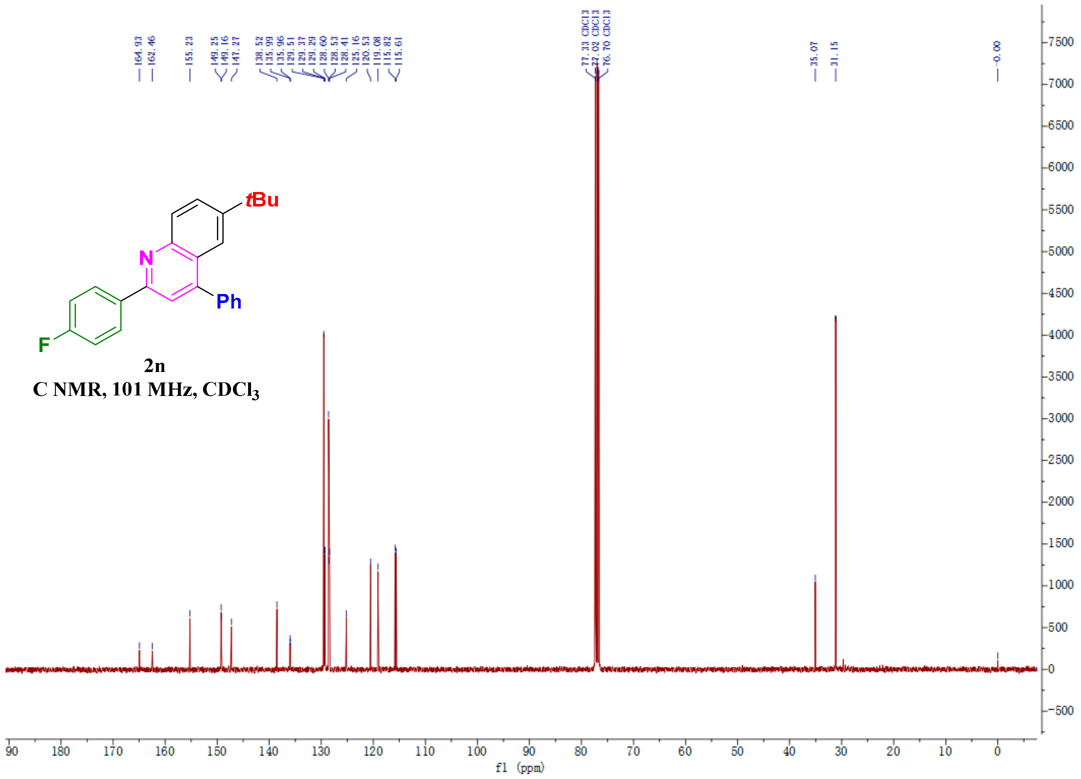
164.93
162.46
155.23
149.25
149.16
147.27
138.52
135.99
135.96
129.51
129.37
129.29
128.60
128.53
128.41
125.16
120.53
119.08
115.82
115.61
77.33 CDCl3
77.02 CDCl3
76.70 CDCl3
35.07
31.15
0.00
tBu
N
Ph
F
2n
C NMR, 101 MHz, CDCl3
f1 (ppm)

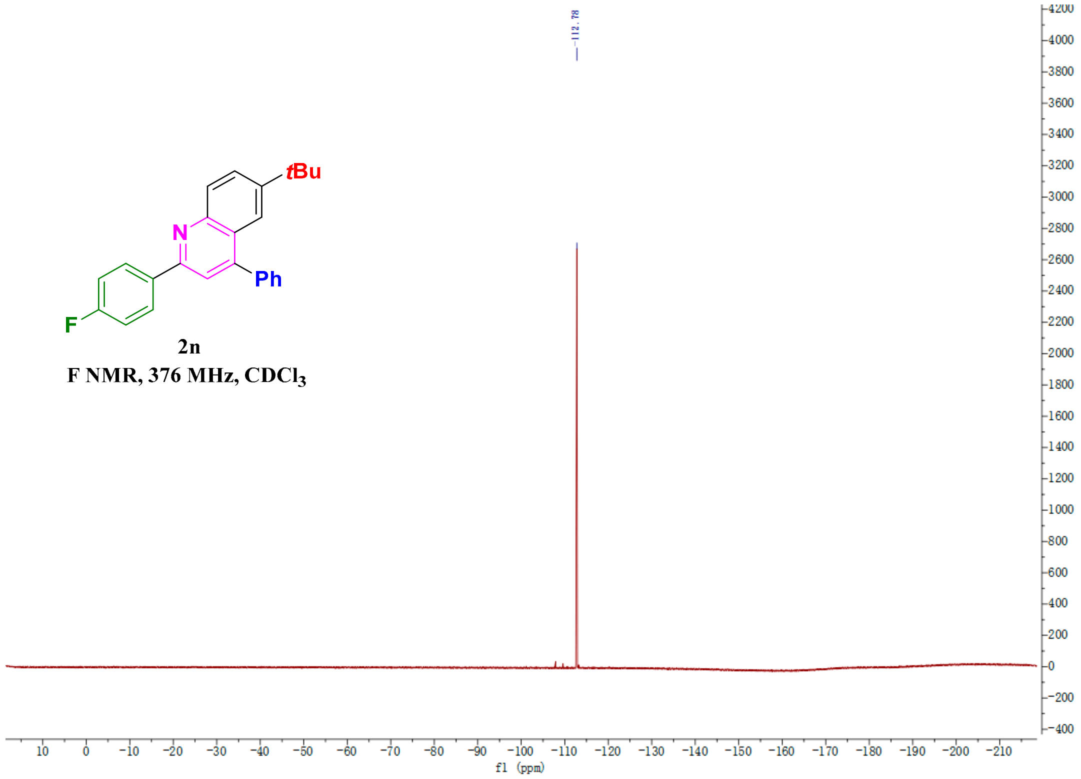

2n
F NMR, 376 MHz, $CDCl_3$

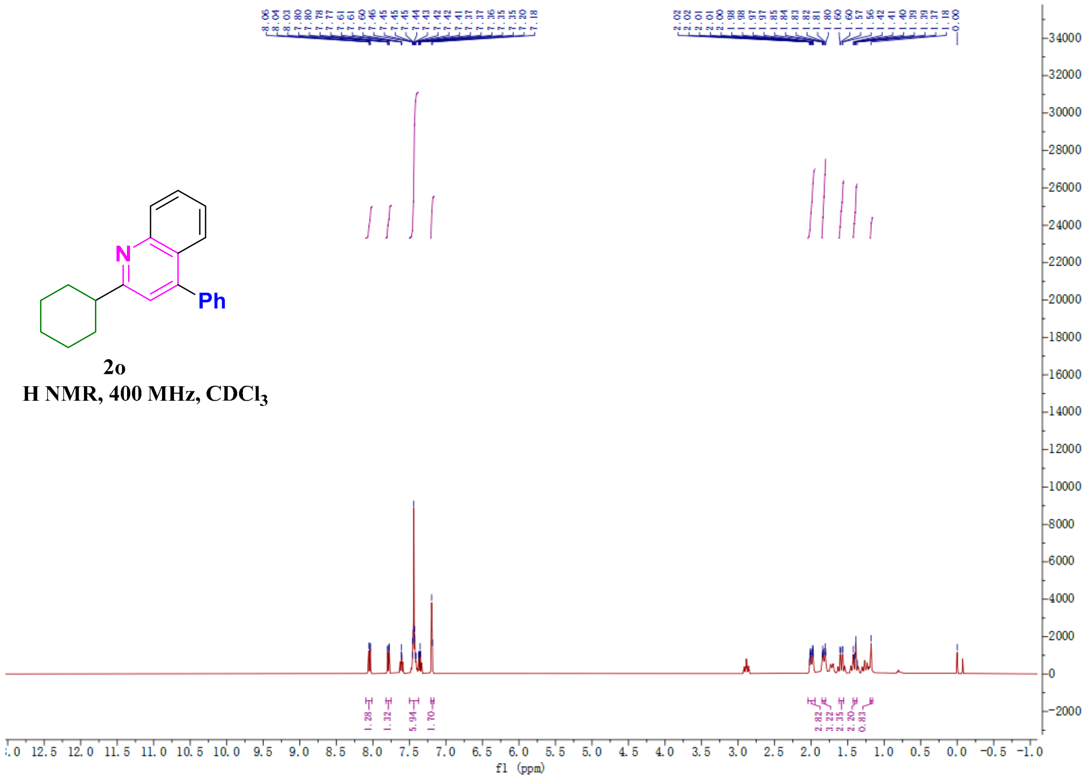

2o
H NMR, 400 MHz, $CDCl_3$

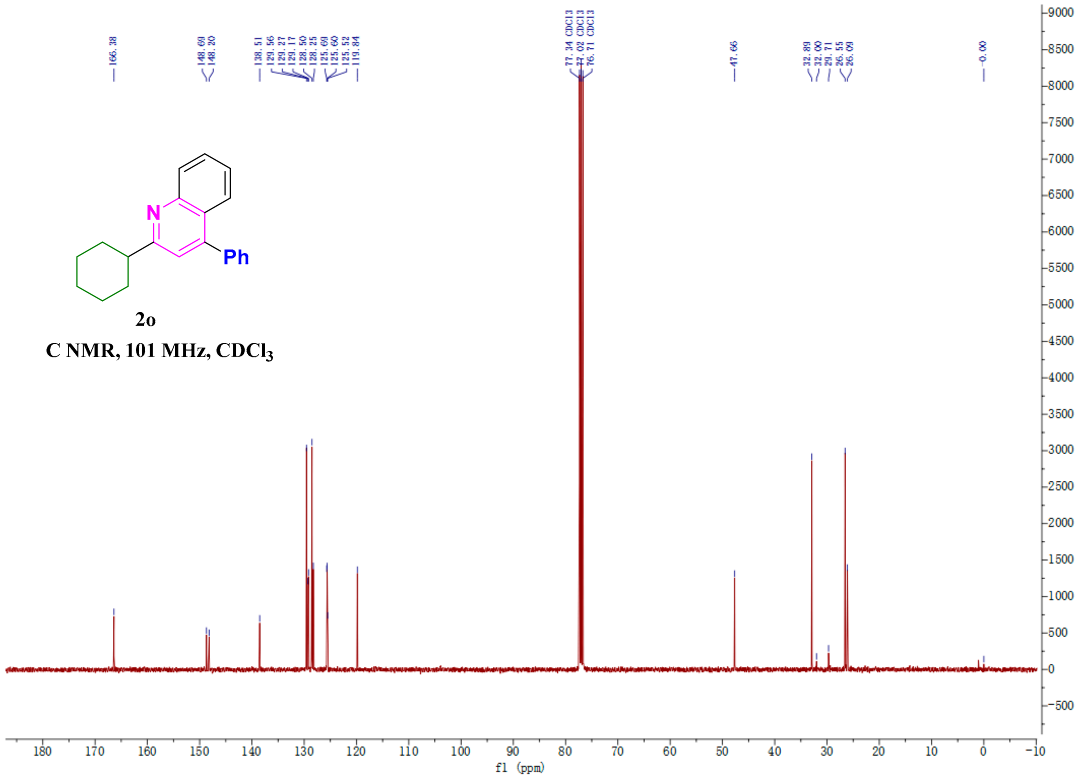

2o

C NMR, 101 MHz, $CDCl_3$

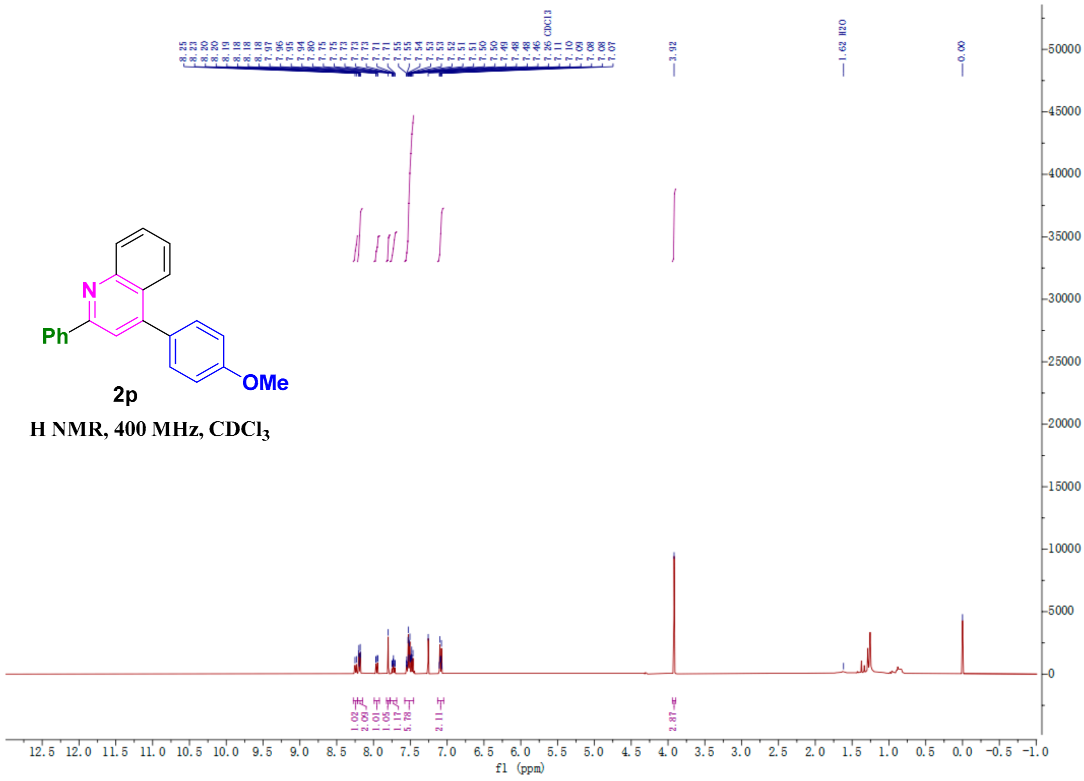

2p

H NMR, 400 MHz, $CDCl_3$

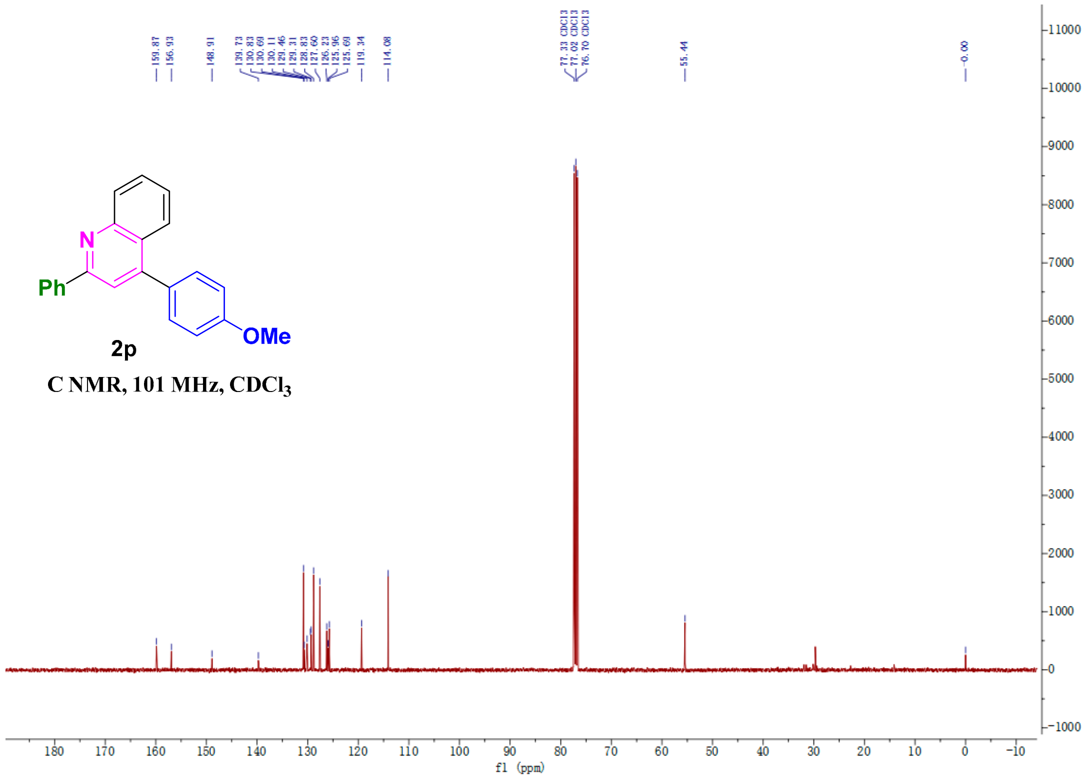

2p
C NMR, 101 MHz, CDCl3
159.87
156.93
148.91
139.73
130.83
130.69
130.11
129.46
129.31
128.83
127.60
126.23
125.96
125.69
119.34
114.08
77.33 CDCl3
77.02 CDCl3
76.70 CDCl3
55.44
0.00
f1 (ppm)

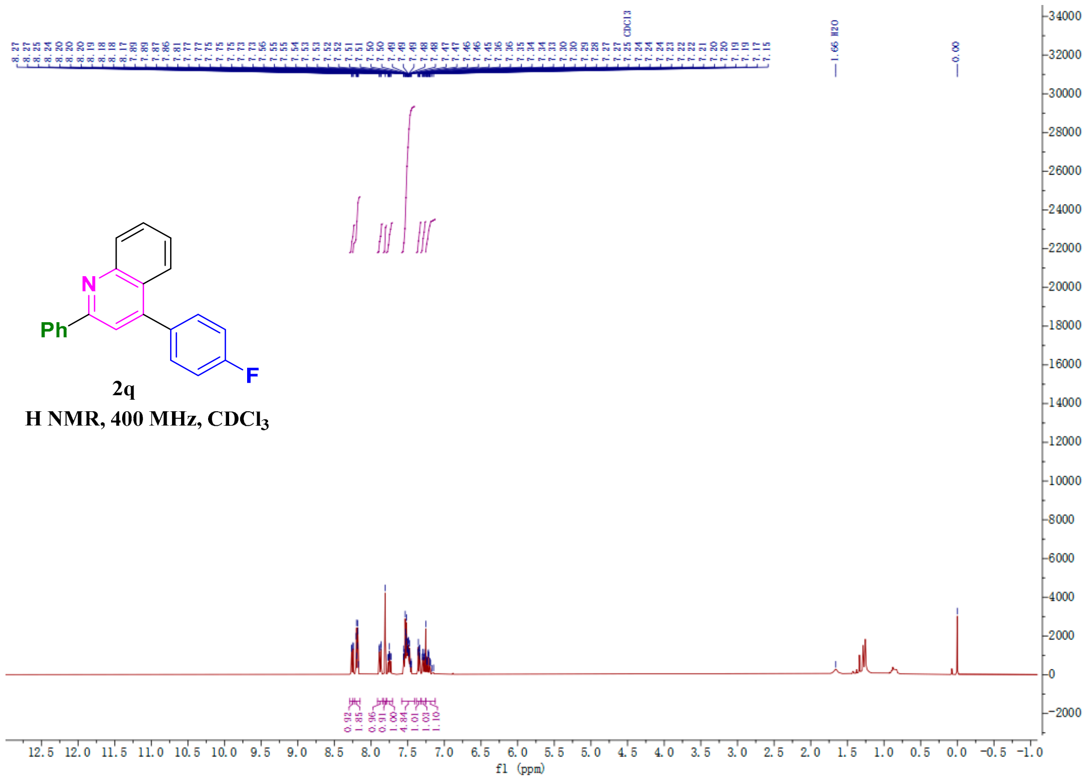

2q
H NMR, 400 MHz, CDCl3
1.66 H2O
0.00
CDCl3
f1 (ppm)

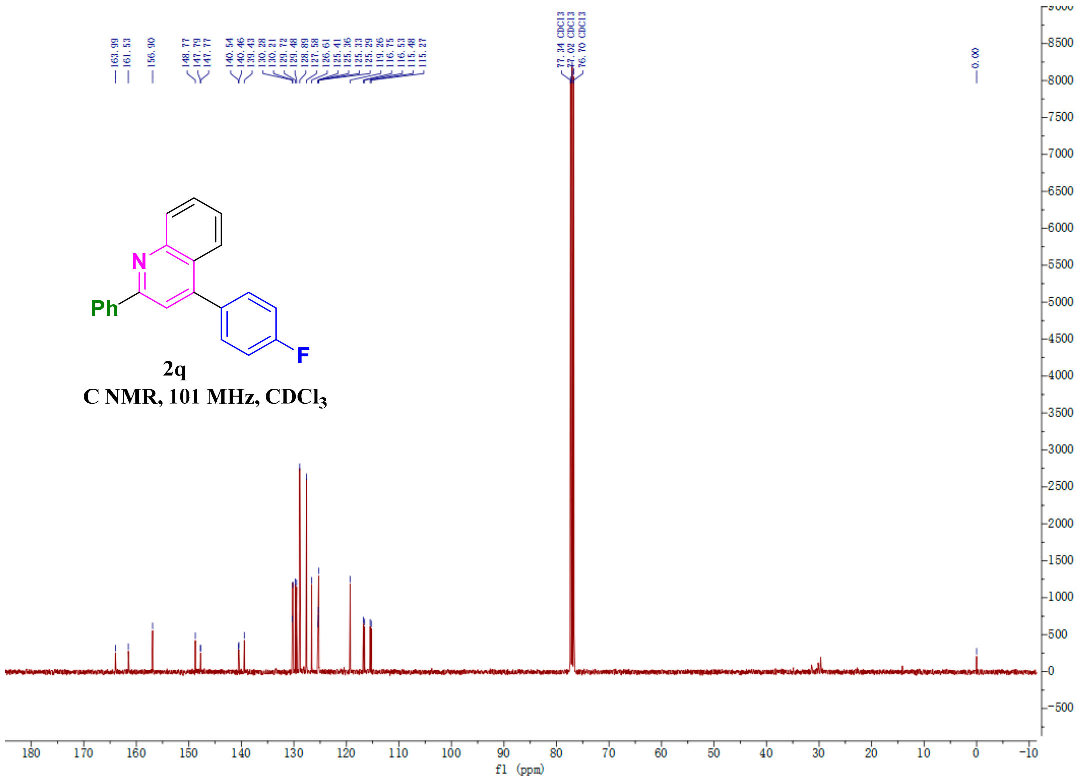
163.99
161.53
156.90
148.77
147.79
147.77
140.54
140.46
139.43
130.28
130.21
129.72
129.48
128.89
127.58
126.61
125.41
125.36
125.33
125.29
119.26
116.75
116.53
115.48
115.27
77.34 CDCl3
77.02 CDCl3
76.70 CDCl3
0.00
N
Ph
F
2q
C NMR, 101 MHz, CDCl3
f1 (ppm)

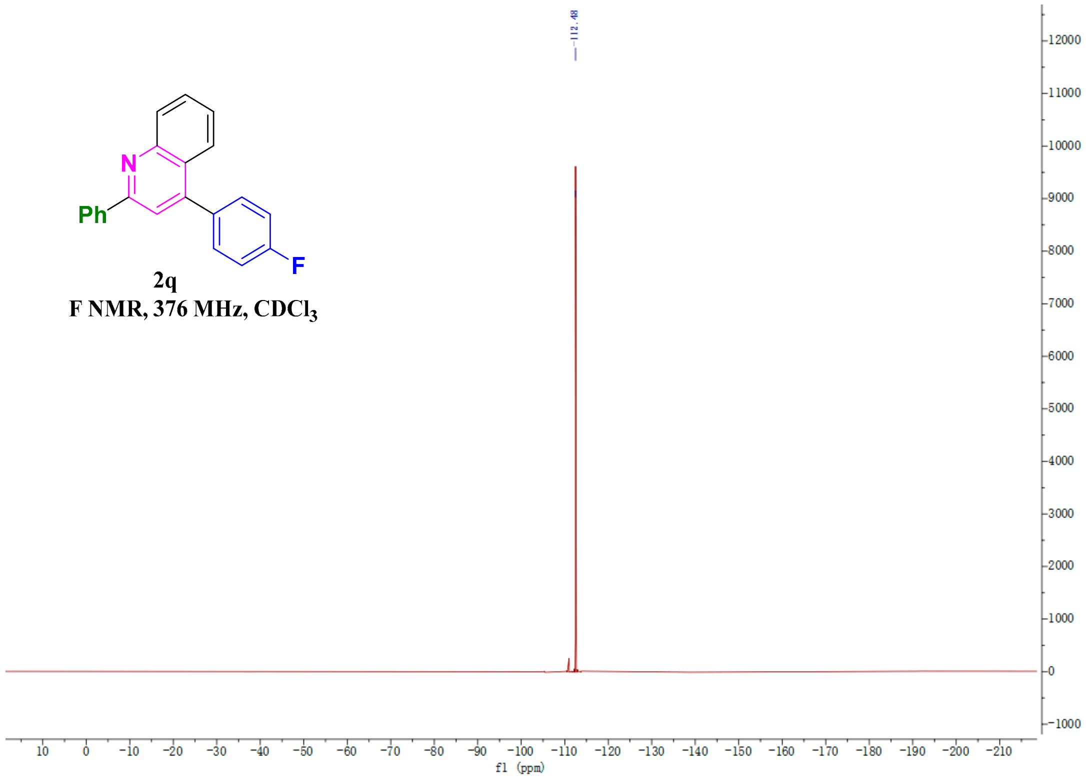
-112.48
N
Ph
F
2q
F NMR, 376 MHz, CDCl3
f1 (ppm)

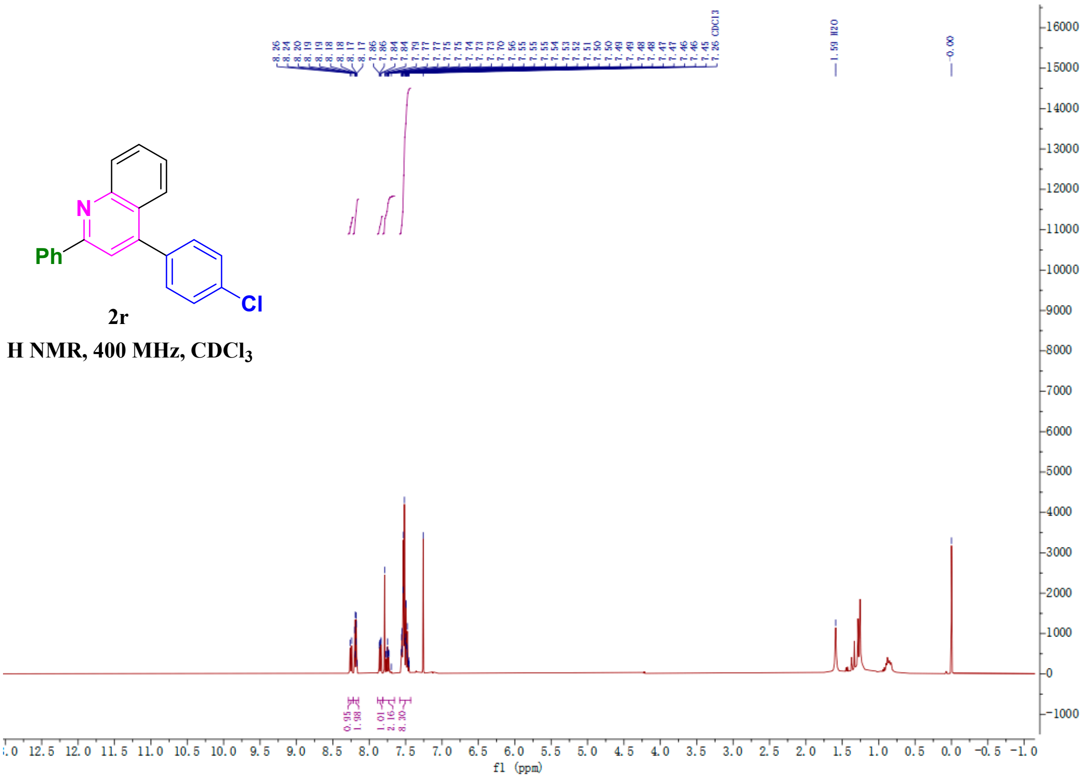

2r

H NMR, 400 MHz, $CDCl_3$

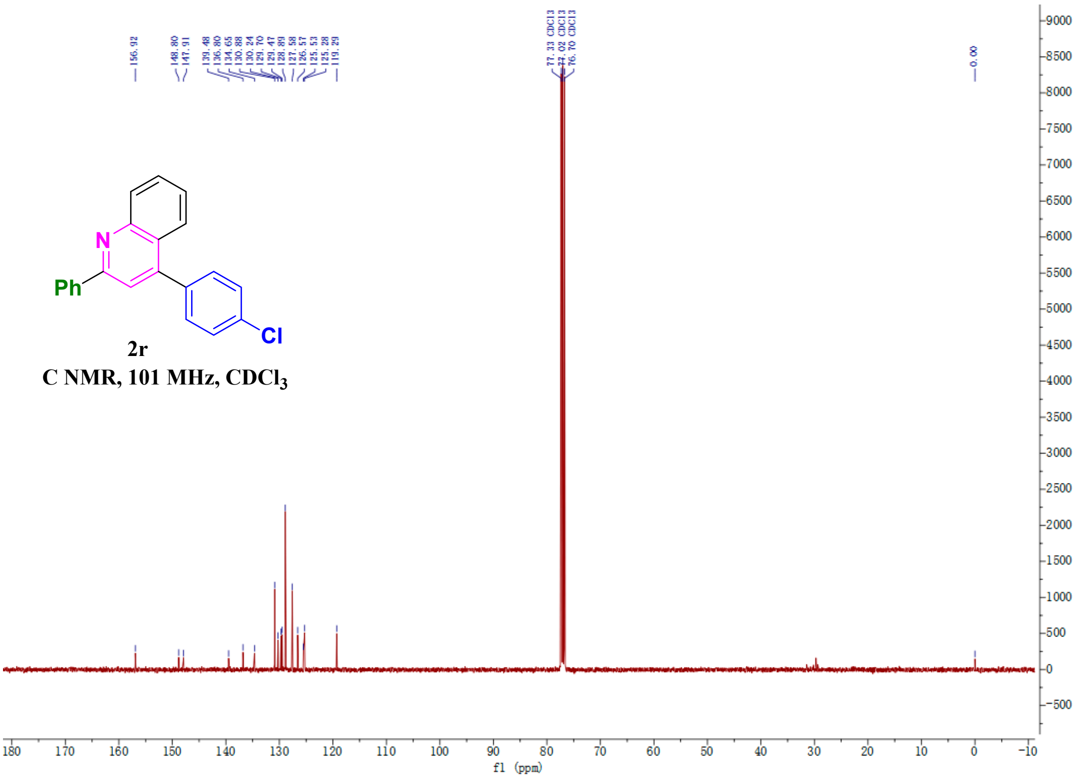

2r

C NMR, 101 MHz, $CDCl_3$

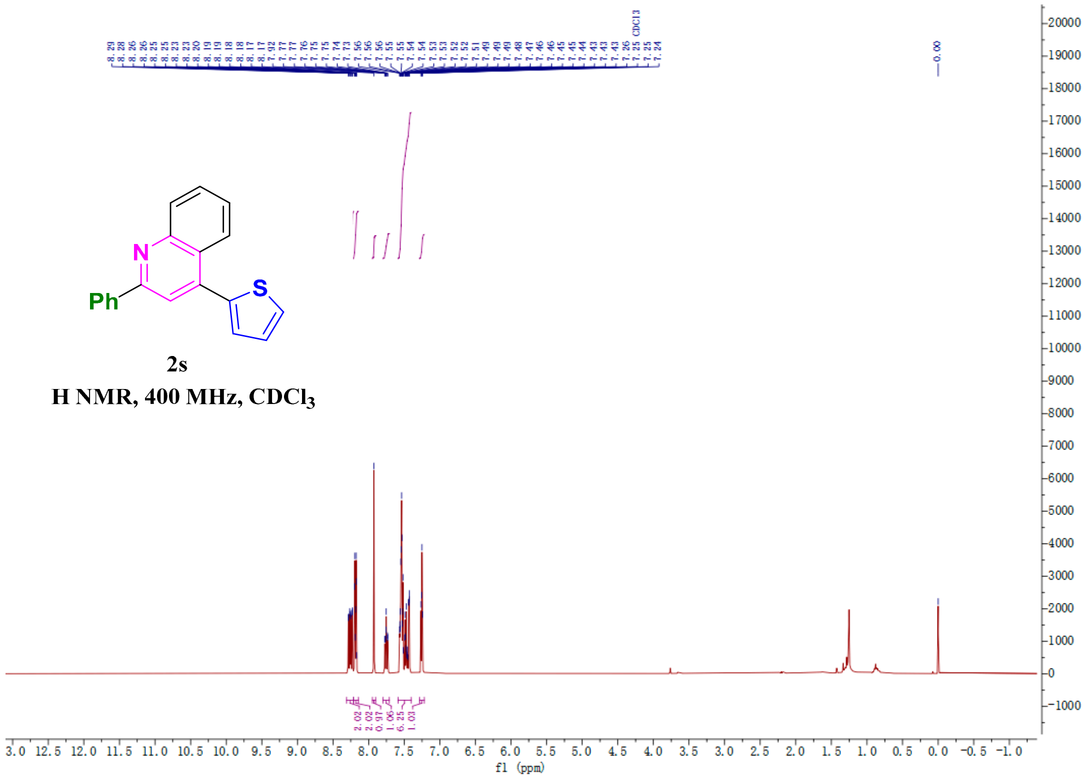

2s

H NMR, 400 MHz, CDCl$_3$

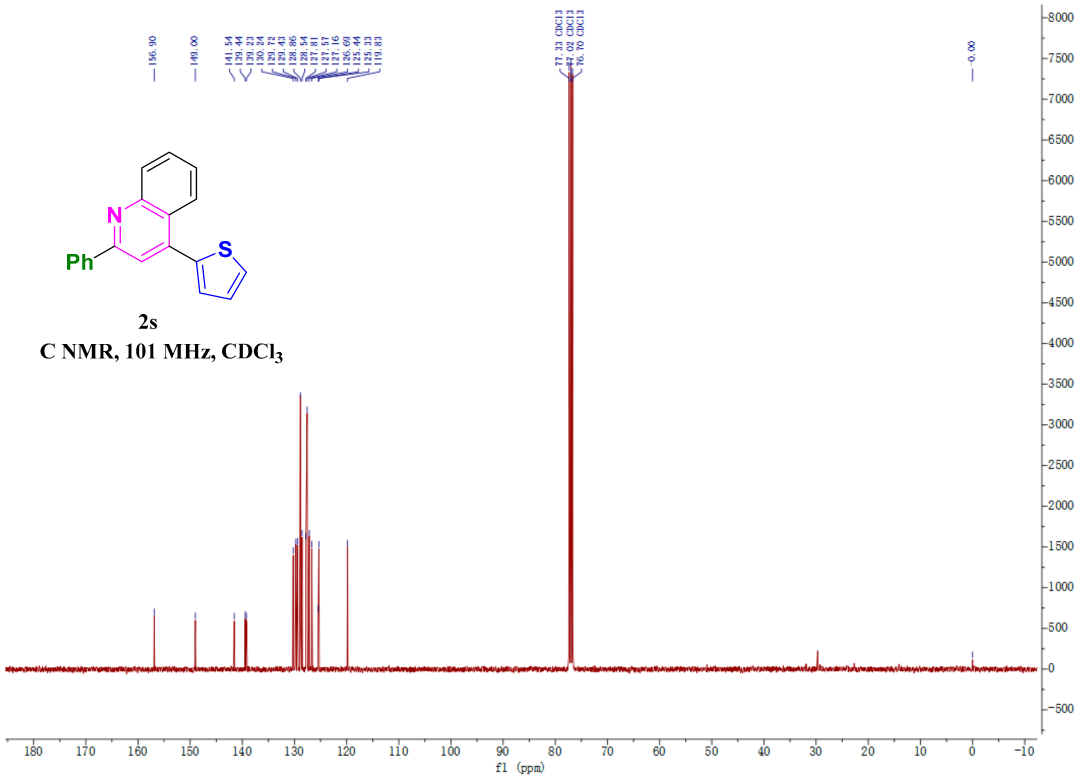

2s

C NMR, 101 MHz, CDCl$_3$

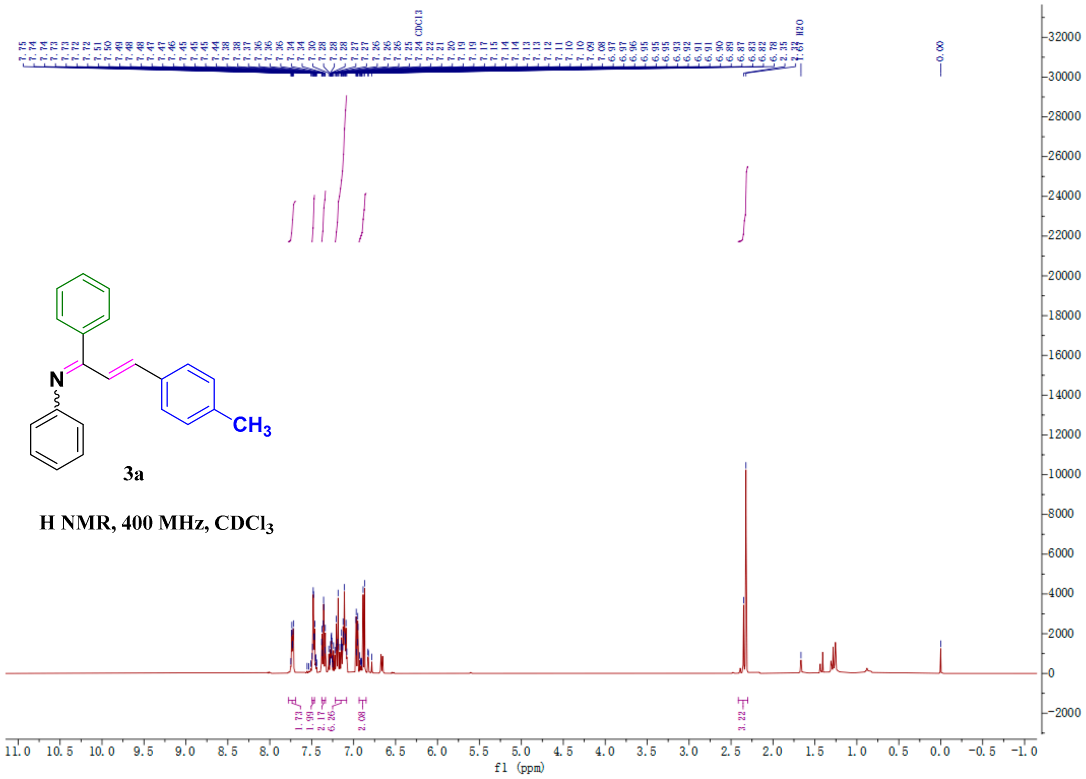
N
CH3
3a
H NMR, 400 MHz, CDCl3
1.73
1.99
2.17
6.26
2.08
3.22
0.00
f1 (ppm)

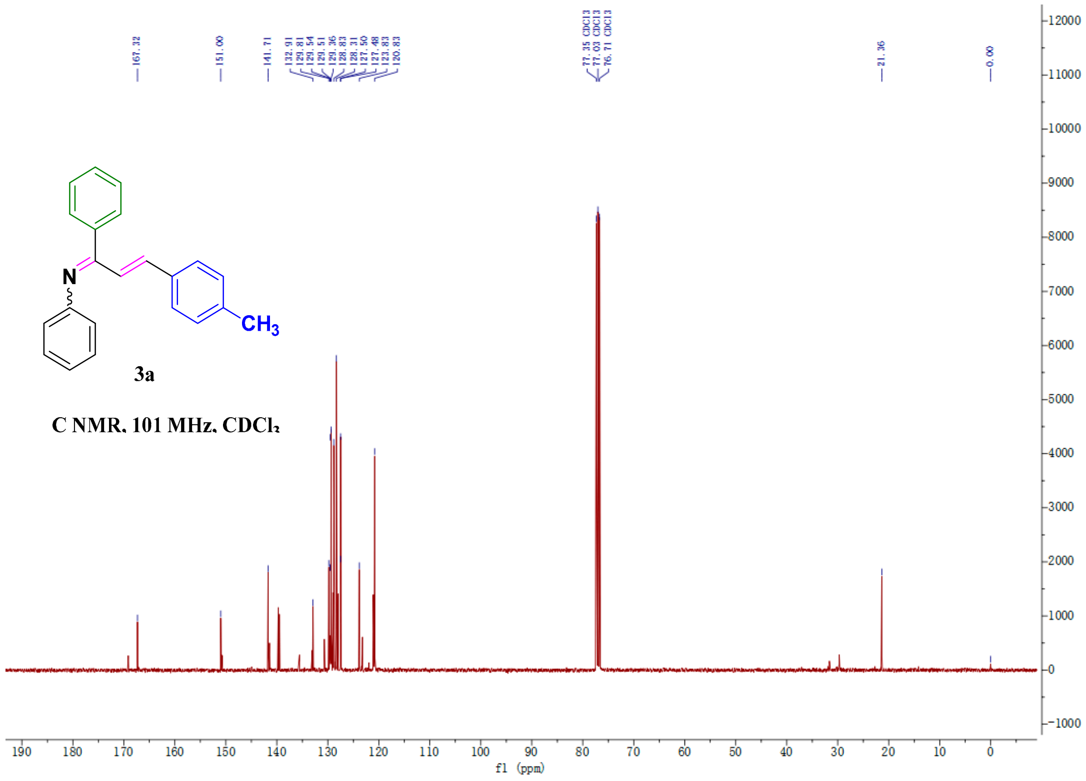
167.32
151.00
141.71
132.91
129.81
129.54
129.51
129.36
128.83
128.31
127.50
127.48
123.83
120.83
77.35 CDCl3
77.03 CDCl3
76.71 CDCl3
21.36
0.00
N
CH3
3a
C NMR, 101 MHz, CDCl3
f1 (ppm)

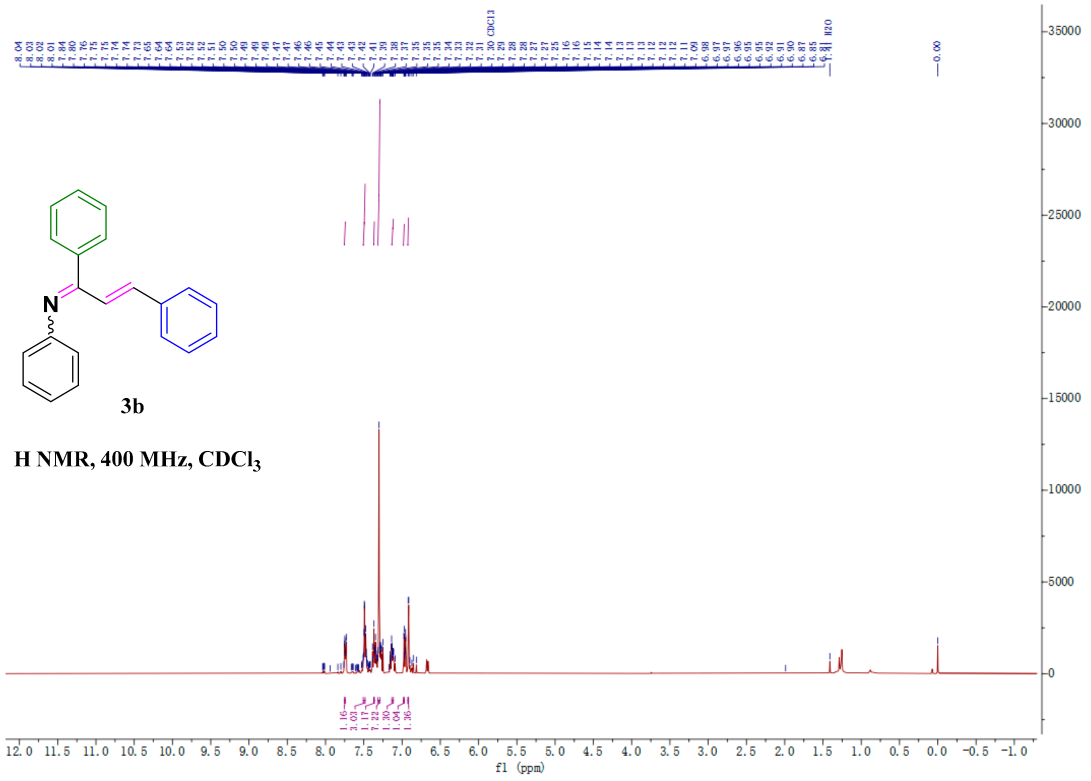

N
3b
H NMR, 400 MHz, CDCl3
CDCl3
H2O
f1 (ppm)

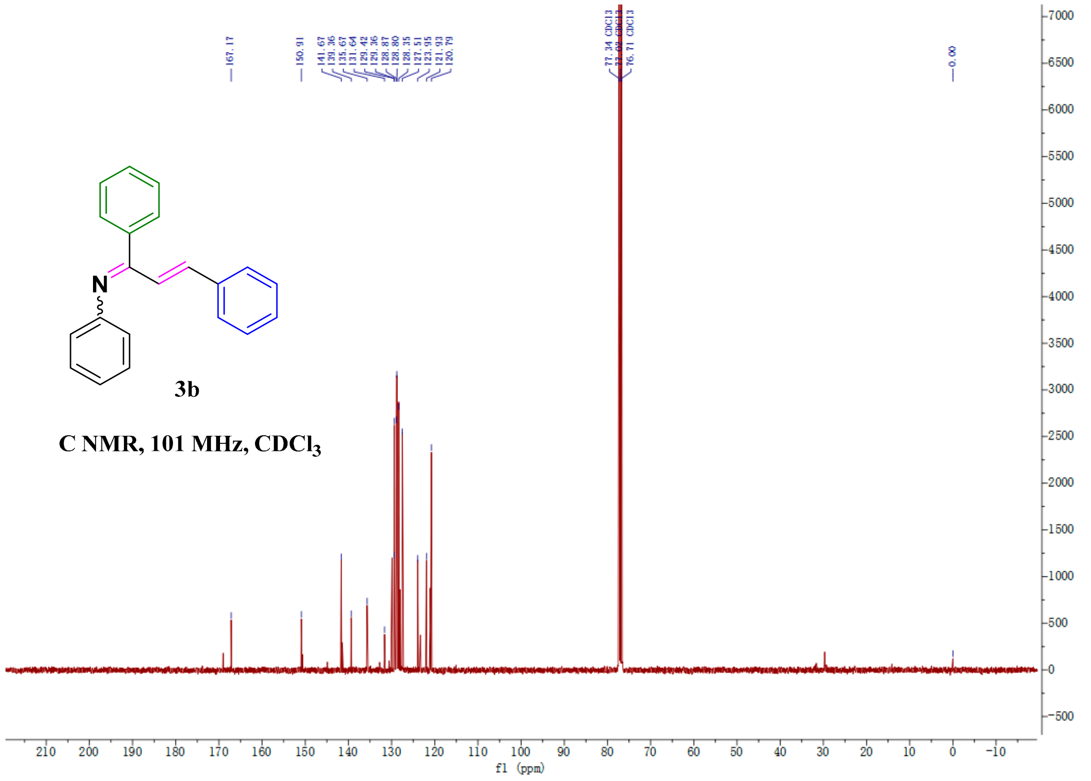

N
3b
C NMR, 101 MHz, CDCl3
167.17
150.91
141.67
139.36
135.67
131.64
129.42
129.36
128.87
128.80
128.35
127.51
123.95
121.93
120.79
77.34 CDCl3
76.71 CDCl3
0.00
f1 (ppm)

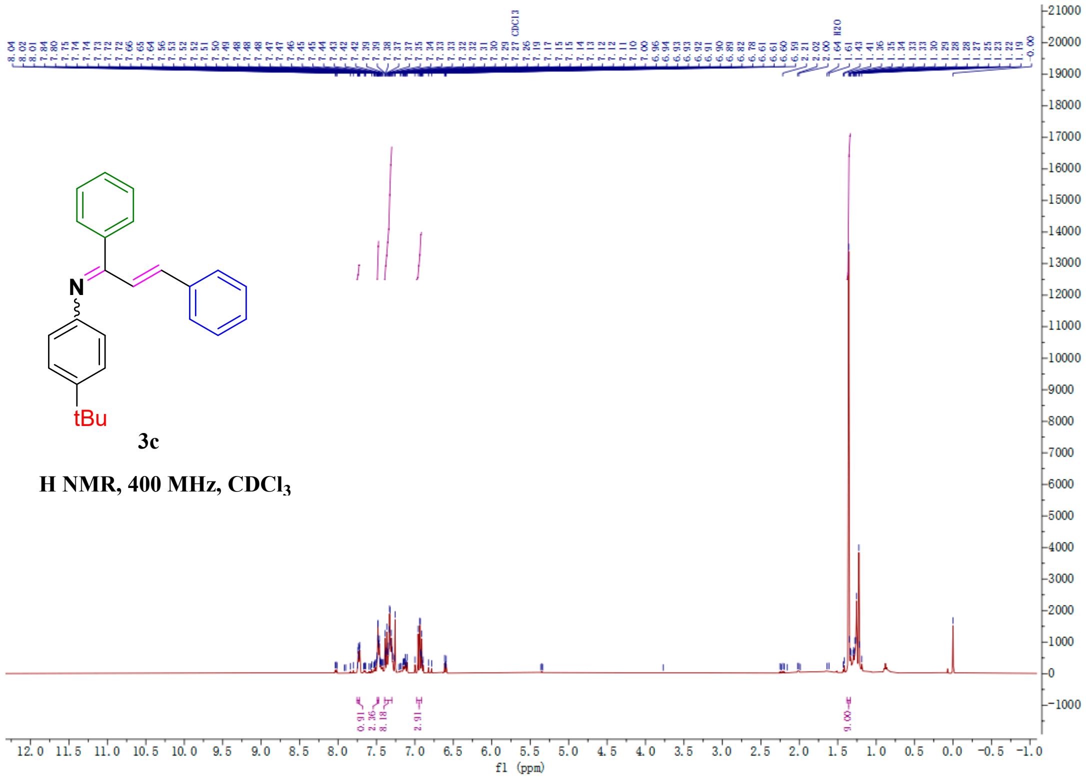

N
tBu
3c
H NMR, 400 MHz, CDCl$_3$
f1 (ppm)

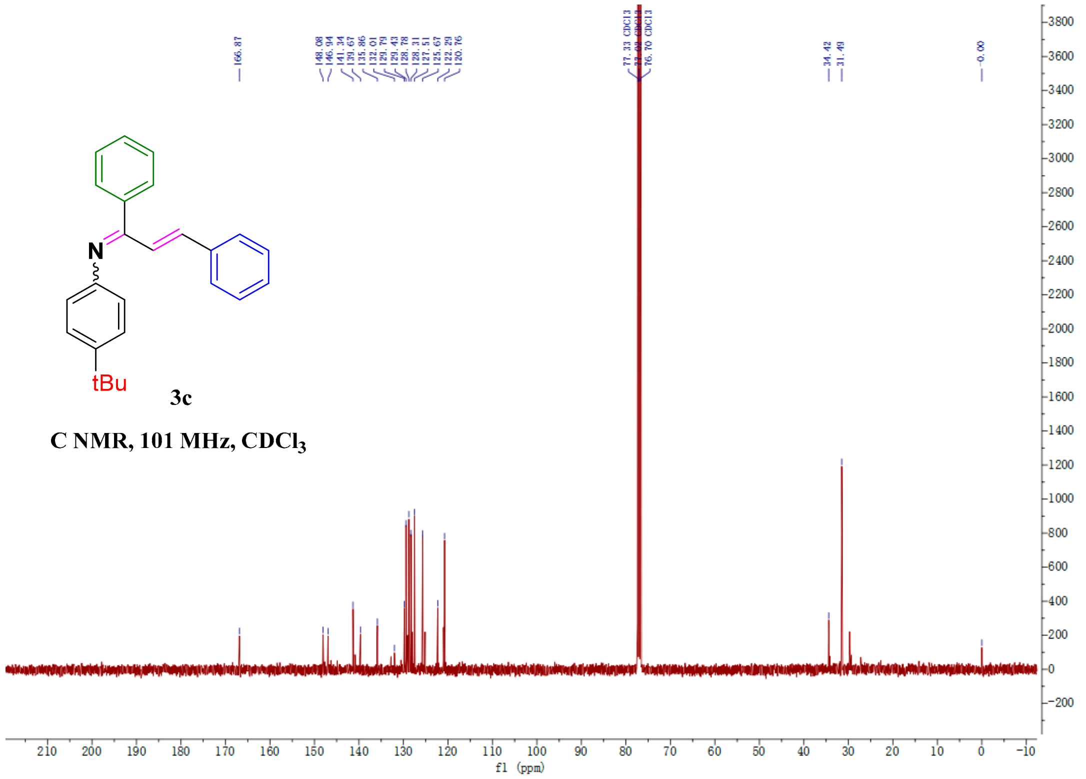

166.87
148.08
146.94
141.34
139.67
135.86
132.01
129.79
129.43
128.78
128.31
127.51
125.67
122.29
120.76
77.33 CDCl3
76.70 CDCl3
34.42
31.49
-0.00
N
tBu
3c
C NMR, 101 MHz, CDCl$_3$
f1 (ppm)

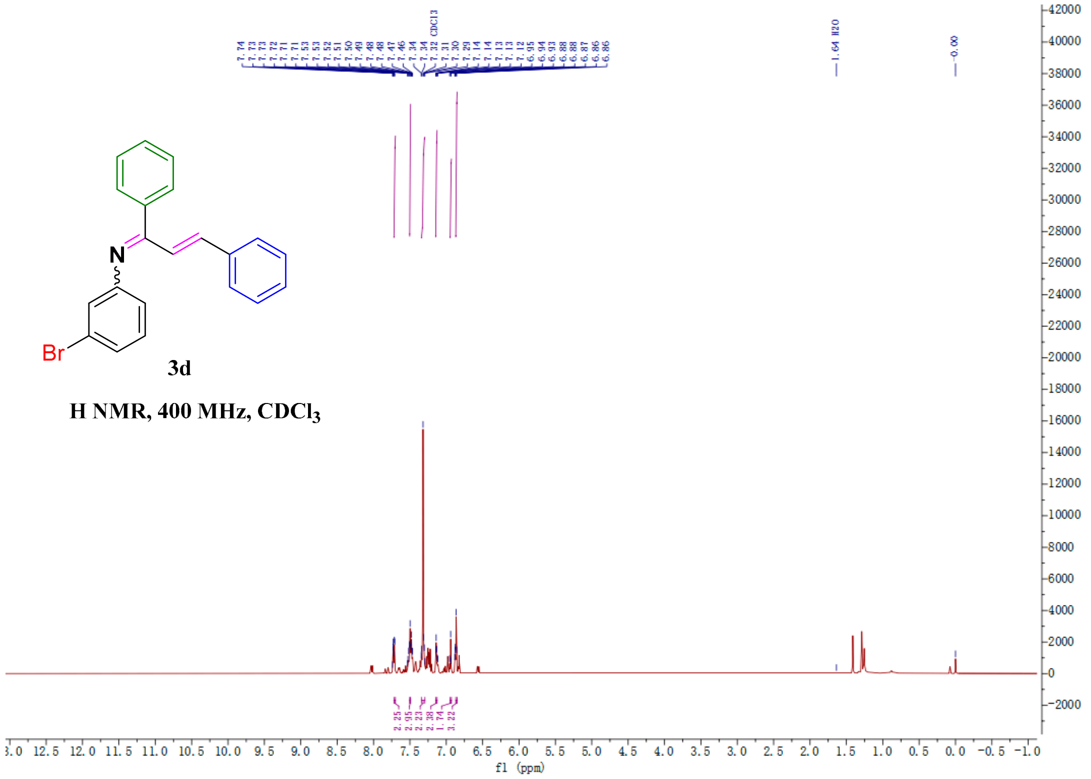

1.64 H2O
0.00
N
Br
3d
H NMR, 400 MHz, CDCl$_3$
f1 (ppm)

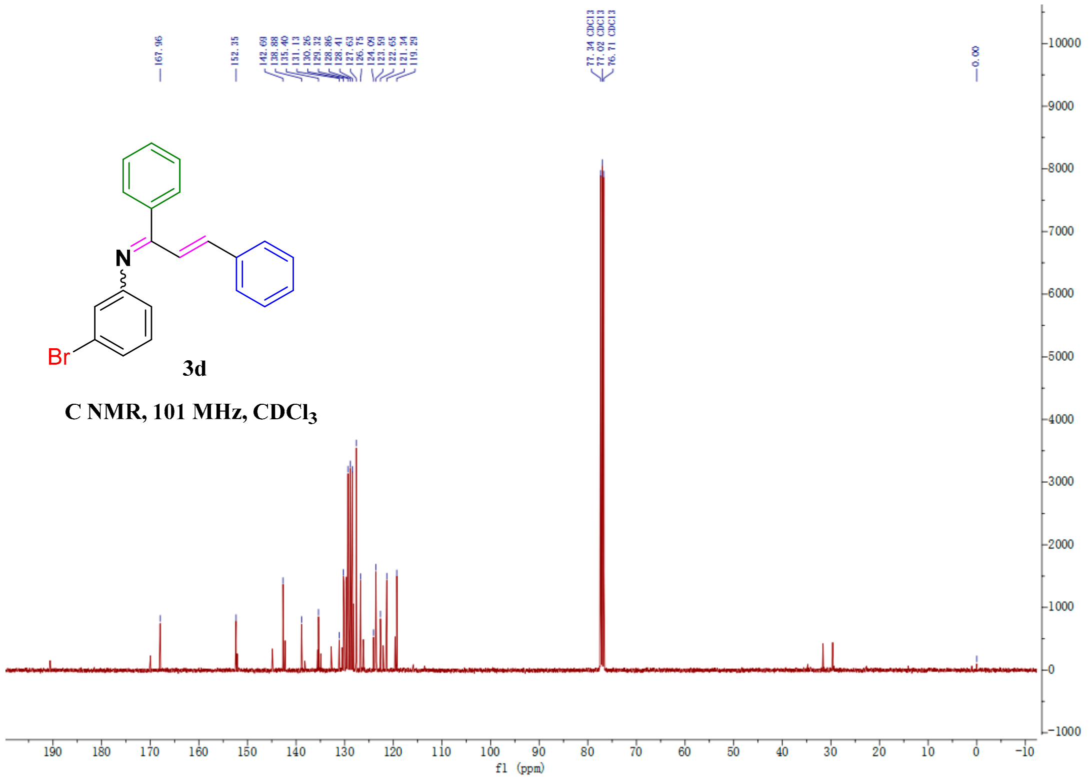

167.96
152.35
142.69
138.88
135.40
131.13
130.26
129.32
128.86
128.41
127.63
126.75
124.09
123.59
122.65
121.34
119.29
77.34 CDCl3
77.02 CDCl3
76.71 CDCl3
0.00
N
Br
3d
C NMR, 101 MHz, CDCl$_3$
f1 (ppm)

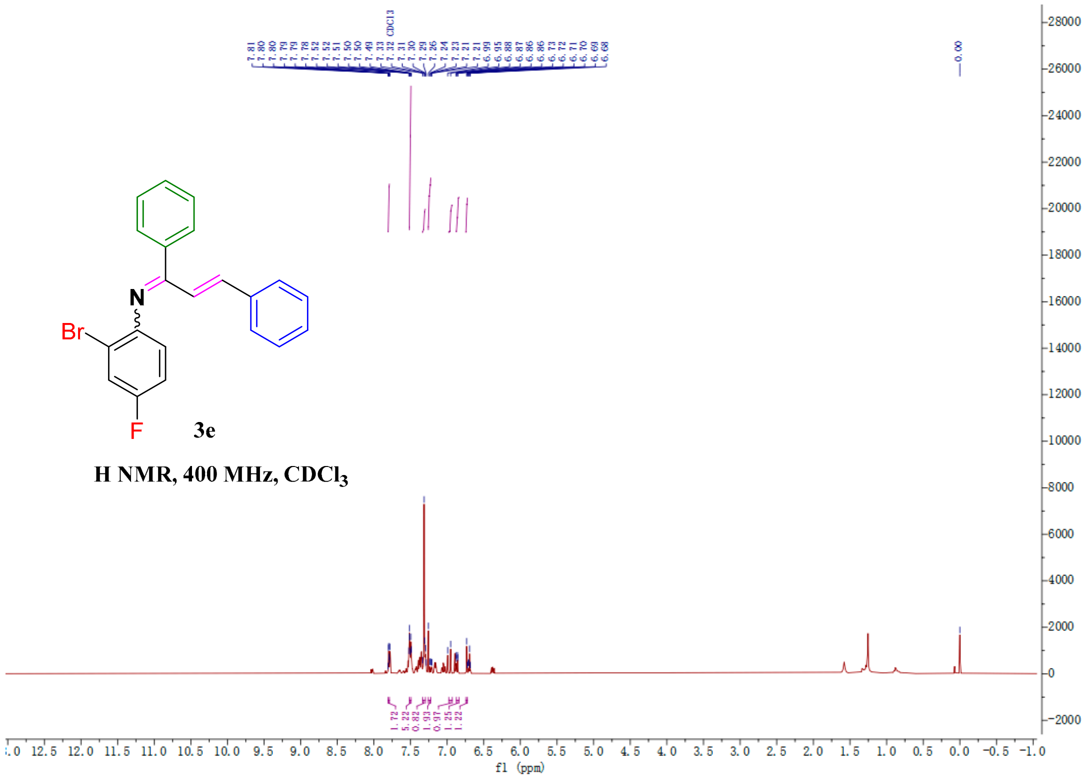
N
Br
F
3e
H NMR, 400 MHz, CDCl3
f1 (ppm)

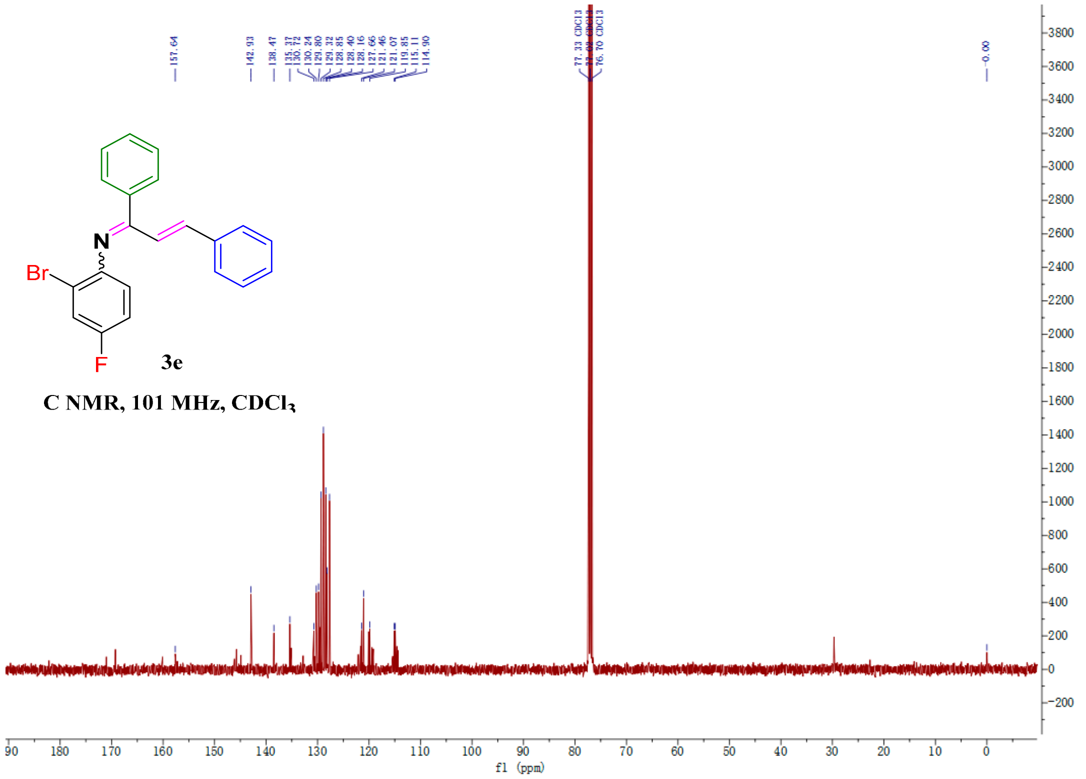
N
Br
F
3e
C NMR, 101 MHz, CDCl3
f1 (ppm)

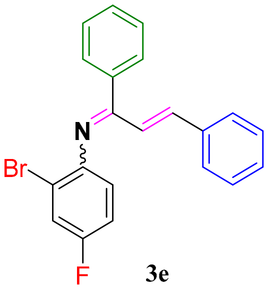

3e

F NMR, 376 MHz, $CDCl_3$

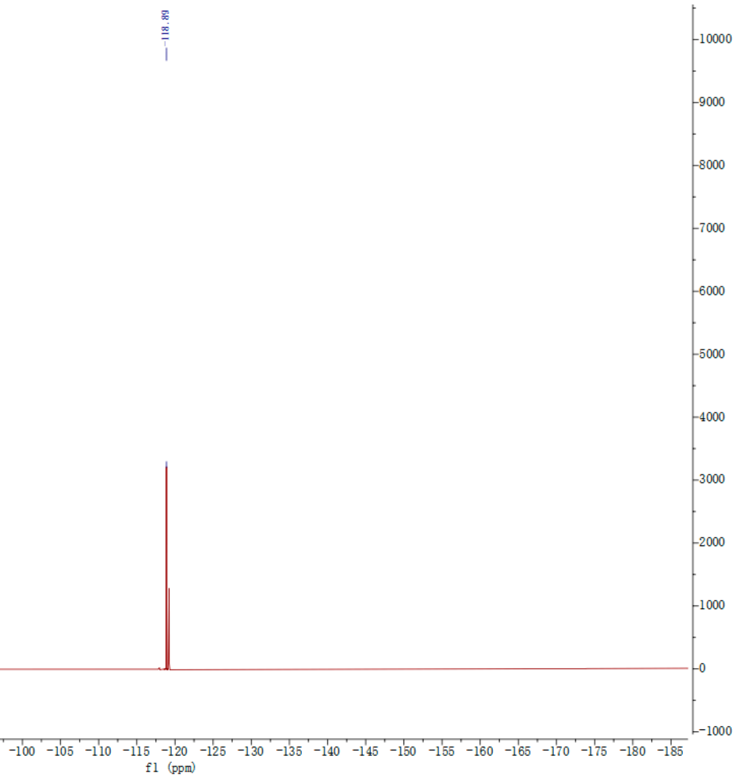

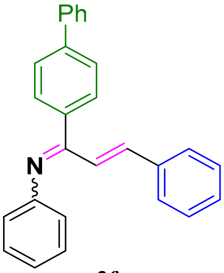

3f

H NMR, 400 MHz, $CDCl_3$

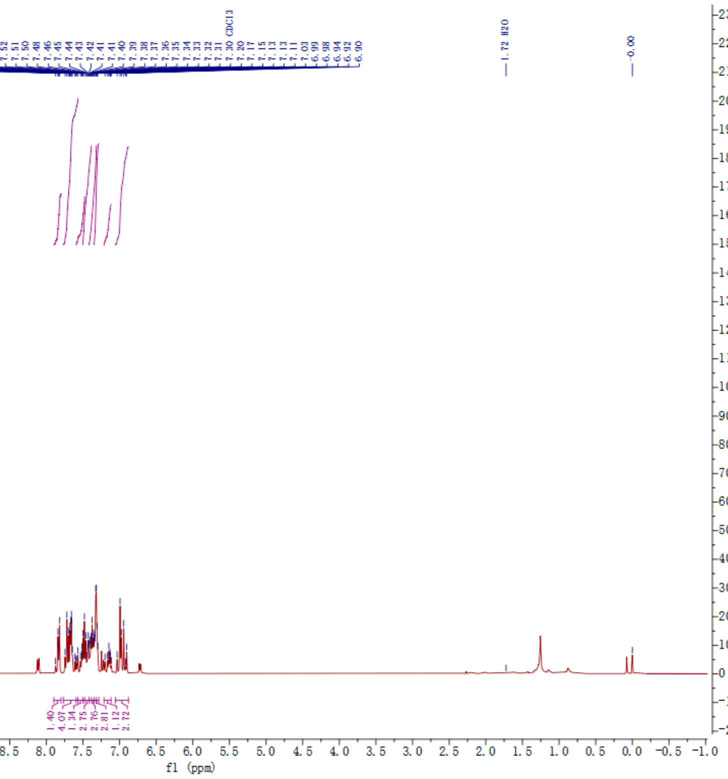

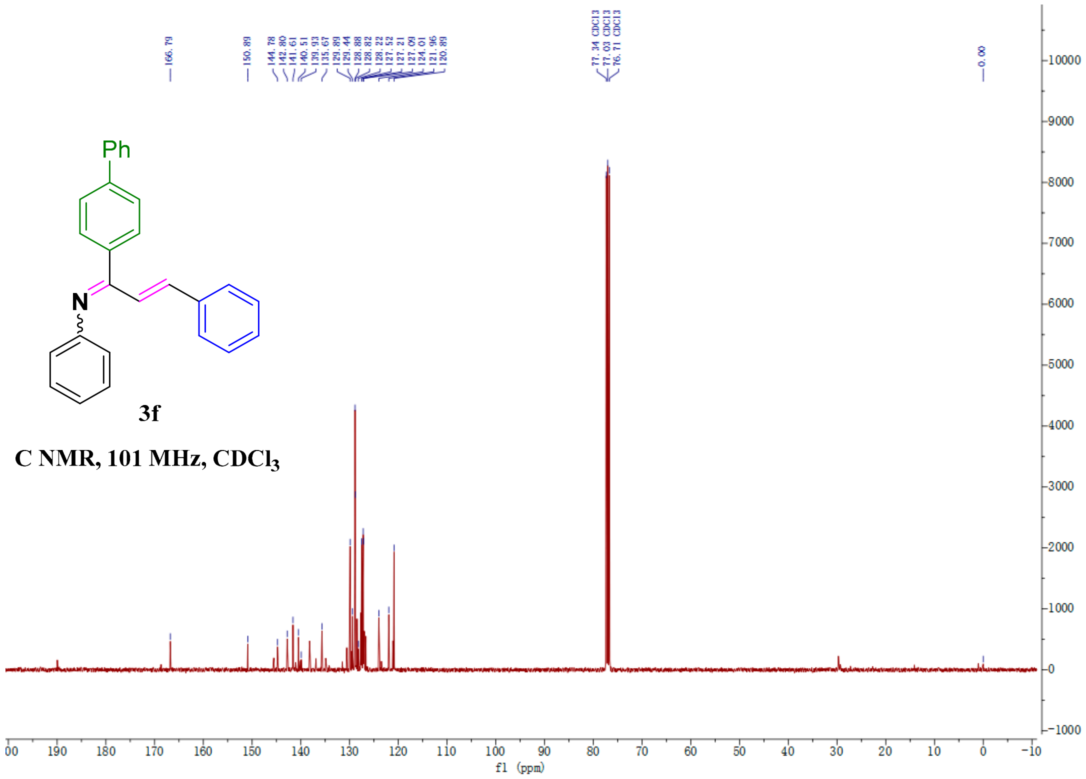
Ph
N
3f
C NMR, 101 MHz, CDCl3
f1 (ppm)

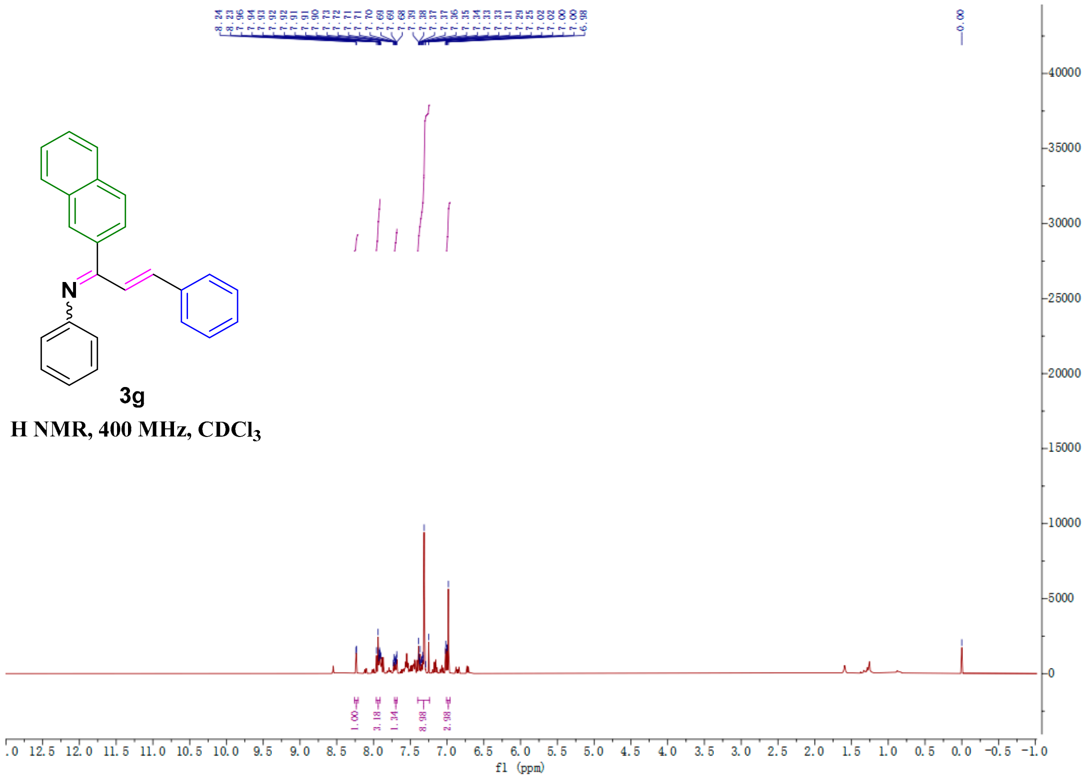
N
3g
H NMR, 400 MHz, CDCl3
f1 (ppm)

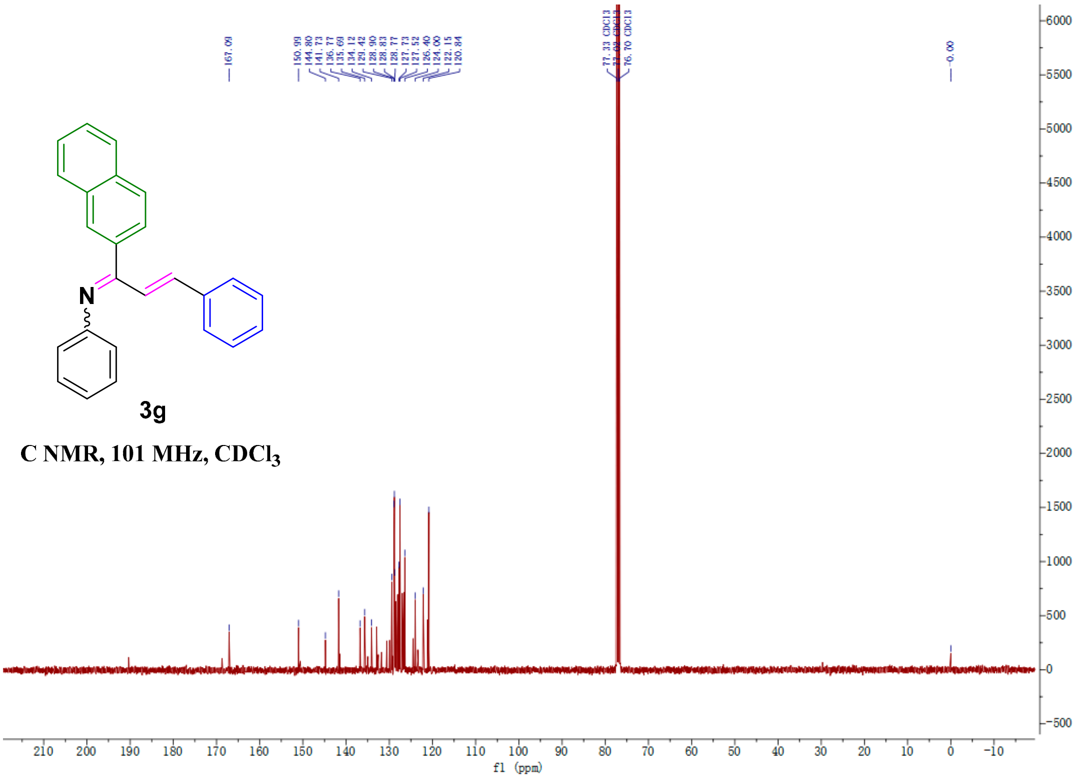

3g

C NMR, 101 MHz, $CDCl_3$

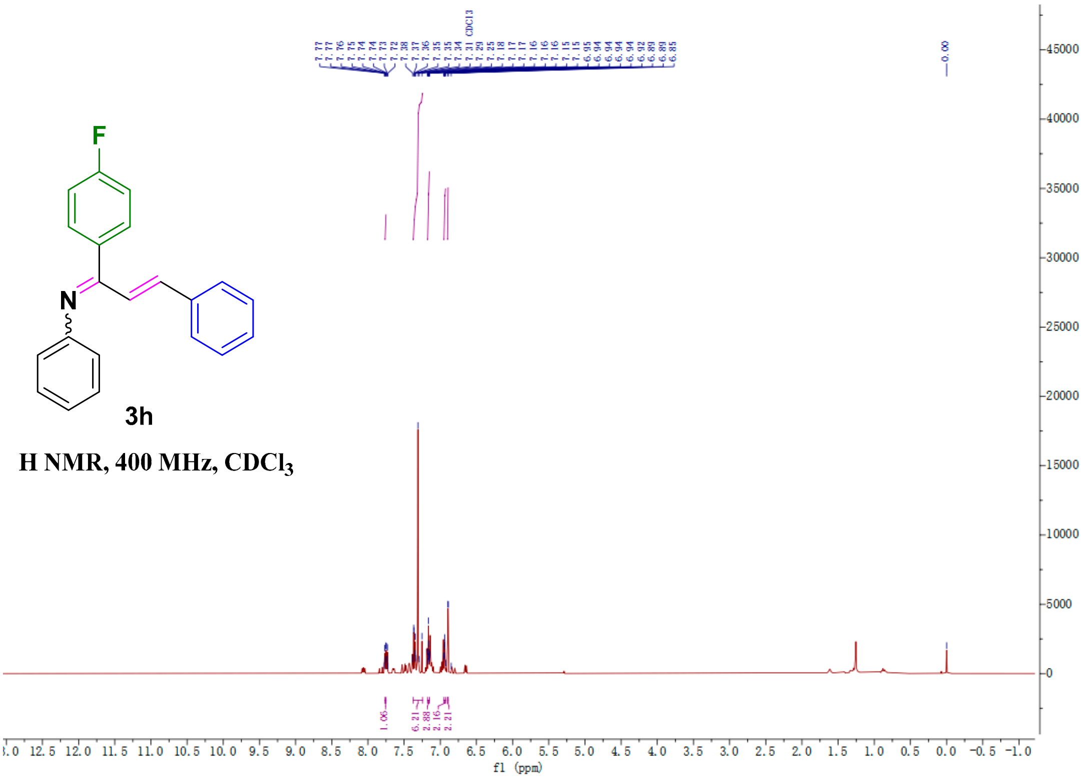

3h

H NMR, 400 MHz, $CDCl_3$

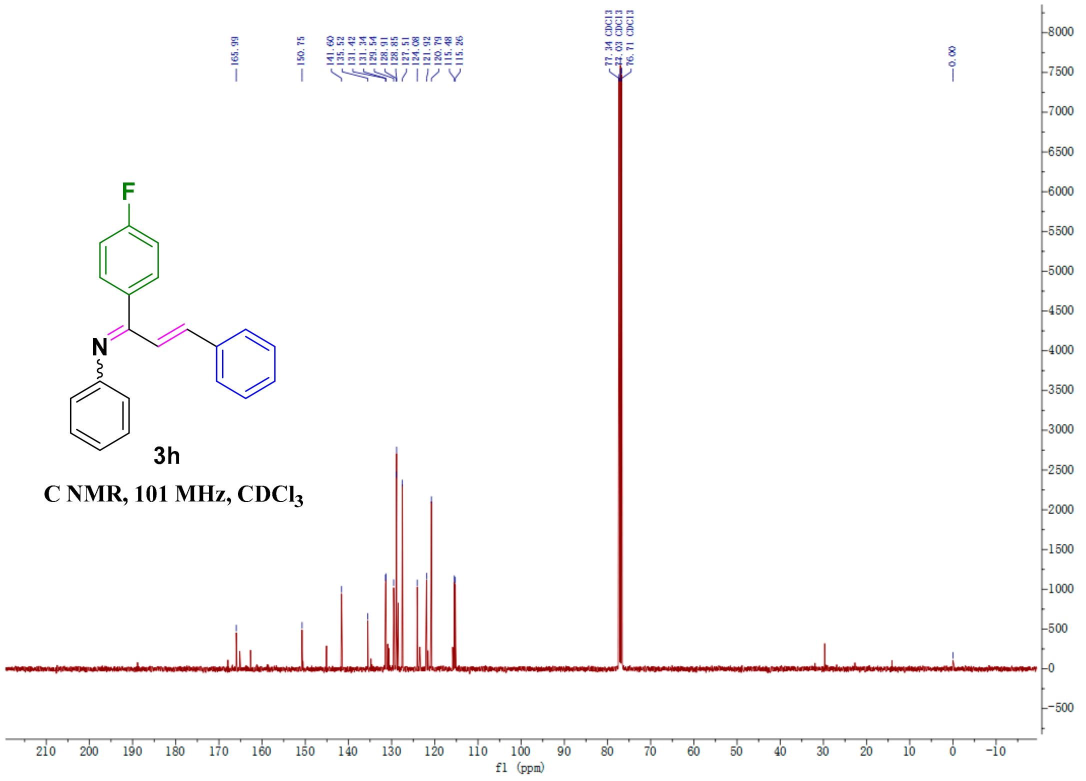
165.99
150.75
141.60
135.52
131.42
131.34
129.54
128.91
128.85
127.51
124.08
121.92
120.79
115.48
115.26
77.34 CDCl3
77.03 CDCl3
76.71 CDCl3
0.00
F
N
3h
C NMR, 101 MHz, CDCl3
f1 (ppm)

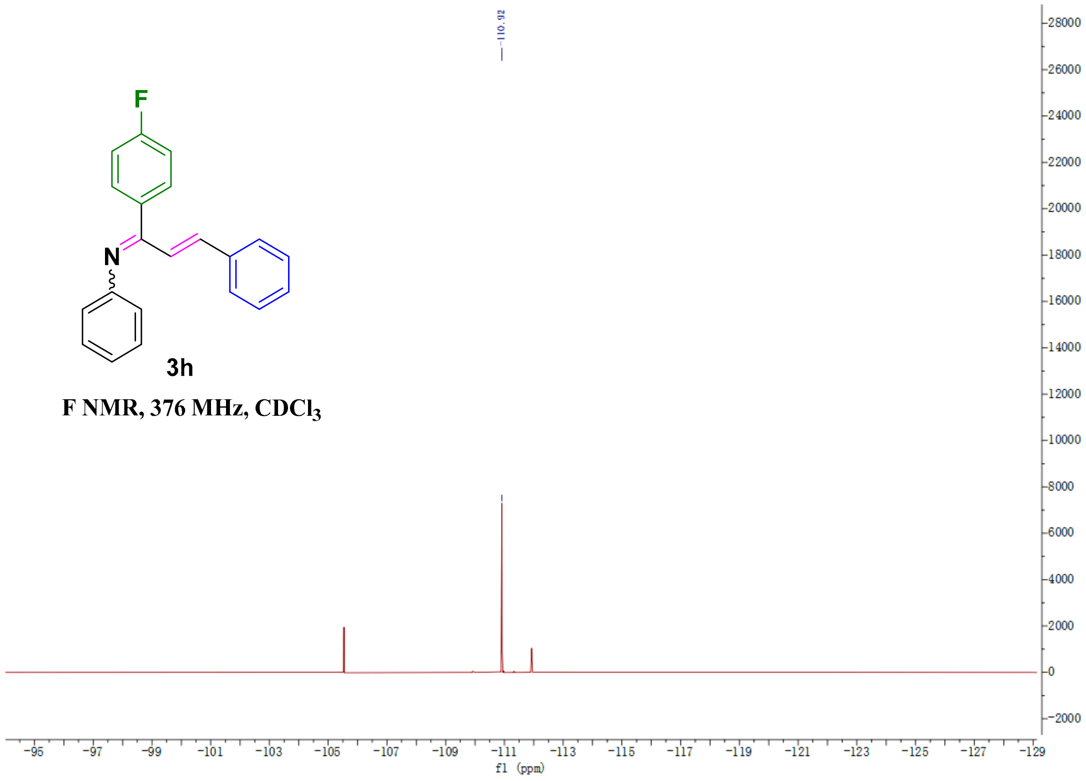
-110.92
F
N
3h
F NMR, 376 MHz, CDCl3
f1 (ppm)

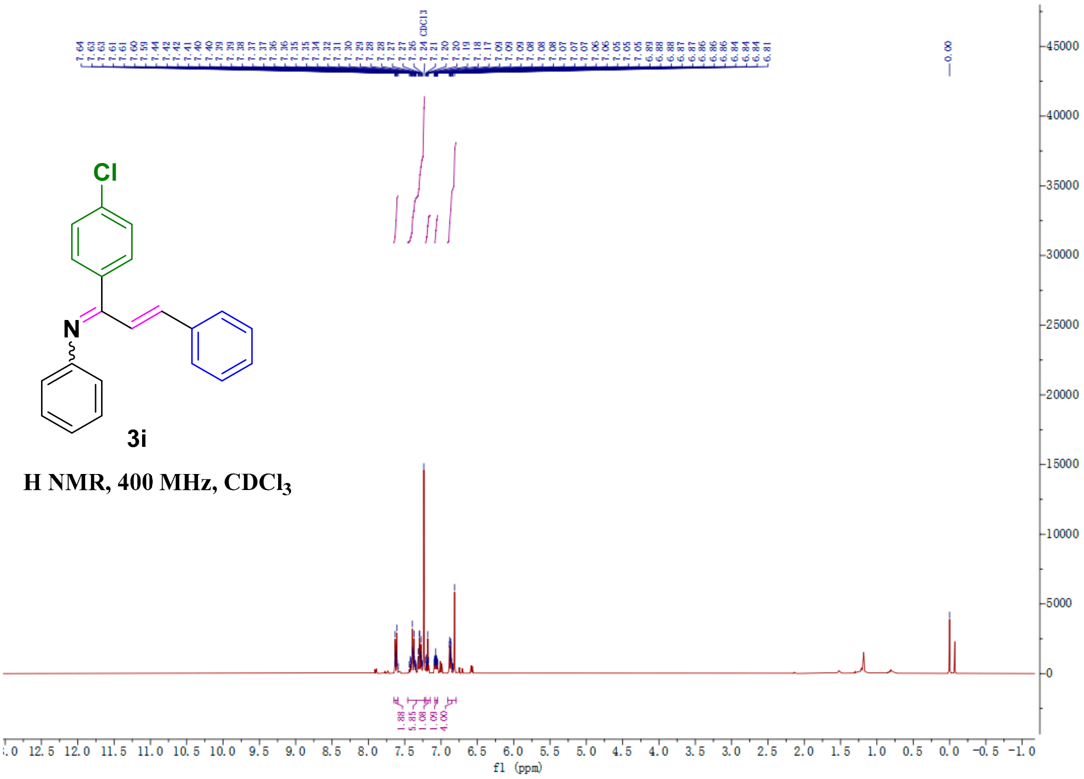

3i
H NMR, 400 MHz, CDCl3
1.88
5.85
1.08
1.09
4.00
0.00
f1 (ppm)

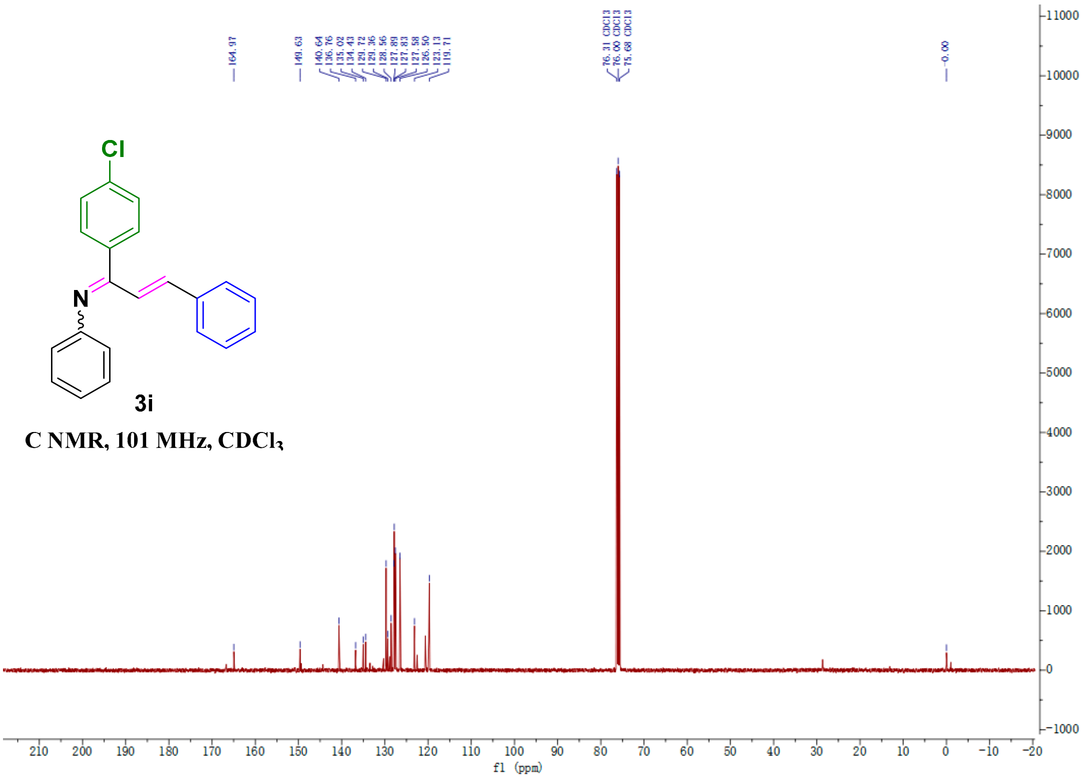

3i
C NMR, 101 MHz, CDCl3
164.97
149.63
140.64
136.76
135.02
134.43
129.72
129.36
128.56
127.89
127.83
127.58
126.50
123.13
119.71
76.31 CDCl3
76.00 CDCl3
75.68 CDCl3
0.00
f1 (ppm)

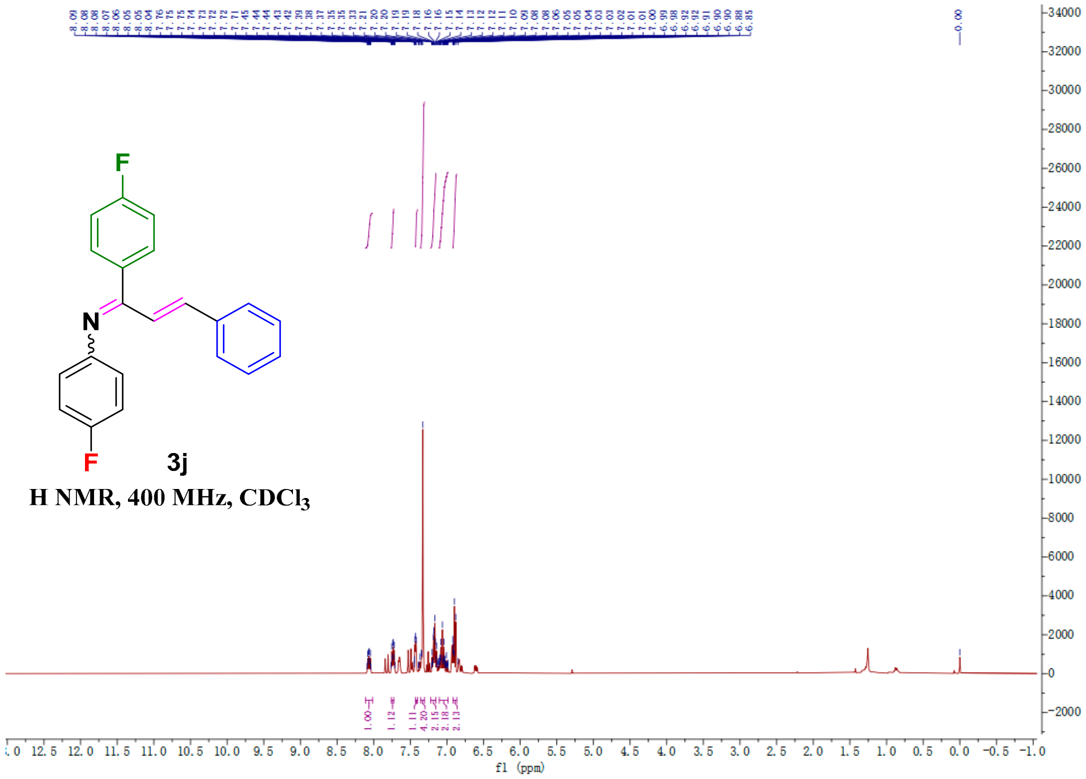

F
N
F
3j
H NMR, 400 MHz, CDCl3
f1 (ppm)

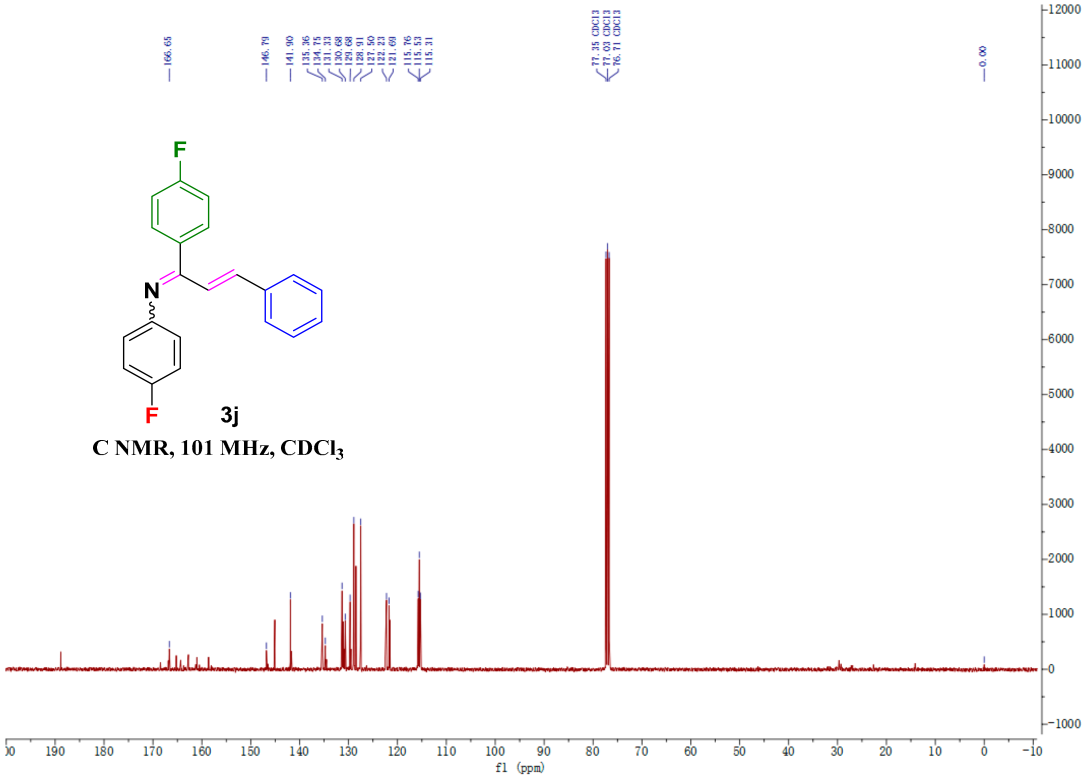

166.65
146.79
141.90
135.36
134.75
131.31
130.68
129.68
128.91
127.50
122.23
121.69
115.76
115.53
115.31
77.35 CDCl3
77.03 CDCl3
76.71 CDCl3
0.00
F
N
F
3j
C NMR, 101 MHz, CDCl3
f1 (ppm)

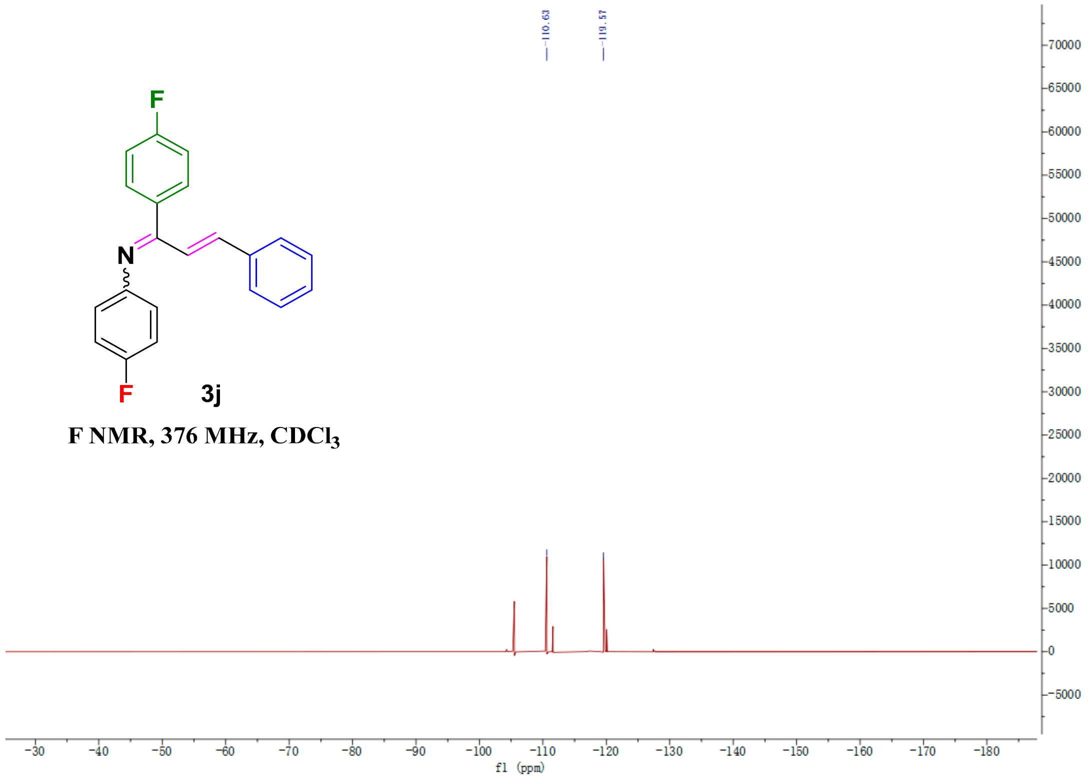

3j

F NMR, 376 MHz, $CDCl_3$

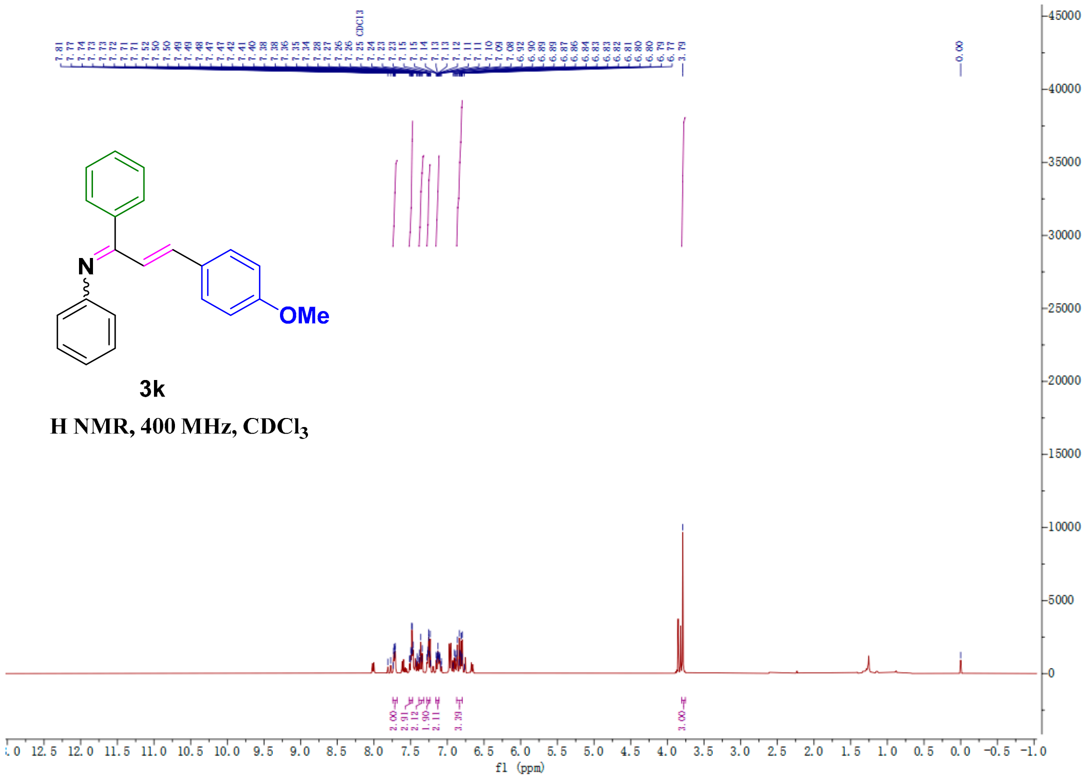

3k

H NMR, 400 MHz, $CDCl_3$

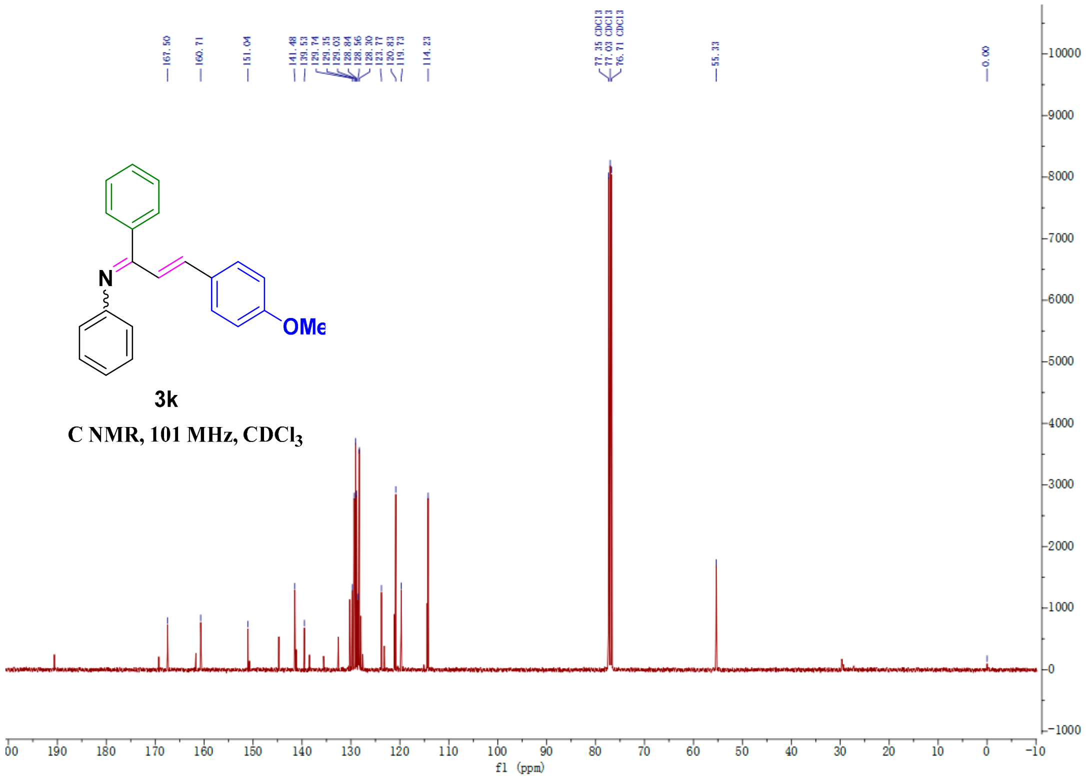

167.50
160.71
151.04
141.48
139.53
129.74
129.35
129.03
128.84
128.56
128.30
123.77
120.83
119.73
114.23
77.35 CDCl3
77.03 CDCl3
76.71 CDCl3
55.33
0.00
N
OMe
3k
C NMR, 101 MHz, CDCl3
f1 (ppm)

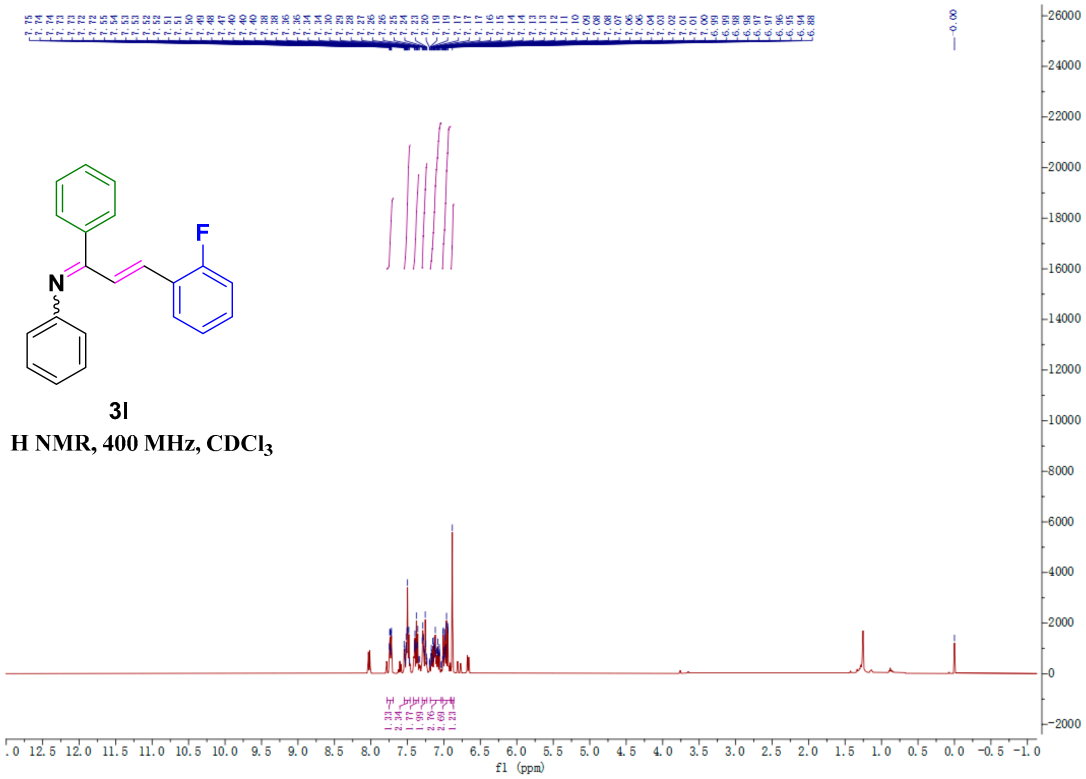

0.00
N
F
3l
H NMR, 400 MHz, CDCl3
f1 (ppm)

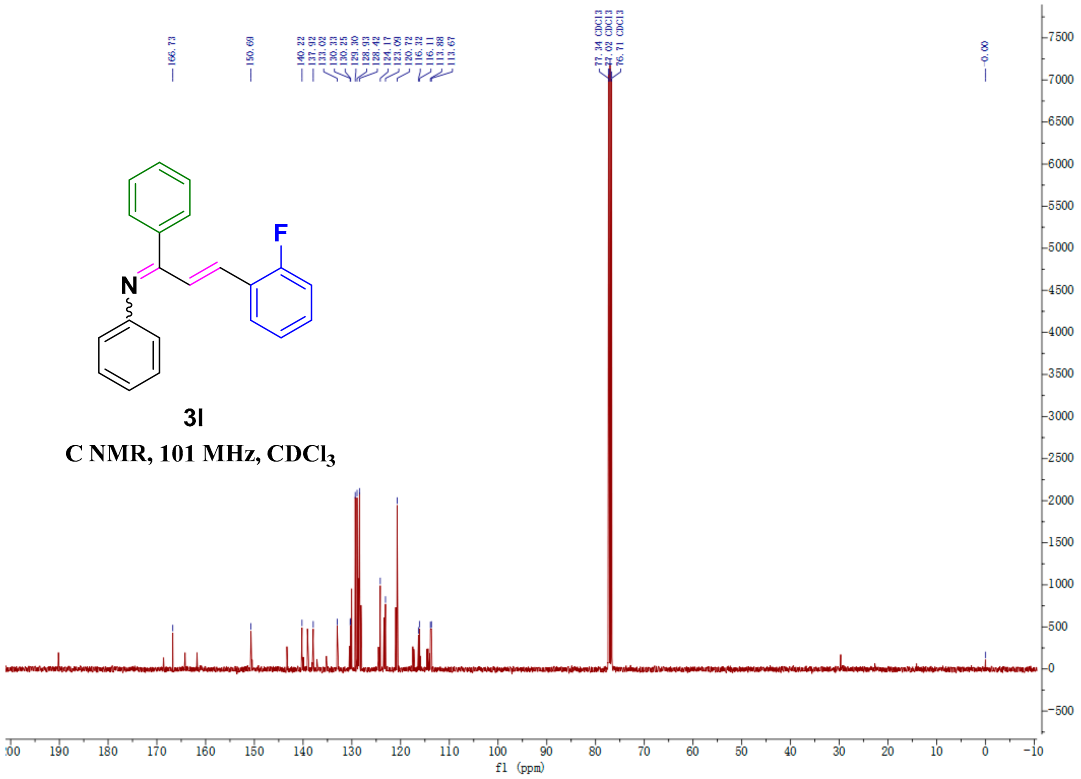

166.73
150.69
140.22
137.92
133.02
130.33
130.25
129.30
128.93
128.42
124.17
123.09
120.72
116.32
116.11
113.88
113.67
77.34 CDCl3
77.02 CDCl3
76.71 CDCl3
-0.00
F
N
3l
C NMR, 101 MHz, CDCl3
f1 (ppm)

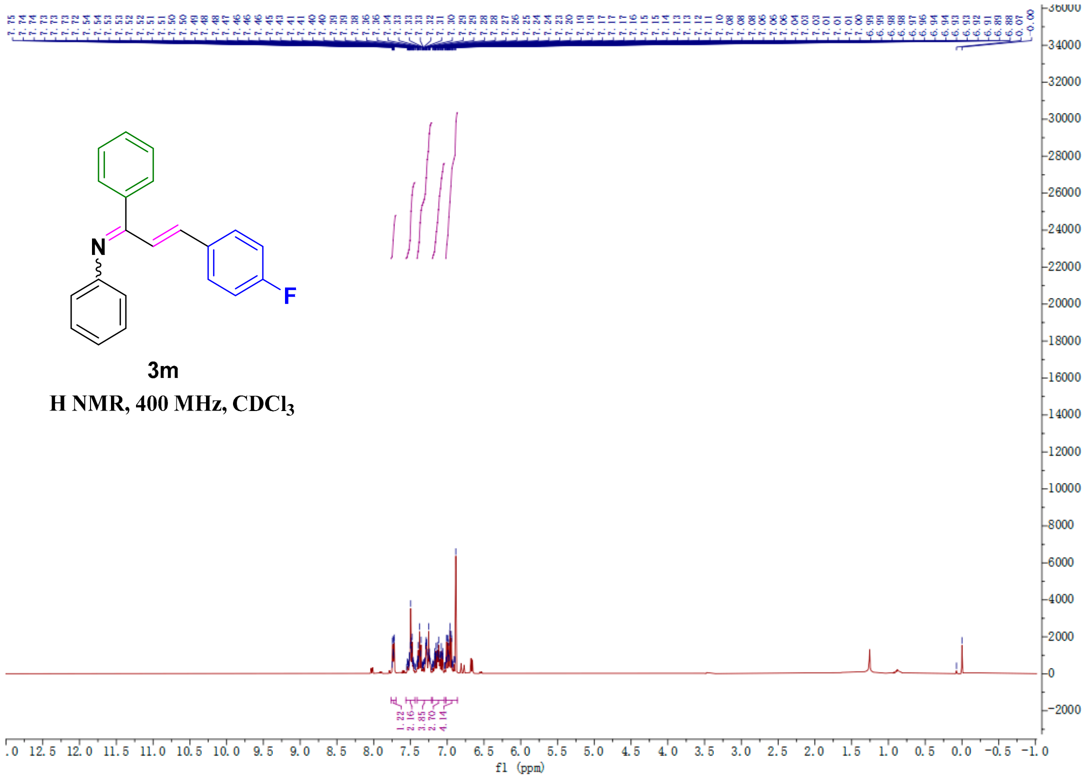

N
F
3m
H NMR, 400 MHz, CDCl3
1.22
2.16
3.85
2.70
4.14
f1 (ppm)

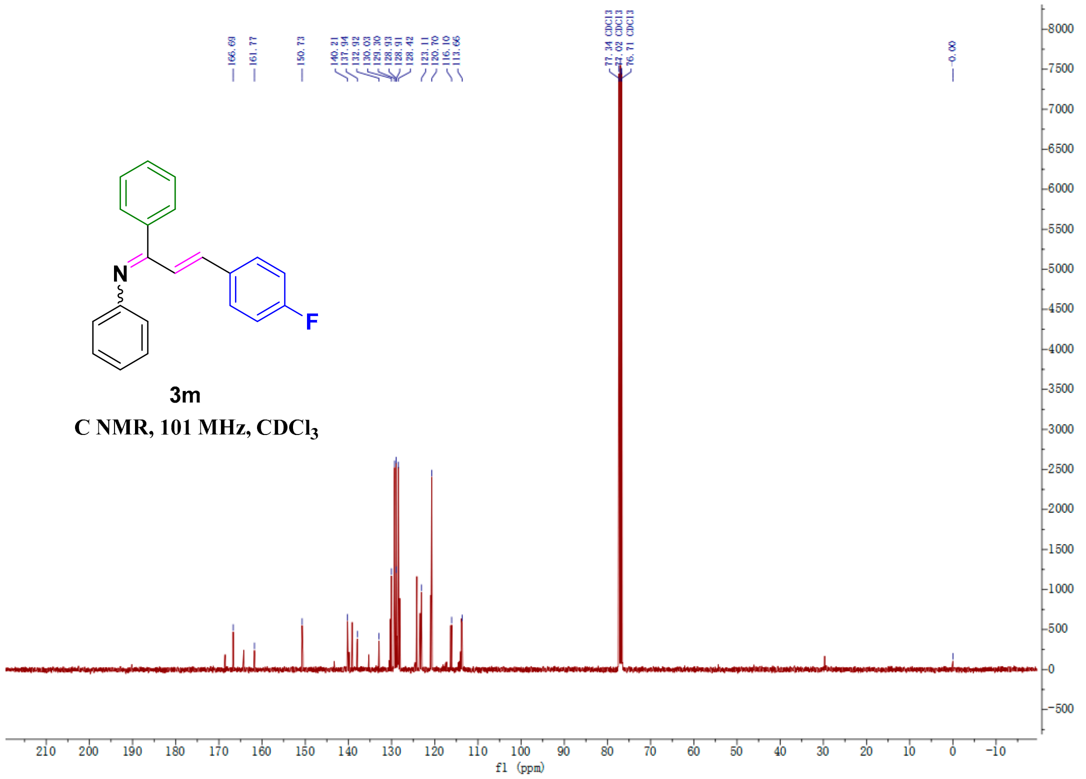
166.69
161.77
150.73
140.21
137.94
132.92
130.03
129.30
128.93
128.91
128.42
123.11
120.70
116.10
113.66
77.34 CDCl3
77.02 CDCl3
76.71 CDCl3
0.00
N
F
3m
C NMR, 101 MHz, CDCl3
f1 (ppm)

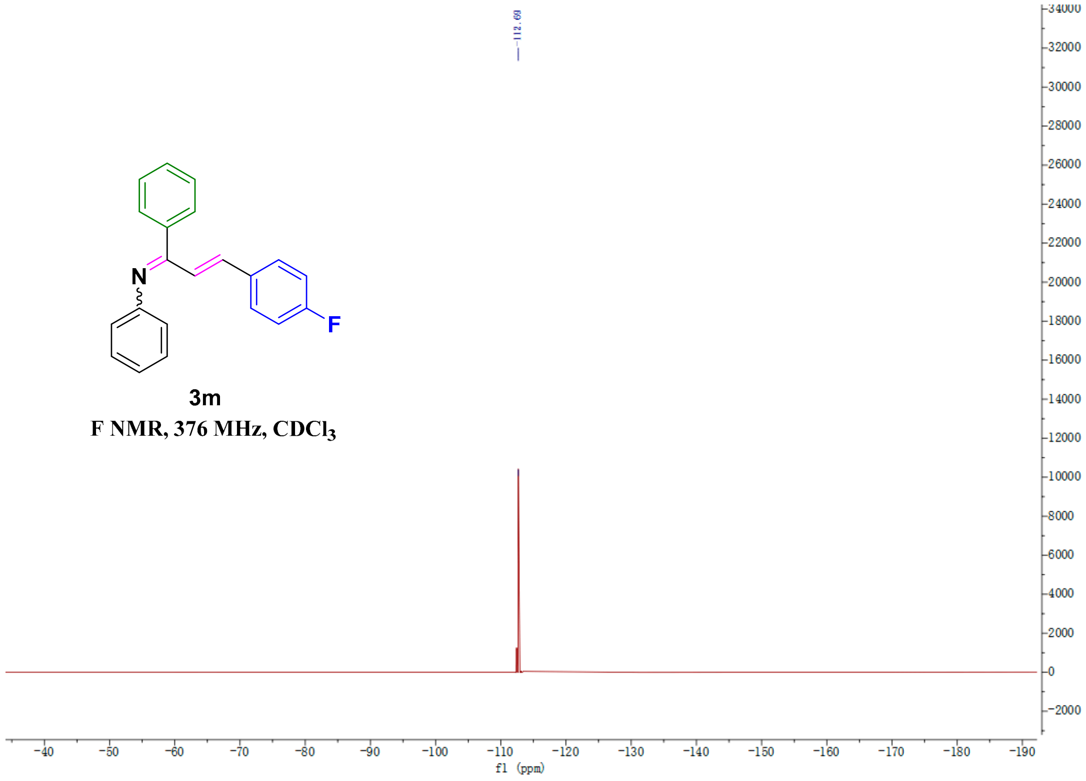
-112.69
N
F
3m
F NMR, 376 MHz, CDCl3
f1 (ppm)

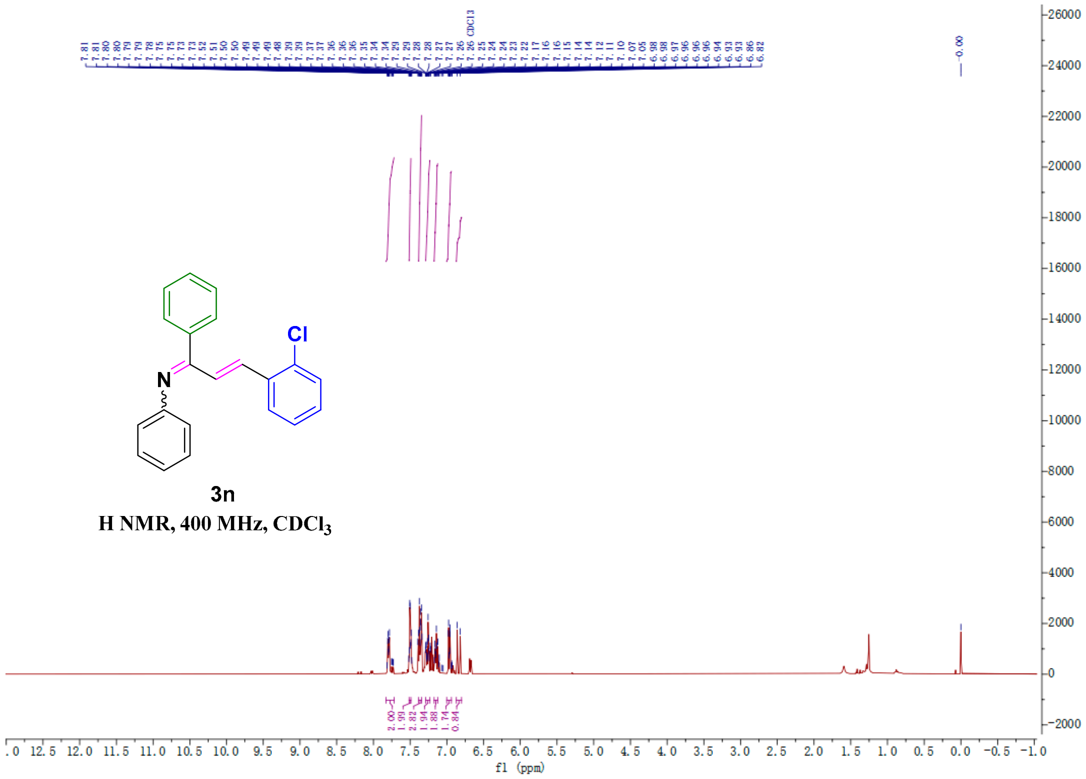
Cl
N
3n
H NMR, 400 MHz, CDCl3
f1 (ppm)

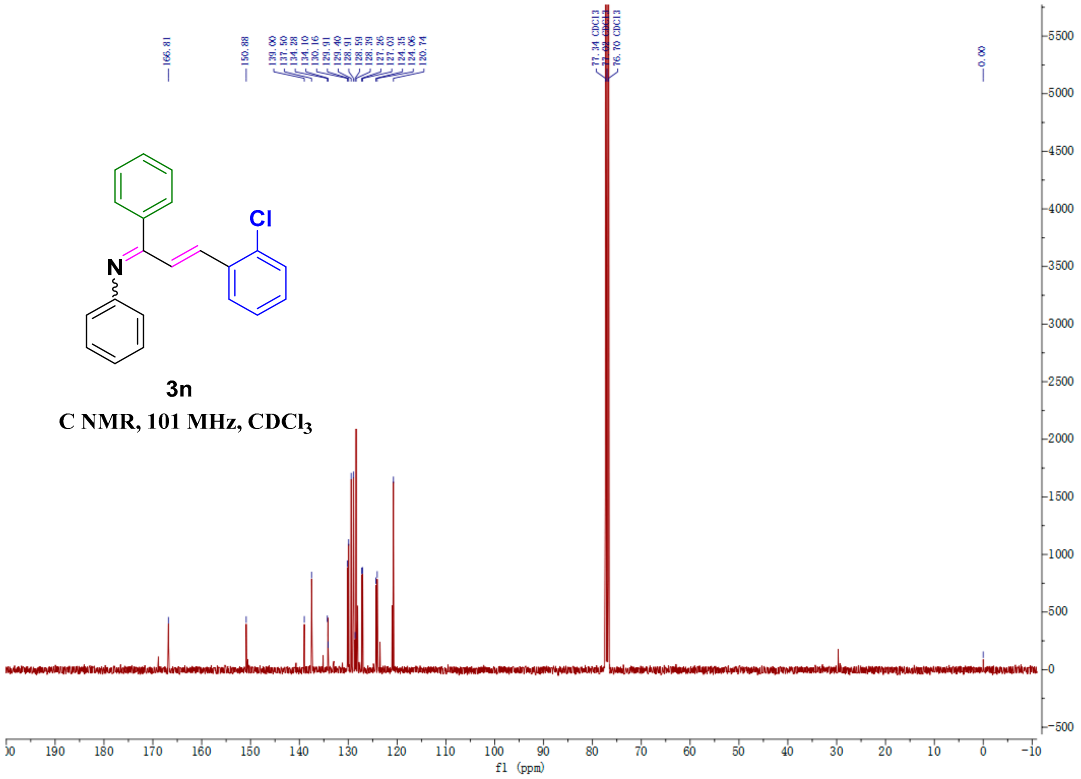
166.81
150.88
139.00
137.50
134.28
134.16
130.16
129.91
129.40
128.91
128.59
128.39
127.35
127.03
124.35
124.06
120.74
77.34 CDCl3
77.02 CDCl3
76.70 CDCl3
0.00
Cl
N
3n
C NMR, 101 MHz, CDCl3
f1 (ppm)

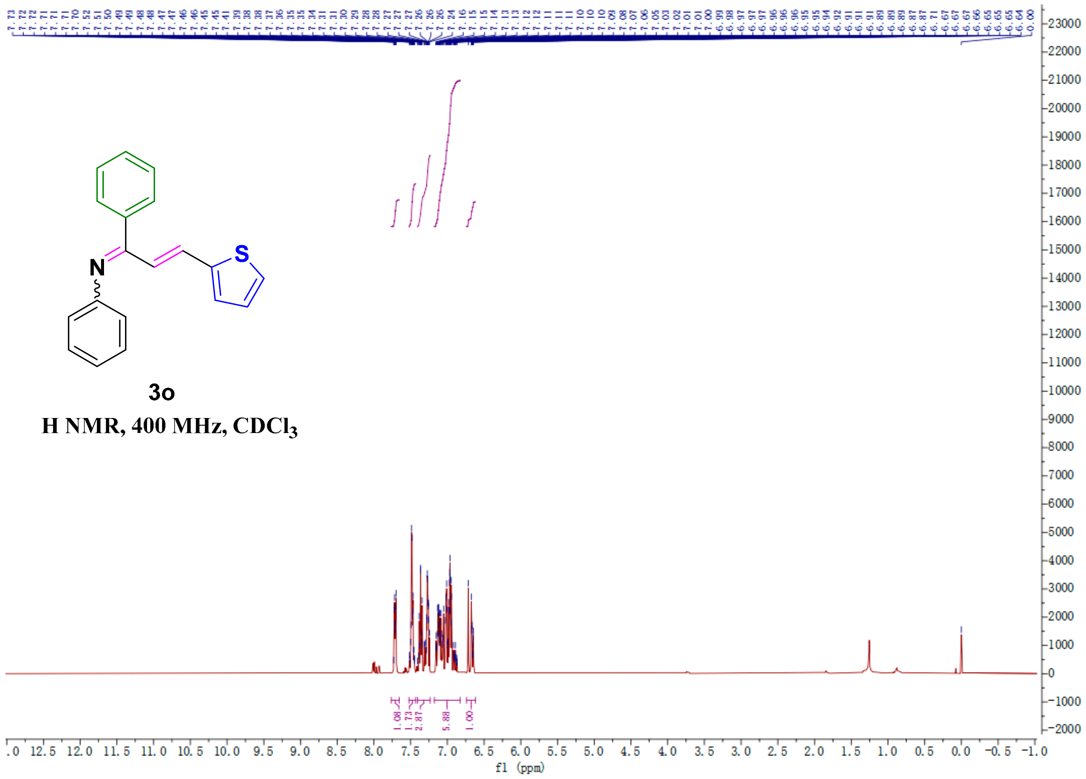
3o
H NMR, 400 MHz, CDCl3
f1 (ppm)

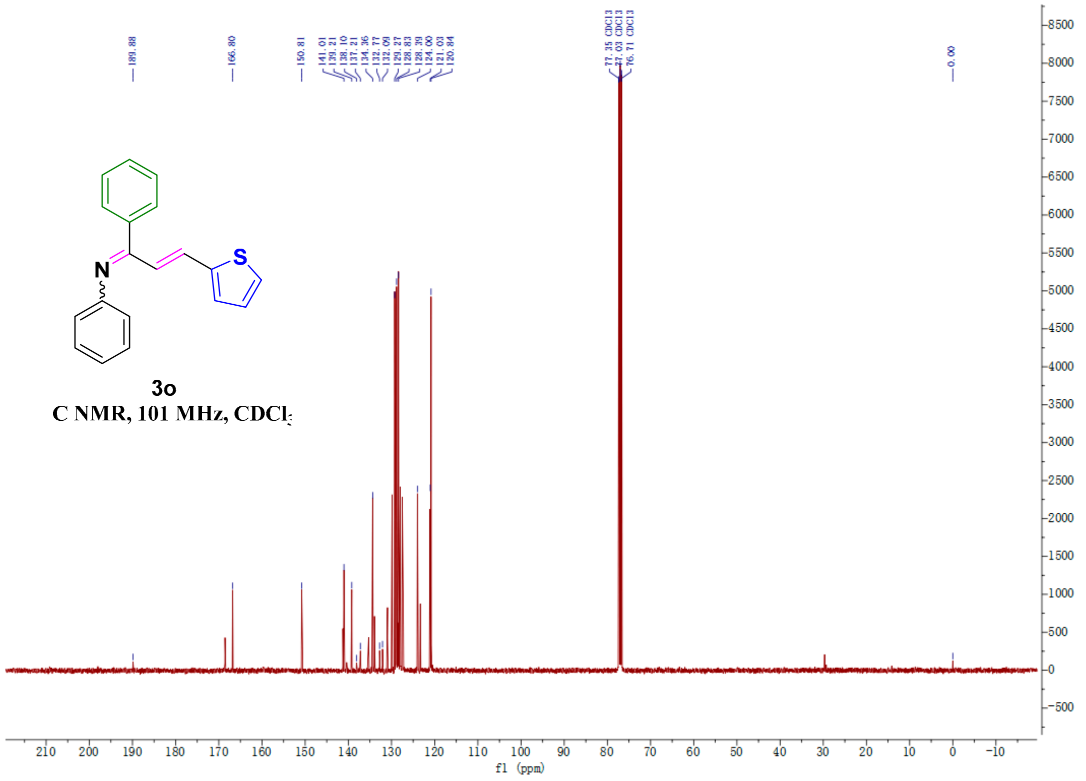
3o
C NMR, 101 MHz, CDCl3
f1 (ppm)

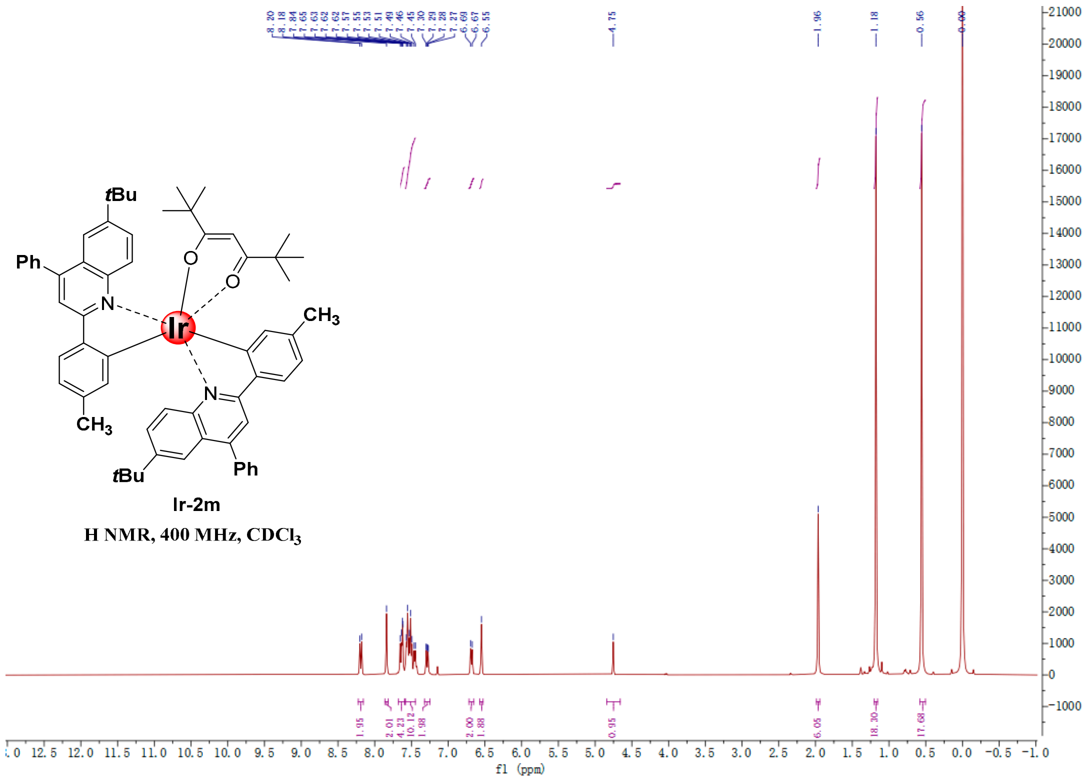

Ir-2m

H NMR, 400 MHz, $CDCl_3$

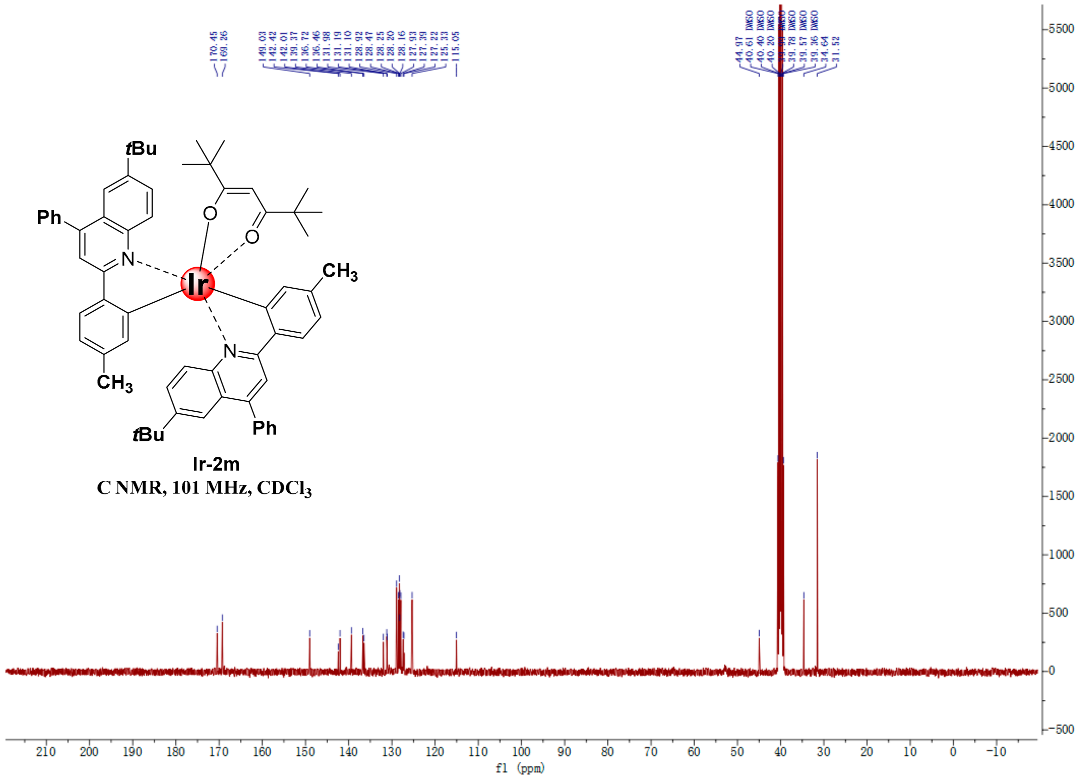

Ir-2m

C NMR, 101 MHz, $CDCl_3$

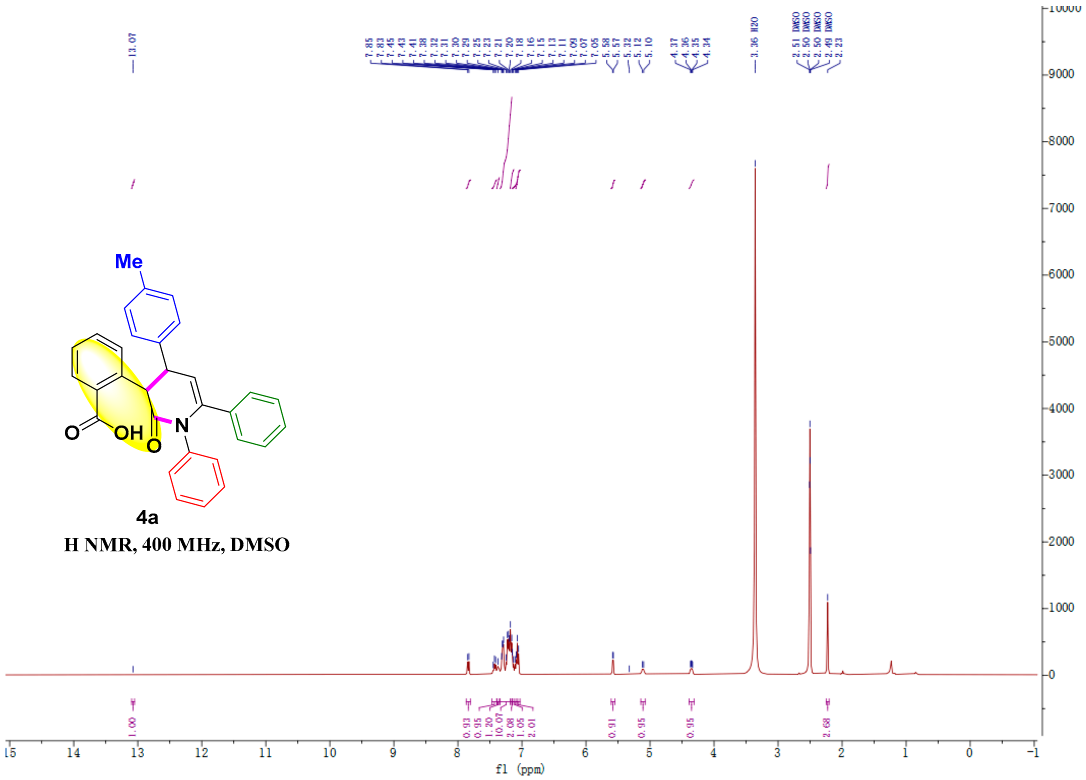
Me
OH
O
N
4a
H NMR, 400 MHz, DMSO
f1 (ppm)

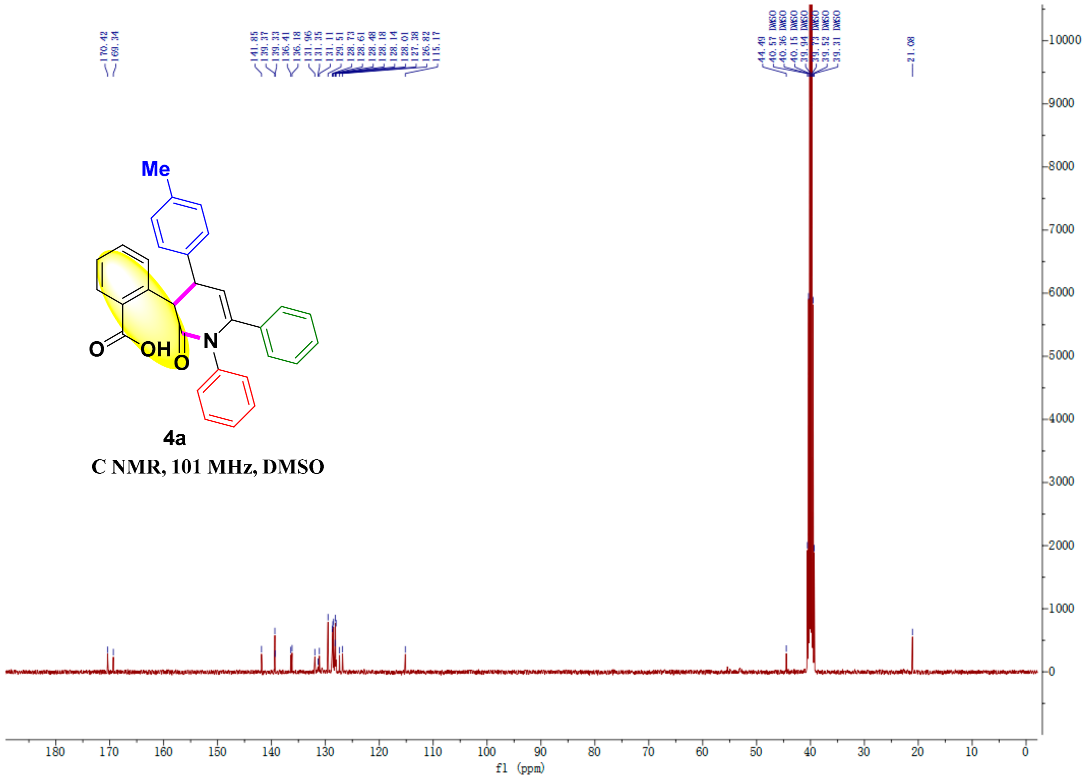
Me
OH
O
N
4a
C NMR, 101 MHz, DMSO
f1 (ppm)

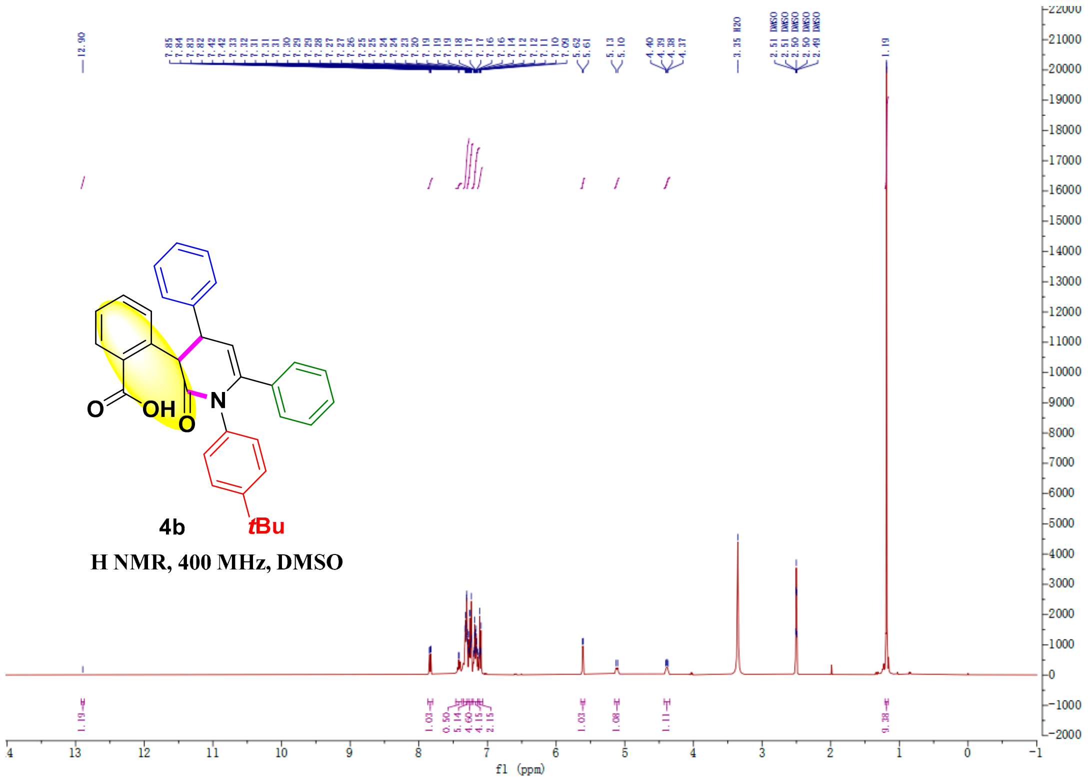

OH
O
O
N
4b
tBu
H NMR, 400 MHz, DMSO
f1 (ppm)

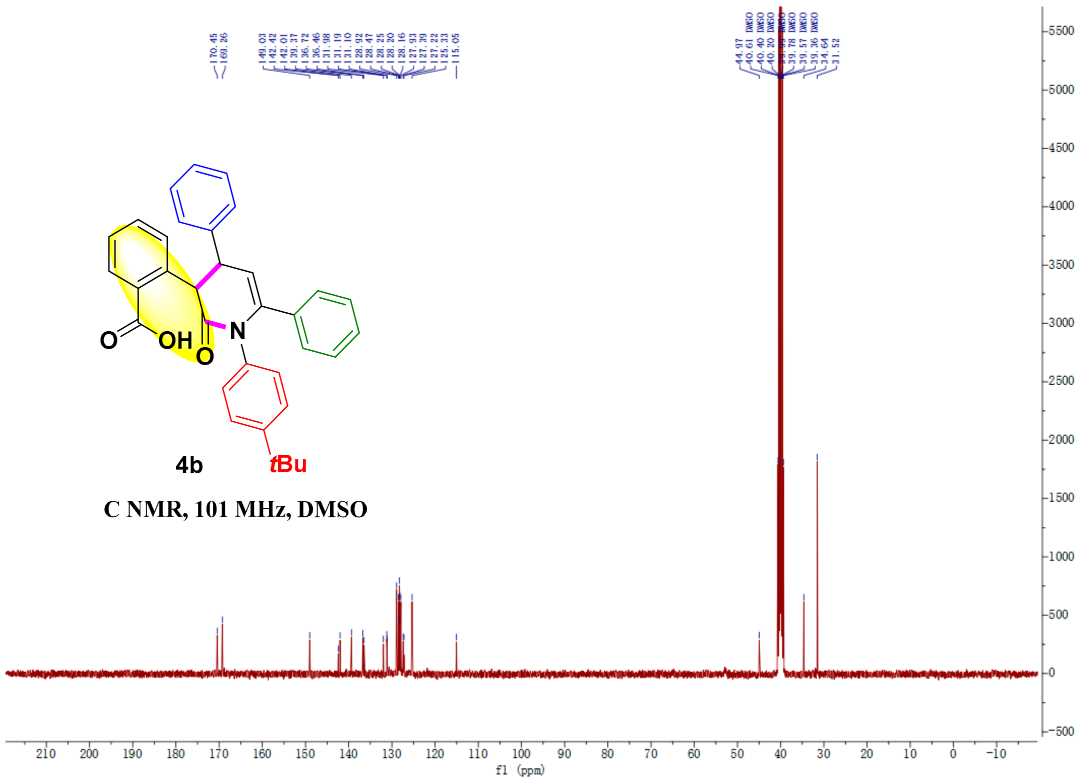

OH
O
O
N
4b
tBu
C NMR, 101 MHz, DMSO
f1 (ppm)

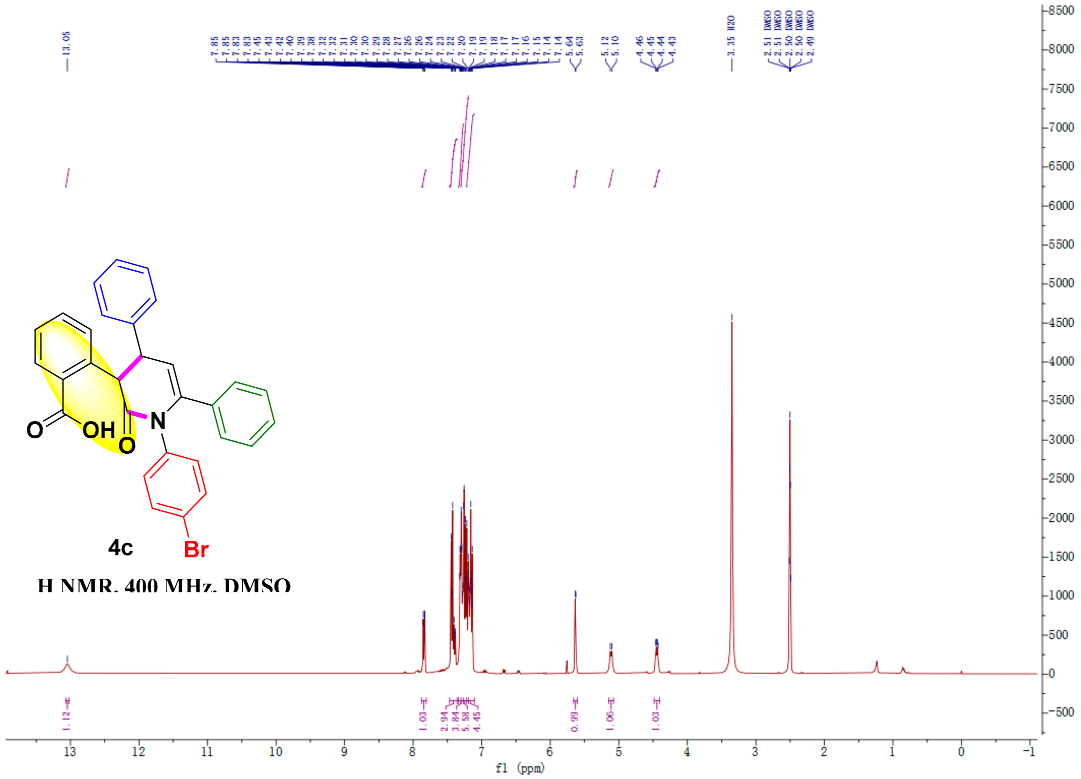
4c
Br
H NMR, 400 MHz, DMSO
f1 (ppm)

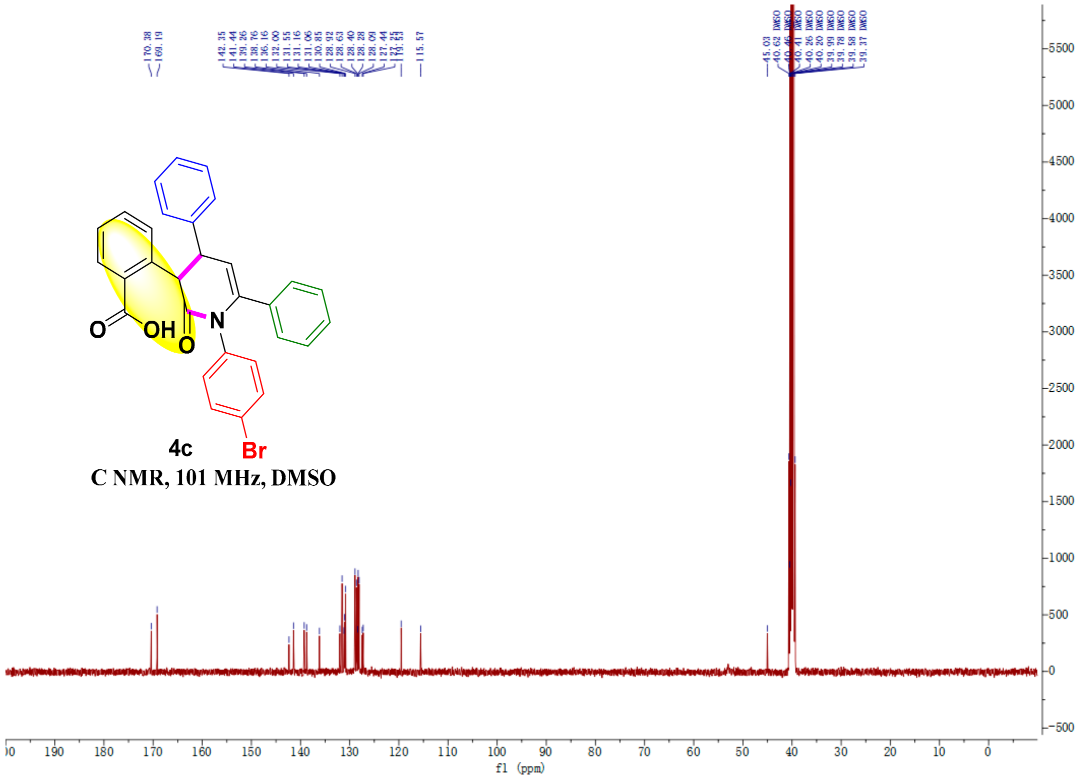
4c
Br
C NMR, 101 MHz, DMSO
f1 (ppm)

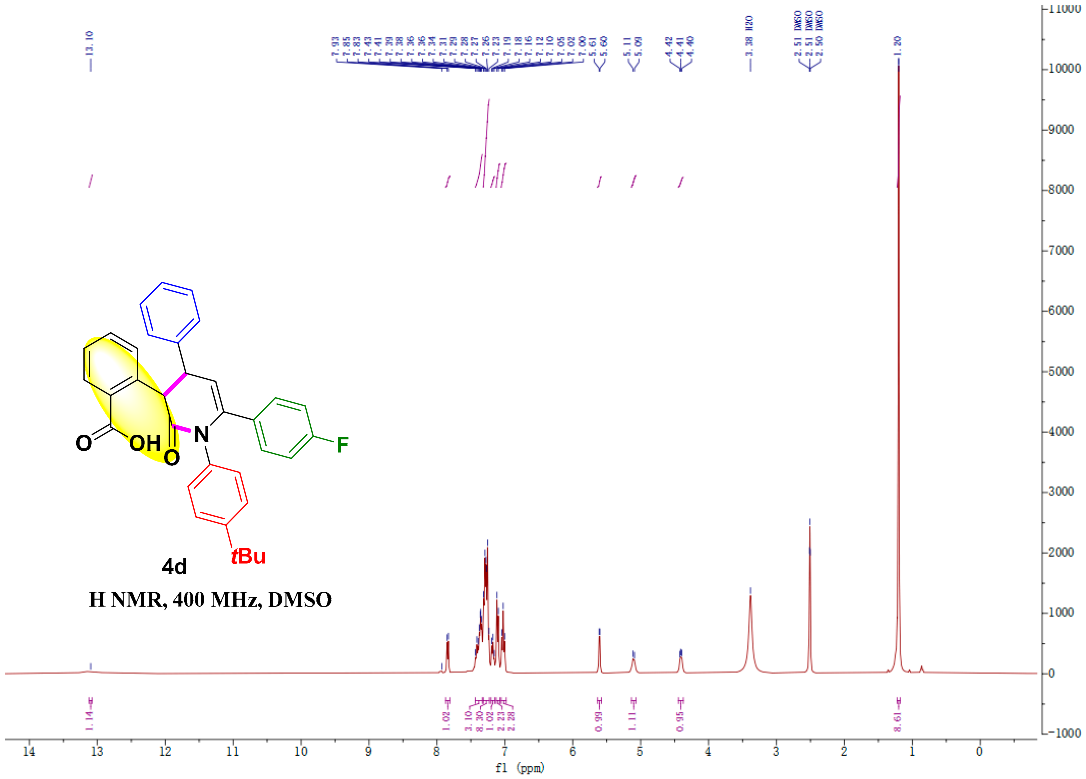
4d
H NMR, 400 MHz, DMSO
O
OH
O
N
F
tBu
13.10
7.93 7.85 7.83 7.43 7.41 7.39 7.38 7.36 7.34 7.31 7.29 7.28 7.27 7.26 7.23 7.19 7.18 7.16 7.12 7.10 7.05 7.02 7.00 5.61 5.60
5.11 5.09
4.42 4.41 4.40
3.38 H2O
2.51 DMSO 2.51 DMSO 2.50 DMSO
1.20
1.14
1.02
3.10 8.30 1.02 2.23 2.28
0.91
1.11
0.95
8.61
f1 (ppm)

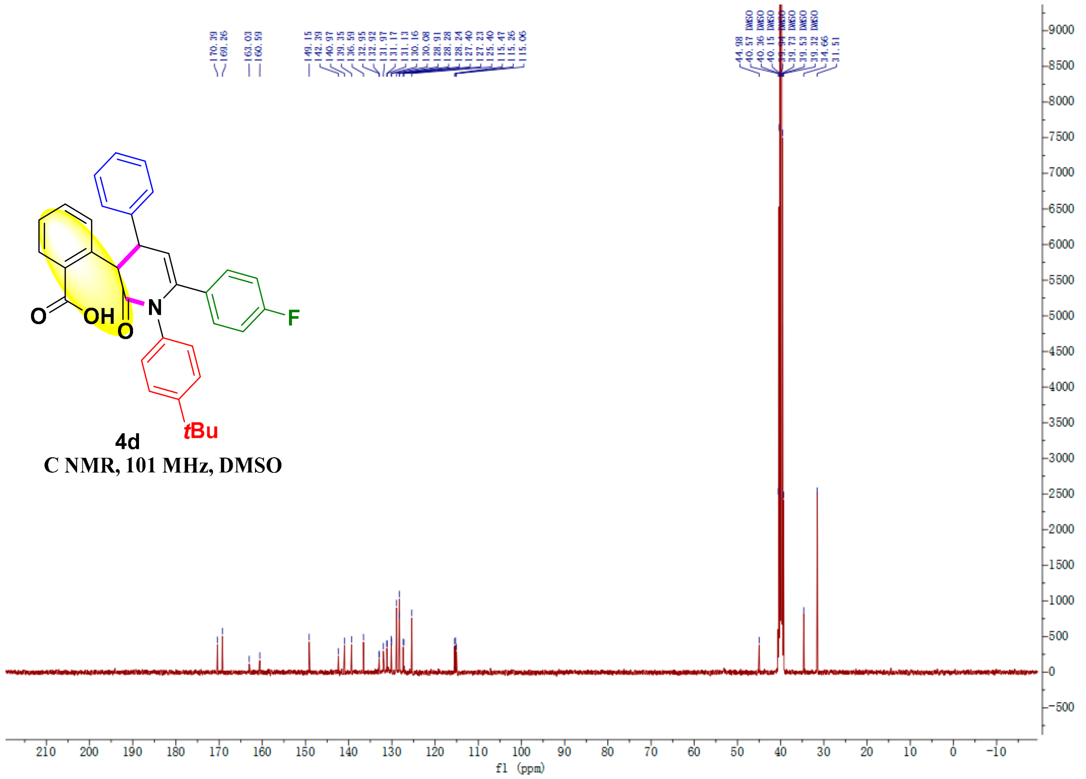
4d
C NMR, 101 MHz, DMSO
O
OH
O
N
F
tBu
170.29 169.26
163.03 160.59
149.15 142.39 140.97 139.25 136.59 132.95 132.92 131.97 131.17 131.13 130.16 130.08 128.91 128.28 128.24 127.40 127.23 125.40 115.47 115.26 115.06
44.98 40.57 DMSO 40.36 DMSO 40.15 DMSO 39.94 DMSO 39.73 DMSO 39.53 DMSO 39.32 DMSO 34.66 31.51
f1 (ppm)

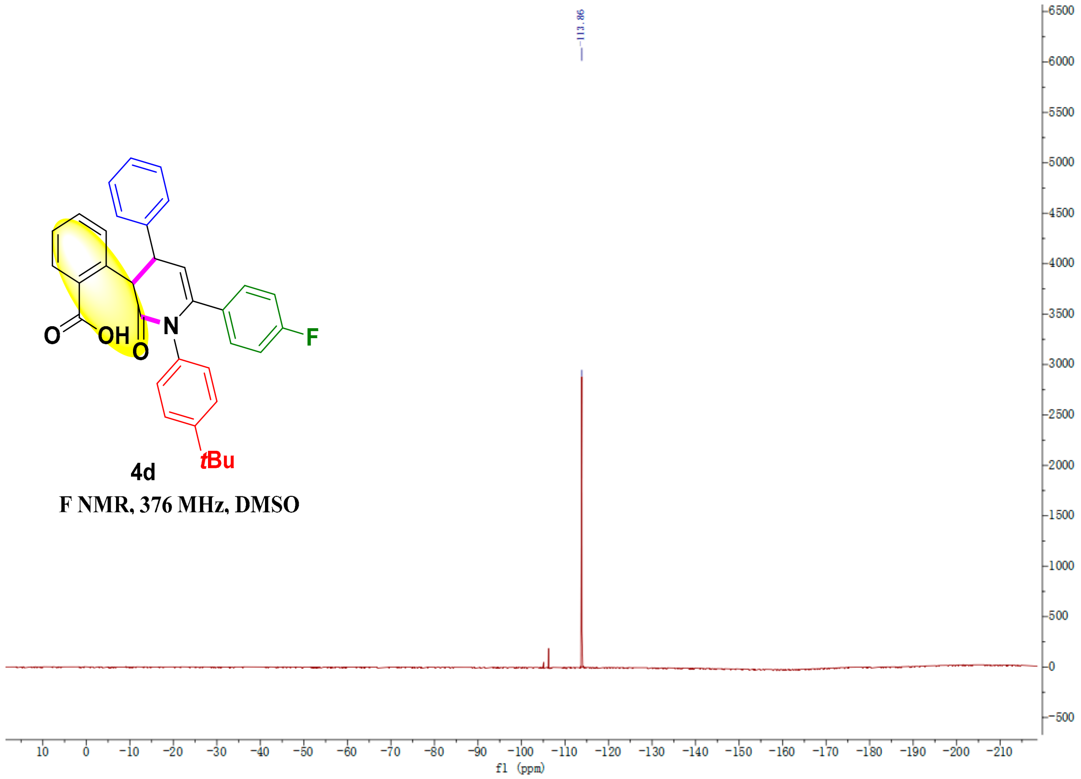

—113.86
O
OH
O
N
F
tBu
4d
F NMR, 376 MHz, DMSO
f1 (ppm)

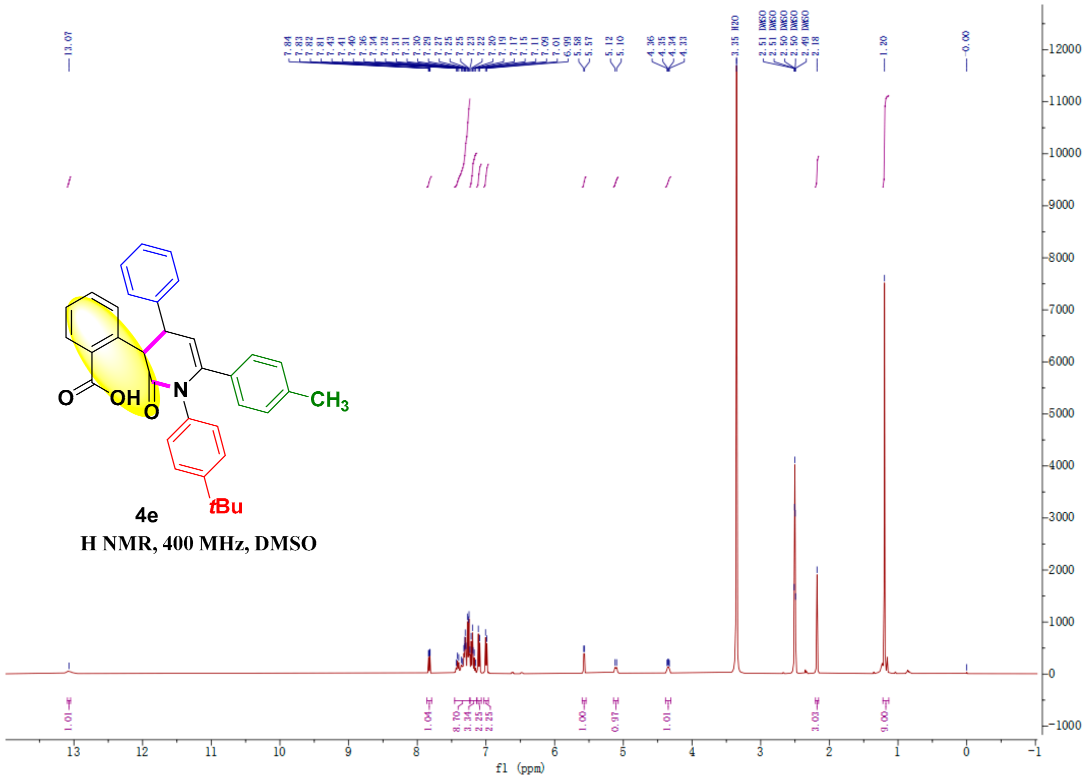

O
OH
O
N
CH3
tBu
4e
H NMR, 400 MHz, DMSO
3.35 H2O
f1 (ppm)

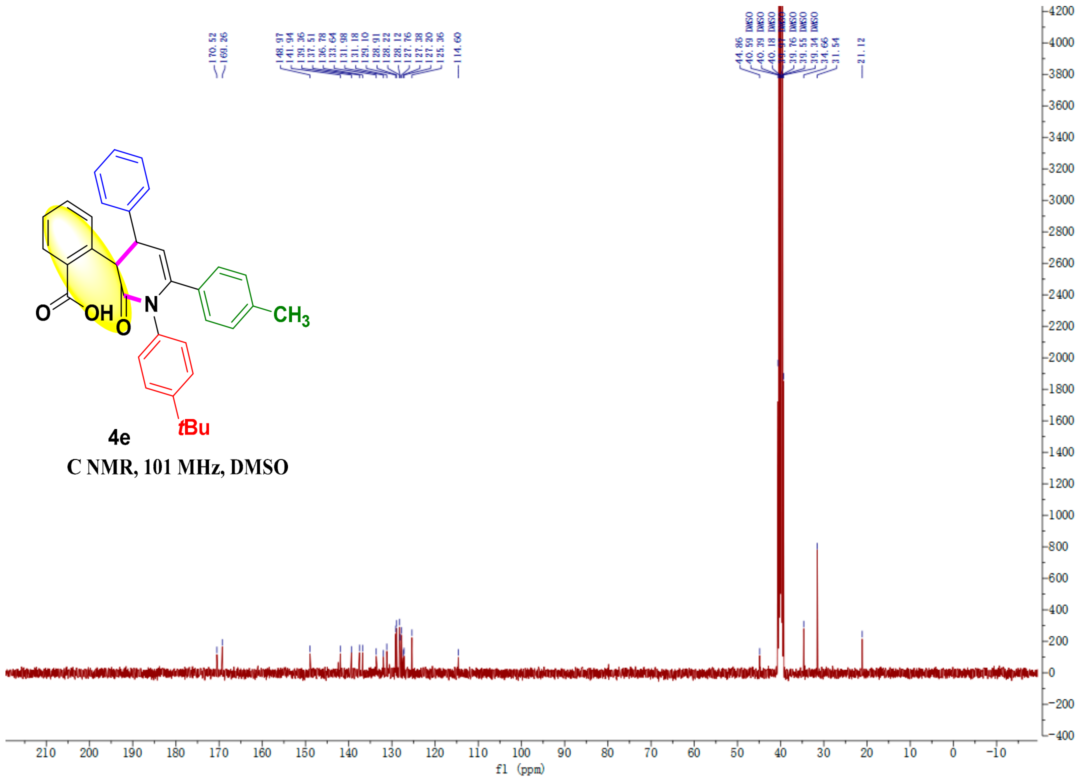
170.52
169.36
148.97
141.94
139.36
137.51
136.78
133.64
131.98
131.18
129.10
128.91
128.22
128.12
127.76
127.38
127.20
125.36
114.60
44.86 DMSO
40.59 DMSO
40.39 DMSO
40.18 DMSO
39.97 DMSO
39.76 DMSO
39.55 DMSO
39.34 DMSO
34.66
31.54
21.12
O
OH
O
N
CH_3
*t*Bu
4e
C NMR, 101 MHz, DMSO
f1 (ppm)

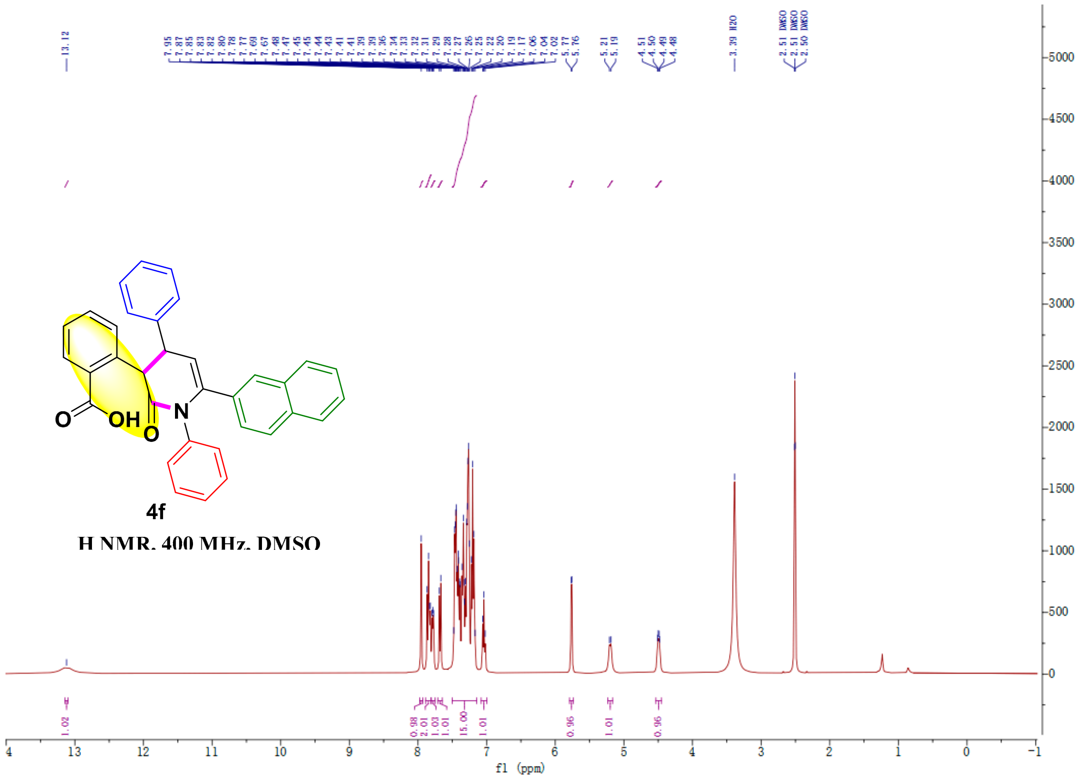
13.12
3.39 H2O
2.51 DMSO
2.51 DMSO
2.50 DMSO
O
OH
O
N
4f
H NMR, 400 MHz, DMSO
f1 (ppm)

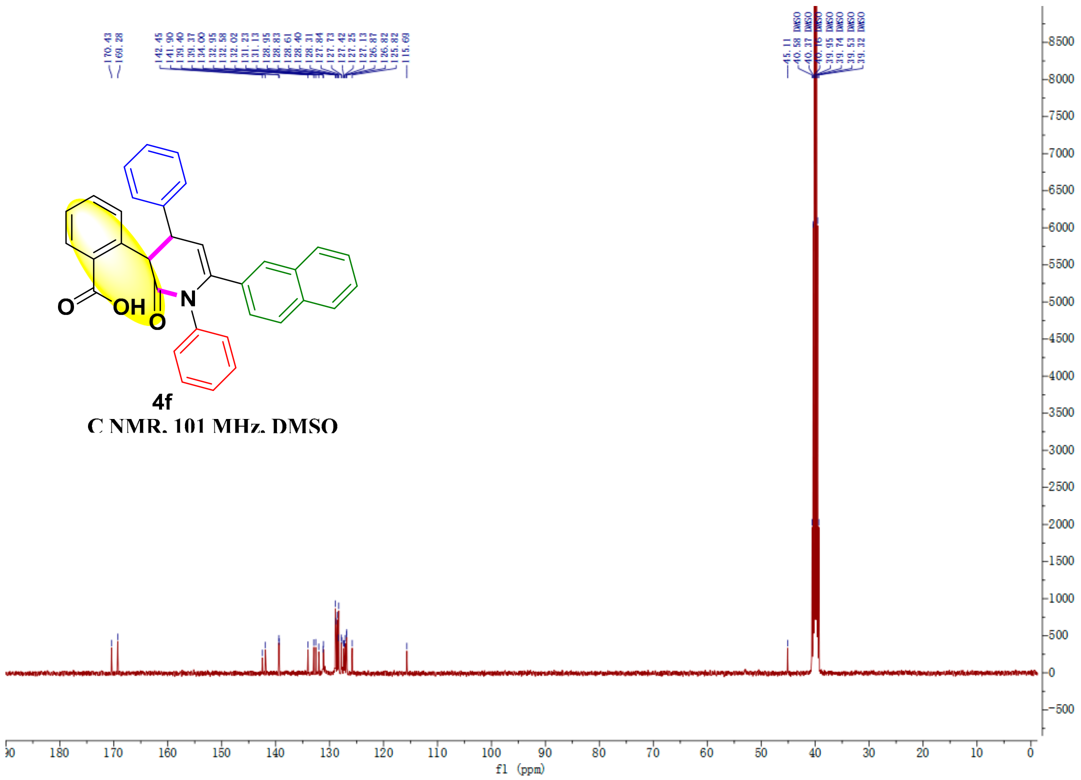
O
OH
O
N
4f
C NMR, 101 MHz, DMSO
f1 (ppm)
